# Mechanofluorochromic Properties of 1,4-Diphenylanthracene Derivatives with Hypsochromic Shift

**DOI:** 10.3390/molecules29020407

**Published:** 2024-01-14

**Authors:** Fumihiro Kannen, Tadatoshi Adachi, Manato Nishimura, Kenji Yoza, Takahiro Kusukawa

**Affiliations:** 1 Faculty of Molecular Chemistry and Engineering, Graduate School of Science and Technology, Kyoto Institute of Technology, Matsugasaki, Sakyo-ku, Kyoto 606-8585, Japan; 2 Bruker Japan K.K., 3-9 Moriya-cho, Kanagawa-ku, Yokohama 221-0022, Japan

**Keywords:** mechanochromism, hypsochromic shift, anthracene, PMMA film

## Abstract

Several types of 1,4-diphenylanthracene derivatives **1**–**4** were prepared, and their photophysical properties were observed in the solid and solution states. Interestingly, the CN-group-substituted 1,4-diphenylanthracene derivative **2** was found to exhibit a higher fluorescence quantum yield (*ϕ*_f_ = 0.71) in the solid state than in the solution state, probably due to the formation of an intermolecular Ar–CN⋯H–Ar hydrogen bond and antiparallel type locked packing structure in the solid state. Furthermore, for some derivatives, an increase in the fluorescence quantum yield was observed in the PMMA film (1 wt%) over both the solid state and the solution state. More interestingly, some of the 1,4-diphenylanthracene derivatives exhibited unusual mechanofluorochromic properties with a “*hypsochromic shift*” in luminous color depending on the substituents of the phenyl group, and with the derivatives having CF_3_, OMe, CN, and two F substituents (**1d**–**1f**, **2**–**4**) showing a significant luminous color change with a “*hypsochromic shift*” after grinding. However, no change in the luminous color was observed for the derivatives having H, Me, and one F substituent (**1a**–**1c**), and especially for some of the CN-substituted derivatives, a reversible luminous color change with a “*hypsochromic shift*” was observed, probably due to the formation of an antiparallel type packing structure. These “*hypsochromic*” anthracene derivatives could probably be utilized as new mechanofluorochromic materials.

## 1. Introduction

Organic solid-state light-emitting materials have received significant attention for their applications in light-emitting diodes (LEDs) [1,2,3,4,5,6,7,8], lasers [9,10,11,12,13,14], and optical waveguides [15,16,17,18,19]. Mechanofluorochromic materials, which are solid organic light-emitting materials that change their luminous color upon mechanical stimuli by crushing or grinding, have attracted attention for their applications in security inks, memory chips, and pressure sensors [20,21,22,23,24,25,26,27,28,29,30]. Numerous organic mechanofluorochromic materials have been reported, including metal complexes [31,32,33,34,35,36,37,38,39,40,41,42,43,44], anthracene derivatives [45,46,47,48,49,50,51,52,53,54,55,56,57,58,59,60,61,62,63,64,65], pyrene derivatives [66,67,68,69,70,71,72,73,74,75,76], tetraphenylethylene derivatives [77,78,79,80,81,82,83], and organoboron compounds [84,85,86,87,88,89,90,91,92,93,94]. However, most of these mechanofluorochromic materials have complex molecular structures and involve a multi-step synthesis, so the practical application of mechanofluorochromic materials with simple structures and easy synthesis is an important issue. Additionally, most of these mechanofluorochromic materials show a “*bathochromic shift*” (red shift) of their luminous color upon grinding, and mechanofluorochromic materials that show a “*hypsochromic shift*” (blue shift) in their luminous color are relatively rare [67,87,95,96,97,98,99,100,101,102,103,104,105,106,107,108,109,110].

Recently, our research group reported that structurally simple 1,8-diphenylanthracene derivatives exhibit characteristic mechanofluorochromic properties with the normal “*bathochromic shift*” [62]. Interestingly, the core anthracene itself does not exhibit any mechanofluorochromic properties at room temperature, but the introduction of phenyl substituents causes it to exhibit mechanofluorochromic properties [65]. In order to develop this structurally simple mechanofluorochromic material and to clarify the reason for the role of the substituted phenyl groups and their substitution positions (i.e., whether or not the substitution at the 1,8-positions of the phenyl group is essential), we synthesized 1,4-diphenylanthracene derivatives **1**–**4** and studied several mechanofluorochromic properties. For the mechanofluorochromic properties of the 1,4-diphenylanthracene derivatives **1a** (R = H), **1b** (R = Me), and **1c** (R = F), the blue luminous color of the pristine sample did not change after grinding. Interestingly, however, “*hypsochromic shift*” of the luminous color changes (e.g., yellow-green to blue-green) was observed after grinding for derivatives **1e**, **1f**, and **2**–**4** having OMe, CF_3_, CN, and two F groups. Especially the 1,4-diphenylanthracene derivatives **1f** (R = CN), **2** (3-CN), and **3** (3,5-CN_2_, R′ = CN) substituted with CN groups underwent repeated reversible luminous color changes with “*hypsochromic shift*” upon grinding, heating, and fuming with solvent vapors, although some of the derivatives could not be fully recovered. This unique “*hypsochromic shift*” with repeated reversible emission color changes is thought to be controlled by the hydrogen bonding between the Ar–CN and Ar–H groups (Ar–CN⋯H–Ar) and the formation of an antiparallel type packing structure. These unique “*hypsochromic*” anthracene derivatives could probably be applied as new mechanofluorochromic materials.

## 2. Results and Discussion

### 2.1. Synthesis and Solid-State Photophysical Properties of 1,4-Diphenylanthracene Derivatives

**Scheme 1:**
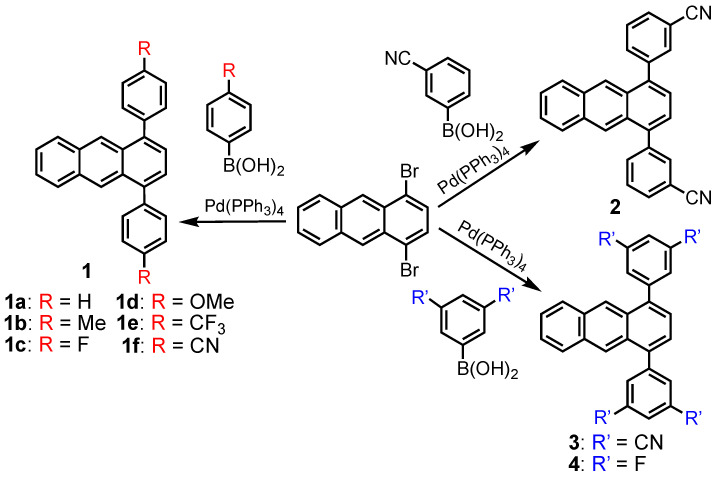
Synthetic route of 1,4-diphenylanthracene derivatives **1**–**4** (Red is substituent R and blue is substituent R’).

The 1,4-diphenylanthracene derivatives **1**–**4** were prepared from 1,4-dibromoanthracene and the corresponding phenylboronic acids using the Suzuki–Miyaura cross-coupling reaction catalyzed by Pd(PPh_3_)_4_ (Scheme 1), and all the derivatives were obtained in 45–99% yields (see experimental part).

**Figure 1:**
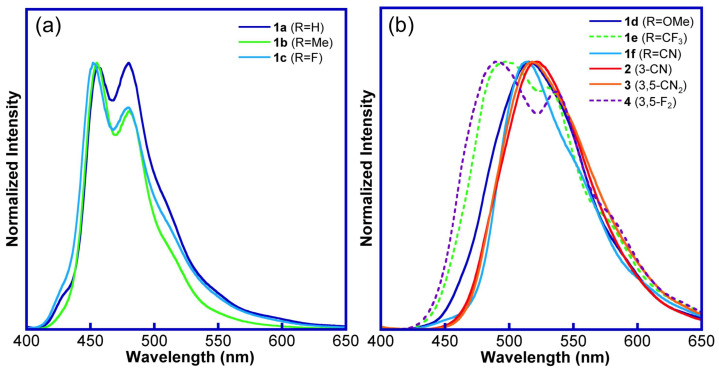
Fluorescence spectra of 1,4-diphenylanthracene derivatives (pristine solids, *λ*_ex_ = 365 nm). (**a**) 1,4-diphenylanthracene derivatives **1a**–**1c** (LB), (**b**) 1,4-diphenylanthracene derivatives **1d**–**1f**, **2**–**4** (YG: solid line, G: dotted line).

The photophysical properties of the obtained 1,4-diphenylanthracene derivatives (**1**–**4**) in the solid and solution state are shown in Table 1, Figures S1, S2, and S15, and the absorption spectra are shown in Figures S13 and S14. All the synthesized 1,4-diphenylanthracene derivatives **1**–**4** showed a light-blue (LB), green (G), or yellow-green (YG) luminous color in the pristine solid state (*λ*_max_ = 452–536 nm) under UV irradiation at 365 nm (Table 1, Figure S2). For the solid-state fluorescence spectra of the pristine samples of the 1,4-diphenylanthracene derivatives, the fluorescence bands around 455–480 nm (*λ*_max_) were observed in the light-blue luminous solids (**1a**–**1c**, LB, Table 1, Figure 1a, Figures S1 and S2). On the other hand, in the yellow-green luminous solids (**1d**, **1f**, **2**, **3**, YG), the fluorescence bands were observed around 514–522 nm (*λ*_max_). Additionally, for the green luminous solids (**1e**, **4**, G), two fluorescence bands were observed around 480 and 530 nm (*λ*_max_, Table 1, Figure 1b, Figures S1 and S2).

The fluorescence quantum yields (*Φ*_f_) of the 1,4-diphenylanthracene derivatives were 0.15–0.71 in the pristine solids, and a relatively high fluorescence quantum yield was observed for **2** (3-CN, *Φ*_f_ = 0.71). The fluorescence lifetimes of the light-blue luminous crystals of **1a**–**1c** are 2.27–3.96 ns, indicating that the short lifetimes are probably due to the monomer emission of the diphenylanthracene part (Table 1, Figure S20). On the other hand, slightly longer fluorescence lifetimes (4.69–5.22 ns) were observed for the green luminous crystals (**1e**, **4**). Additionally, much longer fluorescence lifetimes (7.39–109 ns) were observed for the yellow-green luminous crystals (**1f**, **2**, **3**), and these greenish emission bands are probably due to the excimer emission of the diphenylanthracene part [47,48,51,61,62,63,64,65].

**Figure 2:**
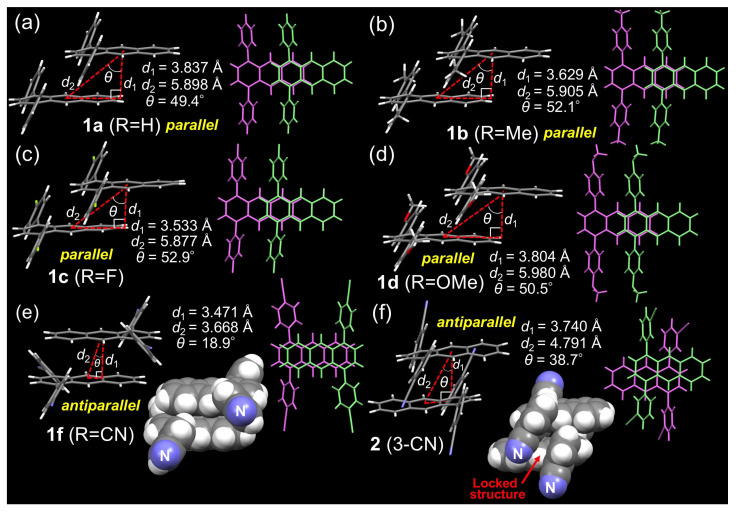
Crystal structures of the 1,4-diphenylanthracene derivatives: (**a**) **1a** (R = H), (**b**) **1b** (R = Me), (**c**) **1c** (R = F), (**d**) **1d** (R = OMe), (**e**) **1f** (R = CN), (**f**) **2** (3-CN).

To understand the relationship between the crystal packing and solid state photophysical properties (fluorescence quantum yield, fluorescence lifetime, and luminous color) of the 1,4-diphenylanthrcene derivatives, the single crystal structures of **1a** (R = H, light-blue luminous color, LB), **1b** (R = Me, light-blue luminous color, LB), **1c** (R = F, light-blue luminous color, LB), **1d** (R = OMe, yellow-green luminous color, YG), **1f** (R = CN, yellow-green luminous color, YG), and **2** (3-CN, yellow-green luminous color, YG) were analyzed. Single crystals were obtained from dimethoxyethane (DME) solutions for **1d** and **1f**, from hexane solutions for **1a** and **1c**, from a CHCl_3_ solution for **2**, and from a CH_2_Cl_2_ solution for **1b**. For the bimolecular stacked crystal packing of **1a** (R = H), **1b** (R=Me), **1c** (R = F), and **1d** (R = OMe), the 1,4-diphenylanthracene rings are arranged in the same direction, called the “parallel orientation” (Figure 2). On the other hand, in the bimolecular stacked crystal packing of the CN-group substituted derivatives **1f** (R = CN) and **2** (3-CN), the 1,4-diphenylanthracene rings are arranged in the “antiparallel orientation”, and a stronger *π*-stacking is observed than in the “parallel orientation” (Figure 2). Additionally, Ar–CN⋯H–Ar or Ar–F⋯H–Ar hydrogen bonds were observed in the crystal packing of **1c** (R = F), **1f** (R = CN), and **2** (3-CN) with CN or F substituents (Figures S28, S32 and S34). To understand the reason for the formation of the “parallel” and “antiparallel” orientation packings of the 1,4-diphenylanthracene rings in the crystal structure, the electrostatic potential surfaces were calculated using DFT calculations according to the obtained crystal data (Figures S41 and S42). For the CN-group substituted derivatives **1f** (R = CN) and **2** (3-CN), the positive charges are located at the edge of the substituted phenyl ring, as shown in Figures S41 and S42, and these positively charged edges are stacked with the center part of the phenyl ring of the other molecules to form a stable stacking bimolecular “antiparallel” packing. Additionally, in the crystal structure of the bimolecular packing of **1f** (R = CN) and **2** (3-CN), many interactions, such as CH–*π* interactions between two 1,4-diphenylanthracen rings, were observed compared to the other non-CN substituted derivatives (**1a**–**1d**), as shown in Figure S36, and it may be showing that the “antiparallel” orientation is more stable than the “parallel” orientation in the case of electron withdrawing CN-group substituted derivatives.

**Figure 3:**
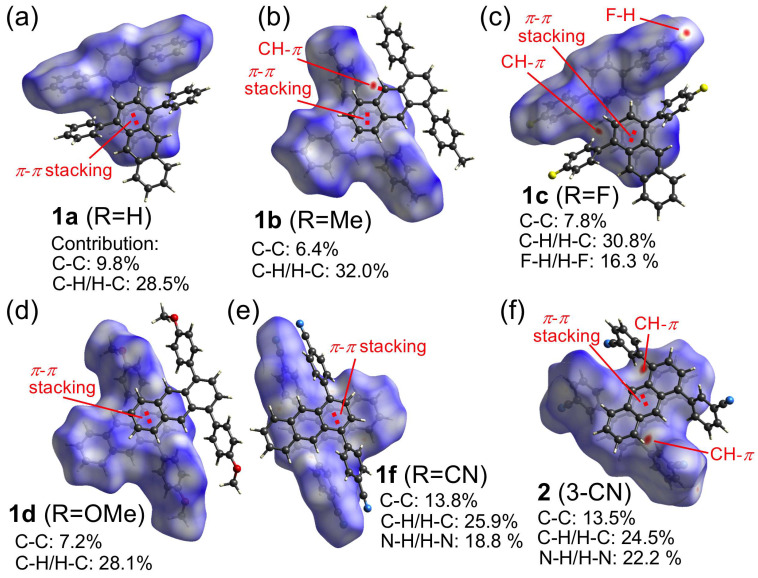
Hirshfeld surface showing the intermolecular interactions over *d*_norm_ and the percentages of the contact’s contribution to the total Hirshfeld surface area of the molecules: (**a**) **1a** (R = H), (**b**) **1b** (R = Me), (**c**) **1c** (R = F), (**d**) **1d** (R = OMe), (**e**) **1f** (R = CN), (**f**) **2** (R′ = CN, 3-CN).

According to the crystal structure analysis and photophysical data of the 1,4-diphenylanthracene derivatives, we can discuss the following:

(1) *Relation between solid-state luminous color, crystal packing, Hirshfeld surface analysis, and fluorescence lifetimes*: As already mentioned, the luminous colors of the pristine samples of the 1,4-diphenylanthracene derivatives show light-blue (**1a**–**1c**, LB), green (**1e**, **4**, G), and yellow-green (**1d**, **1f**, **2**, **3**, YG) colors. In the case of the light-blue luminous derivatives **1a**–**1c**, the emission is probably derived from the monomer of the 1,4-diphenylanthracene rings since the crystal packing of **1a**–**1c** showed only partial overlapping of the anthracene ring, as indicated by the purple and green colors in Figure 2a,c. On the other hand, in the case of the yellow-green luminous derivatives **1f** (R = CN) and **2** (3-CN), the emission is probably derived from the excimer state of the 1,4-diphenylanthracene rings, and a greater overlapping of the anthracene rings compared to **1a**–**1c** is observed, as shown in Figure 2e,f. In the case of the yellow-green luminous MeO-group substituted derivative **1d**, the crystal packing is very similar (overlapping only one benzene ring) to the light-blue luminous derivatives **1a**–**1c,** as shown in Figure 2a–d, suggesting that an intermolecular charge transfer may be operating (see Figures S39 and S40, DFT calculation) and showing a yellow-green luminous color. To further understand the contribution of the *π*–*π* stacking for the crystal packing, the Hirshfeld surface analysis according to the crystal data was performed (Figure 3). The contribution of *π*–*π* stacking for **1f** (R = CN, contribution of the C–C interactions: 13.8%) and **2** (3-CN, contribution of the C–C interactions: 13.5%) is greater than that for **1a**–**1c** (contribution of the C–C interactions: 6.4–9.8%), indicating that the yellow-green emission of **1f** and **2** is due to the formation of the *π–π* stacking (excimer emission). The fluorescence lifetimes of the 1,4-diphenylanthracene derivatives for the pristine state also show the difference in the contribution of the *π–π* stacking interactions as follows. Short fluorescence lifetimes between 2.27–3.96 ns were observed for the light-blue luminous samples **1a**–**1c**; however, slightly longer fluorescence lifetimes were observed for the green luminous samples **1e** (4. 69 ns, R = CF_3_) and **4** (5.22 ns, R′ = F, 3,5-F_2_) and for the yellow-green luminous samples **1f** (R = CN), **2** (3-CN) and **3** (R ′= CN, 3,5-CN_2_), longer fluorescence lifetimes were observed between 7.39 and 109 ns (Table 1, Figure S20). These observed fluorescence lifetimes are increasing in the order of light-blue < green < yellow-green luminous color (the fluorescence wavelengths also increased in the same order). These results indicated that the contribution of the *π–π* stacking interactions is also increasing in the same order, which is in good agreement with the crystal data only except for **1d**.
(2) *Relation between fluorescence quantum yield, fluorescence lifetime, and crystal packing of **1f** (R=CN) and **2** (3-CN)*: The pristine sample of **2** (3-CN) exhibited a relatively high fluorescence quantum yield (*ϕ*_f_ = 0.71) compared to the other 1,4-diphenylanthracene derivatives (*ϕ*_f_ = 0.15–0.44, Table 1), as already described. To consider the reason for the higher fluorescence quantum yield of **2** (3-CN), we calculated the rate constant *k*_f_ for the fluorescence and the rate constant *k*_nr_ for non-radiative decay (Table S1) and compared *k*_f_ and *k*_nr_ to that of **1f** (R = CN) and **2** (3-CN). The rate constants *k*_f_ for the fluorescence of **1f** (*k*_f_ = 0.008 ns^−1^) and **2** (*k*_f_ = 0.006 ns^−1^) were almost the same, but the rate constant *k*_nr_ for the non-radiative decay of **2** (*k*_nr_ = 0.003 ns^−1^) was about seven times lower than that of **1f** (*k*_nr_ = 0.022 ns^−1^), and the difference in *k*_nr_ caused the fluorescence quantum yield of **2** (*ϕ*_f_ = 0.71) to be higher than that of **1f** (*ϕ*_f_ = 0.26) in the pristine solid state. The difference in *k*_nr_ between **1f** and **2** may be due to the difference in the crystal packing structure. Due to the substitution of a CN group at the 3-position (meta-position) of the phenyl group in the derivative **2** and the intermolecular hydrogen bond (Ar–CN⋯H–Ar) formed by the CN group, the crystal packing structure of **2** forms a tight, lock-and-key type packing compared to the loose packing structure of **1f** (Figure 2e,f and Figure S37). The tight packing structure of **2** is considered to suppress the molecular vibration in the solid state compared to the packing structure of **1f** and exhibits a very low *k*_nr_ (0.003 ns^−1^ for **2**) and a high fluorescence quantum yield (*ϕ*_f_ = 0.71).

### 2.2. Photophysical Properties of 1,4-Diphenylanthracene Derivatives in Solution and in PMMA Films

To compare the photophysical properties of the 1,4-diphenylanthracene derivatives in the solid and solution states, we measured their UV spectra, fluorescence spectra, fluorescence quantum yields, and fluorescence lifetimes of the 1,4-diphenylanthracene derivatives in chloroform solutions (Table 1, Figures S14–S16 and S21). In the case of 1,4-diphenylanthracene derivatives **1a**–**1c**, **1e**, **2,** and **4**, a blue luminous color (421–443 nm) was observed in the CHCl_3_ solution. However, for derivatives **1d** (R = OMe), **1f** (R = CN), and **3** (3,5-CN_2_), which have electron-donating and electron-withdrawing groups, a light-blue luminous color (454–458 nm) was observed in the CHCl_3_ solution (Figures S15 and S16). These derivatives may be causing a charge-transfer interaction in the solution state, but only a small solvatochromic effect on the fluorescence spectra was observed in different solvents (Figure S17). For all the 1,4-diphenylanthracene derivatives, the fluorescence quantum yields were observed with a moderate efficiency around *ϕ*_f_ = 0.41–0.56, and for almost all derivatives, the CHCl_3_ solution state showed higher fluorescence quantum yields than the pristine solid state. Additionally, the fluorescence lifetimes were observed around *τ* = 2.57–3.36 ns for all the derivatives, probably indicating a monomer emission in the solution state. Interestingly, only derivative **2** (3-CN) showed a higher fluorescence quantum yield (*ϕ*_f_ = 0.71) in the pristine solid state than in the solution state (*ϕ*_f_ = 0.46 in CHCl_3_).

In order to develop new fluorescent materials, it is important that the newly synthesized fluorescent materials exhibit high fluorescence properties even in the polymer-supported solid state. Therefore, we measured the photophysical properties of the 1,4-diphenylanthracene derivatives in PMMA (polymethyl methacrylate). The desired 1,4-diphenylanthracene derivative-containing PMMA film (1 wt%) was prepared by casting on a glass plate using a CHCl_3_ solution. For most of the PMMA films obtained, a blue or light-blue luminous color was observed, and the fluorescence spectra was similar to that of the CHCl_3_ solution but was significantly different from that of the pristine solid (Figures S18 and S19). These observations indicated that the blue emission of the 1,4-diphenylanthracene derivatives in the PMMA films is derived from the monomer emission, which is a significant difference from the solid-state emission (Figure S18). The fluorescence quantum yields of the PMMA films (1 wt%) of the 1,4-diphenylanthracene derivatives show relatively high efficiencies around *ϕ*_f_ = 0.62–0.71. Interestingly, the fluorescence quantum yields in the PMMA films are increased compared to the solid and solution states, except for **2** (3-CN). Additionally, the fluorescent lifetime of the PMMA film (1 wt%) of the 1,4-diphenylanthracene derivatives exhibited a slightly longer lifetime (*τ* = 3.45–4.16 ns) than the solution state (Tables S1 and S2, Figure S22). Based on these photophysical measurements, the rate constant *k*_f_ for fluorescence of the PMMA films (*k*_f_ = 0.1425–0.2058) was found to be similar to that of the corresponding solution state (Tables S1 and S2). However, the rate constants *k*_nr_ for non-radiative decay of the PMMA film (*k*_nr_ = 0.0837–0.0936) was found to be much lower than for the solution and solid states (Tables S1 and S2). The lower *k*_nr_ value showed the inhibition of the molecular motion in the PMMA film and showed an increasing fluorescence quantum yield in the PMMA films than in the solution state. In the pristine solid state of the 1,4-diphenylanthracene derivatives, the aggregation-caused quenching (ACQ) effect that may have occurred and the fluorescence quantum yields were reduced, except for derivative **2** (3-CN).

### 2.3. Mechanofluorochromic Properties of 1,4-Diphenylanthracene Derivatives

**Figure 4:**
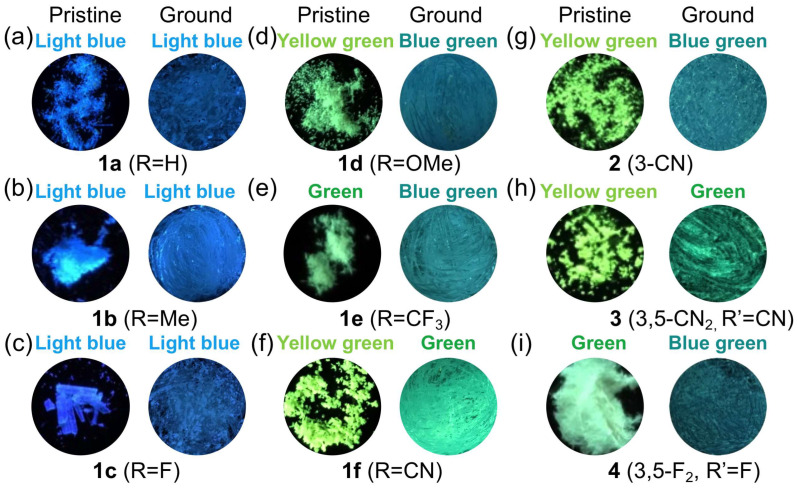
Fluorescent images of the pristine and ground samples of 1,4-diphenylanthracene derivatives **1**–**4** during a 365 nm UV irradiation: (**a**) **1a** (R = H), (**b**) **1b** (R = Me), (**c**) **1c** (R = F), (**d**) **1d** (R = OMe), (**e**) **1e** (R = CF_3_), (**f**) **1f** (R = CN), (**g**) **2** (3-CN), (**h**) **3** (3,5-CN_2_, R′ = CN), (**i**) **4** (3,5-F_2_, R′ = F).

**Figure 5:**
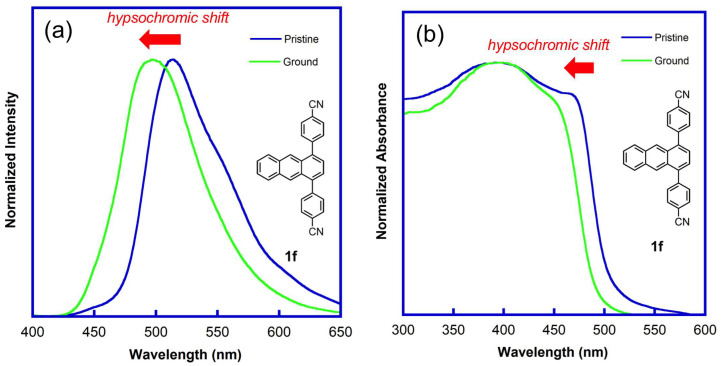
Fluorescence and UV-vis spectra of 1,4-diphenylanthracene derivative **1f** (R=CN) in the solid state: (**a**) fluorescence spectra of the pristine and ground samples (*λ*_ex_ = 365 nm), (**b**) UV-vis absorption spectra of the pristine and ground samples.

To further investigate the fluorescence properties of these 1,4-diphenylanthracene derivatives, we studied their mechanofluorochromic properties in the solid state. The light-blue luminous color (*λ*_max_ = 452–455, 480 nm) of the pristine samples of the 1,4-diphenylanthracene derivatives **1a** (R = H), **1b** (R = Me), and **1c** (R = F) did not change their luminous color after grinding in a mortar at room temperature under 365 nm UV light (Table 1, Figure 4, Figures S1 and S2). On the other hand, the yellow-green or green luminous color of the 1,4-diphenylanthracene derivatives **1d** (R = OMe, from 515 nm YG to 486 nm BG), **1e** (R = CF_3_, from 497, 530 nm G to 488 nm BG), **1f** (R = CN, from 514 nm YG to 497 nm G), **2** (3-CN, from 522 nm YG to 493 nm BG), **3** (3,5-CN_2_, R′ = CN, from 518 nm YG to 497 nm G), and **4** (3,5-F_2_, R′ = F, from 489, 536 nm G to 489 nm BG) changed to shorter wavelength luminous colors (i.e., *hypsochromic shift*) when ground in a mortar (Table 1, Figure 4 and Figure 5a, Figures S1 and S2), and the fluorescence spectra also significantly changed, as shown in Figure S1. The compounds that show a “hypsochromic shift” in fluorescent color after grinding are very rare and interesting, but compared to mechanofluorochromic materials that show a “bathochromic shift”, the mechanistic studies are unclear at the present moment [67,87,95,96,97,98,99,100,101,102,103,104,105,106,107,108,109,110].

For the mechanofluorochromic properties of the 1,8-diphenylanthracene derivatives previously reported, some of the derivatives recovered to their original fluorescent color after grinding within 5 min. at room temperature, but the CN-group substituted derivatives did not recover to their original emission color at room temperature [62]. However, this mechanofluorochromism of the 1,4-diphenylanthracene derivatives was not observed as a self-recovering phenomenon within 5 min. at room temperature in the luminous color changed derivatives **1d**–**1f** and **2**–**4** (Figures S1 and S2).

For the yellow-green (YG) emitting CN-substituted derivatives **1f**, **2,** and **3**, the fluorescence lifetimes (*τ*) of the ground samples became shorter than those of the pristine samples (Table 1), along with a “*hypsochromic shift*” of the luminous color. These results indicated that the *π–π* stacking structure dissociates after grinding, exhibiting a “*hypsochromic shift*” in the luminous color and a shortening of the fluorescence lifetimes.

**Figure 6:**
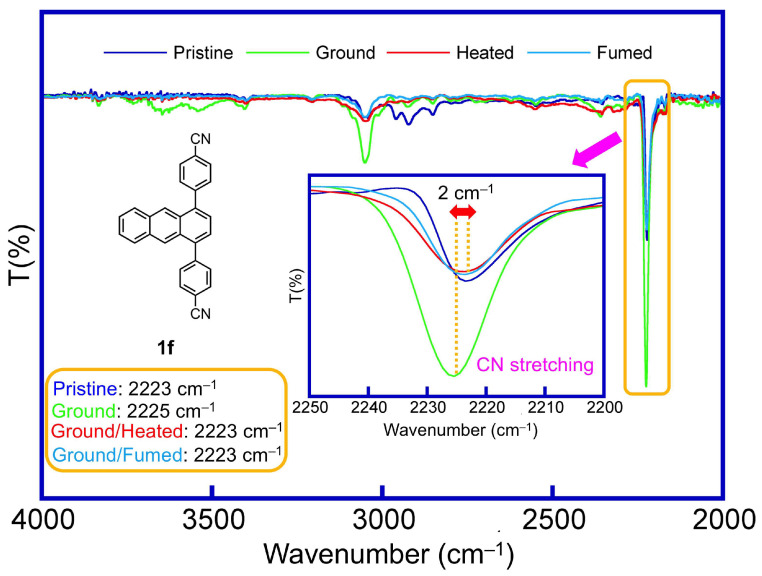
IR spectra of the 1,4-diphenylanthracene derivative **1f** (pristine, ground, ground-heated, ground-fumed samples) in the CN stretching region.

To further understand the mechanofluorochromism of the 1,4-diphenylanthracene derivatives, the UV and IR spectra were observed in the solid state for the pristine and ground samples. In the UV spectra, a blue shift (*hypsochromic shift*) and decreasing of the longer wavelength peaks were observed for the derivatives **1d**–**1f**, **2**–**4** (Figure 5b and Figure S13), which showed a “*hypsochromic shift*” in the fluorescence spectra after grinding, indicating a decrease in the contribution of the *π*-stacking structure after grinding. Furthermore, infrared (IR) spectra of the CN-substituted 1,4-diphenylanthracene derivatives **1f** (R = CN) and **2** (3-CN) before and after grinding were observed, and the CN stretching band of the **1f** and **2** shifted to the higher wavenumber side from the pristine to ground one, indicating that the hydrogen bond (Ar–CN⋯H–Ar) strength decreased after grinding (Figure 6, Figures S10 and S11).

### 2.4. Vapochromic and Thermochromic Properties of 1,4-Diphenylanthracene Derivatives

**Figure 7:**
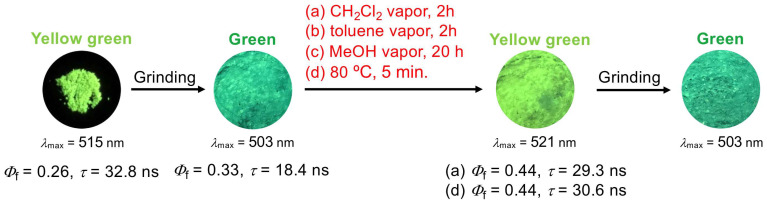
Typical fluorescence images of ground, fumed (CH_2_Cl_2_, toluene, and MeOH vapors), and heated (80 °C for 5 min.) samples of 1,4-diphenylanthracene derivative **1f** (R = CN) upon irradiation by 365 nm UV lamp.

**Figure 8:**
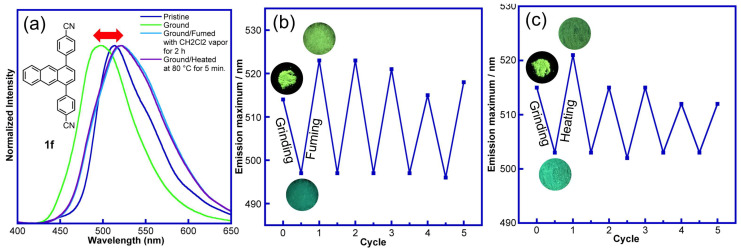
Vapochromic and thermochromic properties of 1,4-diphenylanthracene derivative **1f** (R = CN): (**a**) fluorescence spectra of pristine, ground, ground-fumed (CH_2_Cl_2_ vapor, 2 h), and ground-heated (80 °C, 5 min.) samples, (**b**) repeated grinding–fuming cycles, (**c**) repeated grinding–heating cycles.

To understand the reversibility of the luminous color of the 1,4-diphenylanthracene derivatives, fluorescence images and fluorescence spectra of the ground samples were observed after fuming with the CH_2_Cl_2_ vapor. The blue-green luminescent ground samples of derivatives **1d** (R = OMe), **1e** (R = CF_3_), and **4** (3,5-F_2_, R′ = F) showed no recovery of the luminous color (recovery to green or yellow-green, bathochromic shift) after fuming with the CH_2_Cl_2_ vapor for 20 h (Figures S3 and S4). Interestingly, for the CN-group-substituted 1,4-diphenylanthracene derivatives **1f** (R = CN), **2** (3-CN), and **3** (3,5-CN_2_, R′ = CN), the yellow-green luminous color of the pristine sample turned to the green or blue-green luminous color (“*hypsochromic shift*”) with grinding, and the original yellow-green luminous color was recovered by fuming with solvent vapor (Figure 7, Figures S3 and S4). Additionally, repeated grinding–fuming cycles were also observed, but in some cases, a decrease in the fluorescence intensity and decreasing of the color-switching ability were observed (Figure 8, Figures S5 and S6). To understand the reason for the decrease in the fluorescence intensity and decreasing of the color-switching ability, we measured fluorescence spectra and ^1^H NMR spectra of the derivatives **1a** (R = H) and **2** (3-CN) after UV irradiation in the solid state (365 nm, 5 min.). In the fluorescence spectra of **1a**, no change in fluorescence intensity was observed after 365 nm UV irradiation. On the other hand, a slight decrease in fluorescence intensity was observed for derivative **2**, but no spectral change was observed in the ^1^H NMR spectrum after dissolving the irradiated samples (Figures S43 and S44).

Again, for derivatives **1d** (R = OMe), **1e** (R = CF_3_), and **4** (3,5-F_2_, R′ = F), no recovery to the original luminous color (pristine state) by heating the ground sample was observed (Figures S3 and S4). However, for the CN-group-substituted 1,4-diphenylanthracene derivatives **1f** (R = CN), **2** (3-CN), and **3** (3,5-CN_2_, R′ = CN), a similar recovery of the luminous color of the ground sample to the luminous color of the pristine sample by heating was also observed (Figure 7, Figures S3, S4, and S7–S9). The green luminous ground sample of **1f** (R = CN) turned yellow-green when heated at 80 °C for 5 min., a recovery of the green luminous color was observed upon regrinding (Figure 7 and Figure 8a), and the repeated cycles of grinding–heating are shown in Figure 8c. Although repeated grinding–heating cycles were observed, in some cases, a decrease in the fluorescence intensity and decreasing of the color-switching ability were observed.

**Figure 9:**
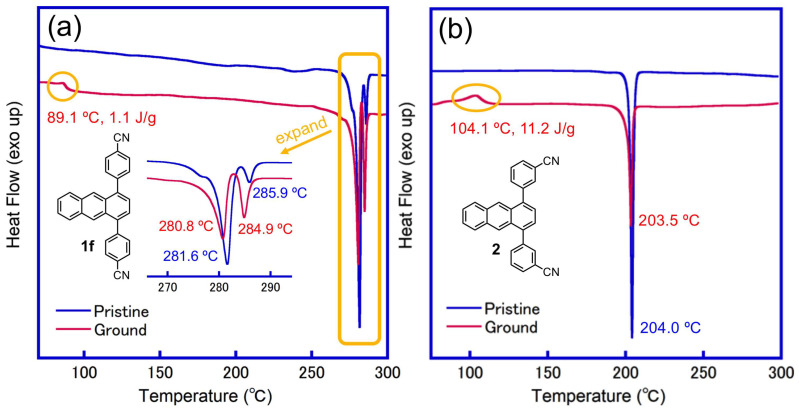
DSC profiles of 1,4-diphenylanthracene derivatives before grinding (pristine) and after grinding (ground): (**a**) **1f** (R = CN), (**b**) **2** (3-CN).

To further understand the grinding–heating process of the CN-substituted 1,4-diphenylanthracene derivative **1f** (R = CN), differential scanning calorimetry (DSC) was conducted to observe the phase transition between the ground and ground-heated samples. For the yellow-green luminous pristine sample of **1f**, only the endothermic peak of melting (281.6 °C, 285.9 °C) was observed (Figure 9a). On the other hand, a broad exothermic peak was observed around 89.1 °C in the green luminous ground sample, which may have caused the transition from the green to yellow-green luminous state. Similar exothermic peaks were also observed in the ground samples for derivative **2** with a substituted CN group (Figure 9b). The DSC analysis of the other 1,4-diphenylanthracene derivatives **1a**–**1e** was examined, but only endothermic melting peaks were observed (Figure S12).

**Figure 10:**
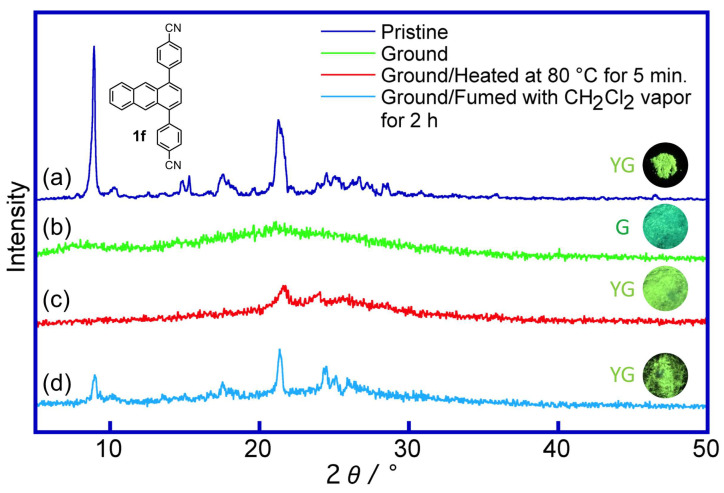
Powder X-ray diffraction (PXRD) patterns of 1,4-diphenylanthracene derivative **1f** (R = CN): (**a**) pristine, (**b**) ground, (**c**) ground-heated sample (80 °C, 5 min.), and (**d**) ground-fumed sample (with CH_2_Cl_2_ vapor, 2 h).

To understand in detail the mechanofluorochromic properties of the 1,4-diphenylanthracene derivatives, the powder X-ray diffraction patterns (PXRD) of the pristine, ground, ground-fumed, and ground-heated samples of derivative **1f** (R = CN) were observed. The “yellow-green” luminous pristine sample of **1f** showed a sharp and strong peak in the PXRD measurement (Figure 10a), but no diffraction peak was observed in the ground “green luminous” sample (Figure 10b). After heating at 80 °C for 5 min., the ground sample recovered its “yellow-green luminous color” and showed small crystallization peaks (Figure 10c). Similar crystallization of the ground sample was also observed when fuming with CH_2_Cl_2_ vapor for 2 h (Figure 10d). These observations, like those in the previously reported paper, show that this mechanofluorochromism may be operating under a phase transition from the crystalline state to the amorphous state [32,33,34,35,36,37,38,39,40,41,42,43,44,46,47,48,49,52,53,55,57,58,59,71,72,77,80,83,84,86,87,89,90,91,92,93,94]. Furthermore, during the vapochromic and thermochromic recovery of the ground sample of **1f**, a reversible change in the fluorescence lifetimes (pristine: *τ* = 32.8 ns, ground: *τ* = 18.4 ns, ground-fumed with CH_2_Cl_2_ vapor: *τ* = 29.3 ns, ground-heated: *τ* = 30.6 ns) was observed (Figure 7). These changes in the fluorescence lifetimes of **1f** are indicated as follows: (i) the *π–π* stacked pristine sample showed a yellow-green luminous color (*λ*_max_ = 514 nm) and showed a longer fluorescence lifetime (*τ* = 32.8 ns) due to *π–π* stacking structure; (ii) after grinding, the sample showed a green luminous color (*λ*_max_ = 497 nm, “*hypsochromic shift*”) and showed a shorter fluorescence lifetime (*τ* = 18.4 ns) due to the decreasing of the *π–π* stacked structure; (iii) after fuming and heating of the ground sample, the longer fluorescence lifetimes (*τ* = 29.3 and 30.6 ns) and yellow-green luminous color were recovered due to the recovering of the *π–π* stacked structure (crystallization was observed in the PXRD analysis).

Furthermore, the IR spectra of the ground-heated and ground-fumed samples showed a recovery of the wavenumber of the CN stretching bands from the ground state in derivatives **1f** and **2** (a shorter wavenumber shift from the ground sample was observed; Figure 6, Figures S10 and S11), and only the CN-substituted 1,4-diphenylanthracene derivatives showed a reversible emission color change, indicating that the hydrogen bond between the Ar–CN group and the Ar–H hydrogen atom (Ar–CN⋯H–Ar) is important for the controllable reversible mechanofluorochromism in the CN-substituted 1,4-diphenylanthracene derivatives.

**Scheme 2:**
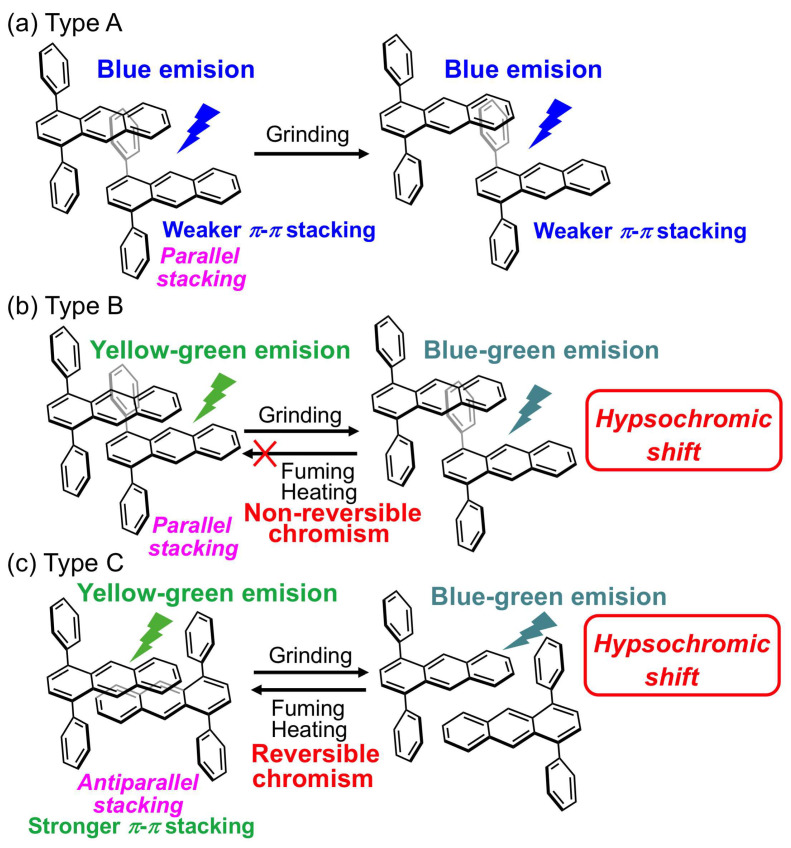
Schematic diagram of different types of mechanofluorochromic properties of 1,4-diphenylanthracene derivatives: (**a**) Type-A, non-mechanofluorochromic phenomenon for derivatives **1a**–**1c**; (**b**) Type-B shows mechanofluorochromic phenomenon but non-reversible change by fuming and heating for derivatives **1d**, **1e,** and **4**; (**c**) Type-C shows mechanofluorochromic phenomenon repeatedly changing by fuming and heating for the CN-substituted derivatives **1f**, **2,** and **3**.

Although it is difficult to further understand the critical mechanism of this mechanofluorochromism, it can be classified as follows: (Type-A) the 1,4-diphenylanthracene derivatives **1a**–**1c** show a blue luminous color in the pristine state, and the crystal packing showed a parallel-type orientation. Only a weaker *π–π* stacking was observed, and its blue luminous color probably comes from monomer emission. After grinding the pristine sample, it does not change the original luminous color (Scheme 2a); (Type-B) the derivatives **1d**, **1e,** and **4** show green and yellow-green luminous colors in the pristine state, and the crystal packing of **1d** showed a parallel-type orientation. Only weak *π–π* stacking was observed, and its green and yellow-green luminous color probably comes from the partial excimer emission or charge-transfer interactions. After grinding the pristine sample, it changes its original luminous color to a bluish color, but its original luminous color does not recover by heating and fuming of the ground samples (Scheme 2b); (Type-C) the derivatives **1f**, **2,** and **3**, which having CN-groups, show a yellow-green luminous color in the pristine state, and the crystal packing showed an antiparallel-type orientation. A stronger *π–π* stacking was observed, and its yellow-green luminous color probably comes from the excimer emission. After grinding the pristine sample, its original luminous color changes to the shorter wavelength side (yellow-green to green or yellow-green to blue-green), and its original luminous color was repeatedly recovered by heating and fuming of the ground samples. Thus, only some of the CN-substituted 1,4-diphenylanthracene derivatives exhibited a repetitive reversible mechanofluorochromism with a “*hypsochromic*” luminous color change upon grinding, heating, and fuming with solvent vapors, which may be due to the stronger *π–π* stacking attributed to the antiparallel crystal packing structure (Scheme 2c). The mechanofluorochromic properties of other phenylanthracence derivatives, such as 1,5-diphenylanthracence and fused phenylanthracene derivatives [111] are currently under investigation.

## 3. Materials, Equipment and Methods

### 3.1. General Methods

The ^1^H and ^13^C NMR spectra were recorded using Bruker (Billerica, MA, USA) Avance III (^1^H: 500 and ^13^C: 125 MHz) spectrometers. The FAB-mass spectra were recorded by a JEOL (Tokyo, Japan) JMS-700 mass spectrometer. The absorption spectra (solid and solution samples) were recorded by a Jasco (Tokyo, Japan) V-750 UV-Visible spectrometer. The fluorescence spectra for the solution samples were recorded by a Jasco (Tokyo, Japan) FP-8300 spectrometer, and the fluorescence spectra were calibrated using rhodamine B as the reference. The fluorescence spectra for the solid samples were recorded by a Flame-S, Ocean Optics (Orlando, FL, USA) fiber-optic spectrometer (*λ*_ex_ = 365 nm). The absolute PL quantum yields (*Φ*) were determined by a Quantaurus-QY (Hamamatsu Photonics, Hamamatsu, Japan) instrument. The emission lifetimes were measured by a Quantaurus-Tau (Hamamatsu Photonics) instrument. The DSC thermograms of the solid samples were recorded by a TA Instruments (New Castle, Delaware, USA) DSC 2920 modulated DSC and were recorded at the constant heating rate of 10 °C/min. The infrared spectra were recorded using a Jasco (Tokyo, Japan) FTIR 4600 spectrometer equipped with an attenuated total reflection (ATR) unit. All the solvents and reagents were purified according to standard procedures. 3,5-dicyanophenylboronic acid [65] and 1,4-dibromoanthracene [112,113,114] were prepared according to published procedure.

### 3.2. Synthesis of 1,4-Diphenylanthracene Derivatives ***1***–***4***

General Procedure: 1,4-dibromoanthracene (103 mg, 0.307 mmol), phenylboronic acid (90.6 mg, 0.743 mmol), Pd(PPh_3_)_4_ (35.6 mg, 30.8 μmol) were mixed in 20 mL of DME. Then a 1 M K_2_CO_3_ aqueous solution (3.0 mL, 3.00 mmol) was added. The obtained mixture was degassed 3 times by a freeze-pump-through method, and then the mixture was heated at 85 °C for 18 h under an Ar atmosphere. After removing the solvent in vacuo and adding water (80 mL), the mixture was extracted with CH_2_Cl_2_ (80 mL), dried over anhydrous Na_2_SO_4,_ and the solvent evaporated. The 1,4-diphenylanthracene **1a** was isolated (98.2 mg, 97%) by column chromatography (SiO_2_, CH_2_Cl_2,_ or CH_2_Cl_2_/hexane) followed by recrystallization from MeOH or CH_2_Cl_2_/MeOH.

**1a** (R = H): yield 87%, pale-yellow needles, mp: 157.8–158.8 °C; ^1^H NMR (500 MHz, CDCl_3_): *δ* (ppm) = 8.52 (s, 2H), 7.87 (dd, *J* = 6.4, 3.3 Hz, 2H), 7.63 (d, *J* = 8.2 Hz, 4H), 7.56 (t, *J* = 7.5 Hz, 4H), 7.49 (t, *J* = 7.3 Hz, 2H), 7.44 (s, 2H), 7.40 (dd, *J* = 6.6, 3.2 Hz, 2H), ^13^C NMR (125 MHz, CDCl_3_): *δ* (ppm) = 141.2 (Cq), 140.0 (Cq), 131.5 (Cq), 130.8 (Cq), 130.4 (CH), 128.6 (CH), 128.5 (CH), 127.6 (CH), 125.9 (CH), 125.7 (CH), 125.5 (CH), HRMS (FAB, NBA) *m*/*z* = 330.1417 (calculated for M^+·^: 330.1409), Anal. Calcd. for C_26_H_18_: C: 94.51, H: 5.49, Found: C: 94.41, H: 5.58.
**1b** (R = Me): yield 76%, pale-yellow prisms, mp: 192.3–193.3 °C; ^1^H NMR (500 MHz, CDCl_3_): *δ* (ppm) = 8.54 (s, 2H), 7.87 (dd, *J* = 6.4, 3.3 Hz, 2H), 7.52 (d, *J* = 8.0 Hz, 4H), 7.42 (s, 2H), 7.39 (dd, *J* = 6.6, 3.2 Hz, 2H), 7.37 (d, *J* = 7.7 Hz, 4H), 2.50 (s, 6H), ^13^C NMR (125 MHz, CDCl_3_): *δ* (ppm) = 139.6 (C_q_), 138.1 (C_q_), 137.1 (C_q_), 131.3 (C_q_), 130.7 (C_q_), 130.1 (CH), 129.1 (CH), 128.3 (CH), 125.8 (CH), 125.4 (CH), 125.3 (CH), 21.3 (CH_3_), HRMS (FAB, NBA) *m*/*z* = 358.1723 (calculated for M^+·^: 358.1722), Anal. Calcd. for C_28_H_22_·0.4H_2_O: C: 91.97, H: 6.28, Found: C: 92.02, H: 6.17.
**1c** (R = F): yield 94%, pale-yellow prisms, mp: 160.0–161.0 °C; ^1^H NMR (500 MHz, CDCl_3_): *δ* (ppm) = 8.43 (s, 2H), 7.87 (dd, *J* = 6.4, 3.3 Hz, 2H), 7.55 (m, 4H), 7.42 (dd, *J* = 6.6, 3.2 Hz, 2H), 7.38 (s, 2H), 7.24 (m, 4H), ^13^C NMR (125 MHz, CDCl_3_): *δ* (ppm) = 162.6 (C_q_, d, ^1^*J*_C-F_ = 246.7 Hz), 139.1 (C_q_), 136.9 (C_q_, d, ^4^*J*_C-F_ = 3.0 Hz), 131.9 (CH), 131.8 (CH), 131.6 (C_q_), 130.8 (C_q_), 128.4 (CH), 126.0 (CH), 125.3 (CH), 115.6 (CH, d, ^2^*J*_C-F_ = 21.4 Hz), ^19^F NMR (470 MHz, CDCl_3_): *δ* (ppm) = −116.1, HRMS (FAB, NBA) *m*/*z* = 366.1230 (calculated for M^+·^: 366.1220), Anal. Calcd. for C_26_H_16_F_2_: C: 85.23, H: 4.40, Found: C: 85.15, H: 4.46.
**1d** (R = OMe): yield 80%, yellow solids, mp: 246.9–247.9 °C; ^1^H NMR (500 MHz, CDCl_3_): *δ* (ppm) = 8.53 (s, 2H), 7.88 (dd, *J* = 6.4, 3.3 Hz, 2H), 7.54 (d, *J* = 8.7 Hz, 4H), 7.404 (s, 2H), 7.401 (dd, *J* = 6.4, 3.3 Hz, 2H), 7.10 (d, *J* = 8.7 Hz, 4H), 3.93 (s, 6H), ^13^C NMR (125 MHz, CDCl_3_): *δ* (ppm) = 159.2 (C_q_), 139.4 (C_q_), 133.6 (C_q_), 131.5 (C_q_), 131.4 (CH), 131.1 (C_q_), 128.5 (CH), 125.9 (CH), 125.6 (CH), 125.5 (CH), 114.0 (CH), 55.5 (CH_3_), HRMS (FAB, NBA) *m*/*z* = 390.1619 (calculated for M^+·^: 390.1620), Anal. Calcd. for C_28_H_24_O_3_·H_2_O: C: 82.33, H: 5.92, Found: C: 82.30, H: 5.57.
**1e** (R = CF_3_): yield 88%, yellow solids, mp: 207.5–208.5 °C; ^1^H NMR (500 MHz, CDCl_3_): *δ* (ppm) = 8.42 (s, 2H), 7.89 (dd, *J* = 6.4, 3.2 Hz, 2H), 7.83 (d, *J* = 8.0 Hz, 4H), 7.73 (d, *J* = 8.0 Hz, 4H), 7.45 (dd, *J* = 6.6, 3.1 Hz, 2H), 7.43 (s, 2H), ^13^C NMR (125 MHz, CDCl_3_): *δ* (ppm) = 144.5 (C_q_), 139.1 (C_q_), 131.6 (C_q_), 130.5 (CH), 130.1 (C_q_), 129.83 (q, ^2^*J*_C-F_ = 32.5 Hz), 128.3 (CH), 126.1 (CH), 125.8 (CH), 125.5 (CH, q, ^3^*J*_C-F_ = 3.6 Hz), 125.1 (CH), 124.3 (CF_3_, q, ^1^*J*_C-F_ = 272.1 Hz), ^19^F NMR (470 MHz, CDCl_3_): *δ* (ppm) = −63.5, HRMS (FAB, NBA) *m*/*z* = 466.1154 (calculated for M^+·^: 466.1156), Anal. Calcd. for C_28_H_16_F_6_·0.2H_2_O: C: 71.55, H: 3.52, Found: C: 71.41, H: 3.50.
**1f** (R = CN): yield 88%, yellow solids, mp: 278.5–279.5 °C; ^1^H NMR (500 MHz, CDCl_3_): *δ* (ppm) = 8.39 (s, 2H), 7.90 (dd, 2H), 7.89 (d, *J* = 8.2 Hz, 4H), 7.75 (d, *J* = 8.2 Hz, 4H), 7.49 (dd, *J* = 6.6, 3.2 Hz, 2H), 7.45 (s, 2H), ^13^C NMR (125 MHz, CDCl_3_): *δ* (ppm) = 145.5 (C_q_), 139.0 (C_q_), 132.4 (CH), 131.7 (C_q_), 130.8 (CH), 129.7 (C_q_), 128.2 (CH), 126.4 (CH), 125.8 (CH), 125.0 (CH), 118.8 (C_q_), 111.6 (C_q_), HRMS (FAB, NBA) *m*/*z* = 380.1324 (calculated for M^+·^: 380.1313), Anal. Calcd. for C_28_H_16_N_2_·0.2H_2_O: C: 87.57, H: 4.30, N: 7.29, Found: C: 87.51, H: 4.37, N: 7.17.
**2** (3-CN): yield 88%, yellow solids, mp: 208.1–209.1 °C; ^1^H NMR (500 MHz, CDCl_3_): *δ* (ppm) = 8.35 (s, 2H), 7.92 (t, *J* = 1.6 Hz, 2H), 7.92 (dd, 2H), 7.87 (dt, *J* = 7.7, 1.5 Hz, 2H), 7.83 (dt, *J* = 7.8, 1.4 Hz, 2H), 7.50 (dd, *J* = 6.6, 3.2 Hz, 2H), 7.43 (s, 2H), ^13^C NMR (125 MHz, CDCl_3_): *δ* (ppm) = 141.9 (C_q_), 138.4 (C_q_), 134.5 (CH), 133.5 (CH), 131.7 (C_q_), 131.3 (CH), 129.9 (C_q_), 129.4 (CH), 128.2 (CH), 126.4 (CH), 125.9 (CH), 124.9 (CH), 118.7 (C_q_), 112.9 (C_q_), HRMS (FAB, NBA) *m*/*z* = 380.1306 (calculated for M^+·^: 380.1313), Anal. Calcd. for C_28_H_16_N_2_·0.4H_2_O: C: 86.75, H: 4.37, N: 7.23, Found: C: 86.65, H: 4.29, N: 7.12.
**3** (3,5-CN_2_, R′=CN): yield 45%, yellow solids, mp: >300 °C; ^1^H NMR (500 MHz, CDCl_3_): *δ* (ppm) = 8.24 (s, 2H), 8.13 (d, *J* = 1.5 Hz, 4H), 8.11 (t, *J* = 1.5 Hz, 2H), 7.95 (dd, *J* = 6.5, 3.3 Hz, 2H), 7.58 (dd, *J* = 6.6, 3.1 Hz, 2H), 7.44 (s, 2H), ^13^C NMR (125 MHz, CDCl_3_): *δ* (ppm) = 143.3 (C_q_), 137.2 (CH), 137.0 (C_q_), 134.5 (CH), 132.1 (C_q_), 129.2 (C_q_), 128.2 (CH), 127.2 (CH), 126.2 (CH), 124.6 (CH), 116.5 (C_q_), 114.7 (C_q_), HRMS (FAB, NBA) *m*/*z* = 430.1214 (calculated for M^+·^: 430.1218).
**4** (3,5-F_2_, R′ = F): yield 99%, yellow solids, mp: 196.8–197.8 °C; ^1^H NMR (500 MHz, CDCl_3_): *δ* (ppm) = 8.45 (s, 2H), 7.92 (dd, *J* = 6.4, 3.3 Hz, 2H), 7.47 (dd, *J* = 6.6, 3.1 Hz, 2H), 7.40 (s, 2H), 7.13 (dd, ^3^*J*_F-H_ = 7.8 Hz, ^4^*J*_H-H_ = 2.0 Hz, 4H), 6.96 (tt, ^3^*J*_F-H_ = 9.0 Hz, ^4^*J*_H-H_ = 2.3 Hz, 2H), ^13^C NMR (125 MHz, CDCl_3_): *δ* (ppm) = 163.2 (C_q_, dd, ^1^*J*_C-F_ = 249.1, ^3^*J*_C-F_ = 12.9 Hz), 144.1 (C_q_, t, ^3^*J*_C-F_ = 9.5 Hz), 138.6 (C_q_), 131.9 (C_q_), 130.0 (C_q_), 128.5 (CH), 126.4 (CH), 125.7 (CH), 125.1 (CH), 113.3 (CH, dd, ^2^*J*_C-F_ = 19.3, ^4^*J*_C-F_ = 6.1 Hz), 103.3 (CH, t, ^2^*J*_C-F_ = 25.3 Hz), ^19^F NMR (470 MHz, CDCl_3_): *δ* (ppm) = −110.8, HRMS (FAB, NBA) *m*/*z* = 402.1045 (calculated for M^+·^: 402.1032), Anal. Calcd. for C_26_H_14_F_4_·0.5H_2_O: C: 75.91, H: 3.68, Found: C:76.01, H: 3.75.

### 3.3. Computational Methods

Density functional theory (DFT) with the B3LYP functional at the 6–31G* basis set level was used to calculate the electro cloud distribution of the HOMO and LUMO in a vacuum. For the calculation from the obtained single crystal structures, two molecules, which were stacked on each other, were located, and the non-hydrogen atom coordinates were fixed. Only the hydrogen atoms were optimized to obtain the conformation for the DFT calculation. All of the calculations were performed using Gaussian 16W C.01 [115].

### 3.4. Crystallographic Analysis

Crystals suitable for X-ray crystallography were grown from the solutions of **1a** (R = H), **1c** (R = F) in hexane solution that were stored in a test tube and left to stand for several days. For compounds **1d** (R = OMe) and **1f** (R = CN), the crystals were obtained from DME solution. For compounds **1b** (R = Me) and **2** (3-CN), the crystals were obtained from CH_2_Cl_2_ and CHCl_3_ solutions, respectively. For the crystallographic data collection of **1a** (R = H) and **2** (3-CN), Rigaku XtaLAB mini with graphite-monochromated Mo-Kα radiation was used. Calculations were performed using the Olex2, version 1.3.0 crystallographic software packages [116]. The crystal structures were solved by a direct method using Shelxt Version 2014/2015 [117,118]. The structure refinements were performed by a full-matrix least squares method using Shelxl Version 2014/2015 [119,120]. For the crystallographic data collection of **1b** (R = Me), **1c** (R = F), **1d** (R = OMe), and **1f** (R = CN), a Bruker D8 VENTURE equipped with a rotating anode of Mo-Kα or Cu-Kα radiation and a PHOTON III detector was used. Calculations were performed using the Bruker APEX3, 2019 [121] software package. Details of the data are summarized in Tables S3 and S4. All non-hydrogen atoms were anisotropically refined. All the hydrogen atoms were placed in idealized positions and were included in the structure factor calculations but were not refined. CCDC-2314555-2314560 contains the supplementary crystallographic data for this paper. These data can be obtained free of charge from The Cambridge Crystallographic Data Centre.

### 3.5. PXRD Analysis

Powder X-ray diffraction (PXRD) patterns of the samples were recorded using a Rigaku Smartlab X-ray diffractometer with CuK_α_ radiation (1.5406 Å) at a voltage of 40 kV and a current of 30 mA, and the data were collected with a step size of 0.01° over a 2*θ* range of 5–50°.

## 4. Conclusions

In this study, several types of 1,4-diphenylanthracene derivatives **1**–**4** were prepared, and their photophysical properties were observed in the solid and solution states. The CN-group substituted 1,4-diphenylanthracene derivative **2** exhibited a relatively high fluorescence quantum yield (*Φ* = 0.71) in the solid state than that in the solution state. Interestingly, for some derivatives, an increase in the fluorescence quantum yield was observed in the PMMA film (1 wt%) over both the solid state and the solution state. More interestingly, some of the 1,4-diphenylanthracene derivatives exhibited mechanofluorochromic properties with “*hypsochromic shift*” of the luminous color depending on the substituents of the phenyl group. Especially for some CN-substituted derivatives, a reversible luminous color change with a “*hypsochromic shift*” was observed, probably due to the formation of the antiparallel-type packing structure. These “*hypsochromic*” anthracene derivatives could probably be applied as new mechanofluorochromic materials.

## Figures and Tables

**Table 1:** Fluorescence properties of 1,4-diphenylanthracene derivatives.

Compounds	*λ*_max_/nm ^a^, Luminous Color ^b^			*Φ*_f_ ^c^		*τ*^d^/ns	
	Pristine	Ground	Solution ^e^	Pristine (Ground)	Solution ^f^	Pristine (Ground)	Solution ^f^
1a (R = H)	455, 480, LB	448, 481, LB	422, 438, B	0.20 (0.23)	0.47	2.27 (6.48)	3.02
1b (R = Me)	455, 480, LB	450, 483, LB	432, 443, B	0.31 (0.21)	0.49	3.96 (13.4)	3.08
1c (R = F)	452, 480, LB	448, 482, LB	421, 437, B	0.24 (0.25)	0.49	3.05 (13.7)	3.36
1d (R = OMe)	515, YG	486, BG	454, LB	0.15 (0.18)	0.40	12.4 (10.4)	2.69
1e (R = CF_3_)	497, 530, G	488, BG	438, 440, B	0.37 (0.33)	0.51	4.69 (7.63)	2.76
1f (R = CN)	514, YG	497, G	458, LB	0.26 (0.33)	0.55	32.8 (18.4)	2.57
2 (3-CN)	522, YG	493, BG	428, 439, B	0.71 (0.37)	0.46	109 (91.5)	2.85
3 (R′ = CN)	518, YG	497, G	455, LB	0.15 (0.05)	0.41	7.39 (4.09)	2.57
4 (R′ = F)	489, 536, G	489, BG	426, 438, B	0.44 (0.22)	0.50	5.22 (5.36)	2.90

^a^ Solution and solid-state emission (*λ*_ex_ = 365 nm). ^b^ Luminous color, B: blue, G: green, BG: blue-green, LB: light-blue, YG: yellow-green under 365 nm UV irradiation. ^c^ Fluorescence quantum yield determined by a calibrated integrating sphere at *λ*_ex_ = 365 nm. ^d^ Averaged fluorescence lifetime at *λ*_ex_ = 365 nm. ^e^ CHCl_3_ solution (25 μM, *λ*_ex_ = 365 nm). ^f^ CHCl_3_ solution (10 μM) after Ar bubbling.

## Data Availability

Data are contained within the article and supplementary materials.

## Supplementary Materials

The following supporting information can be downloaded at: https://www.mdpi.com/article/10.3390/molecules29020407/s1. Fluorescence spectra of the pristine, ground, and stored samples of 1,4-diphenylanthracene derivatives (Figure S1), fluorescence images of the pristine, ground, stored, heated and fumed sample of 1,4-diphenylanthracene derivatives (Figures S2 and S3), normalized fluorescence spectra of the pristine, ground, ground-fumed and ground-heated samples (Figure S4), vapochromic properties of 1,4-diphenylanthracene derivatives (Figures S5 and S6),thermochromic properties of 1,4-diphenylanthracene derivatives (Figures S7–S9), IR spectra of the CN-substituted 1,4-diphenylanthracene derivatives (Figures S10 and S11), DSC profiles of 1,4-diphenylanthracene derivatives (Figure S12), UV-vis absorption spectra of the pristine and ground samples (Figure S13), UV-vis absorption spectra of 1,4-diphenylanthracene derivatives in solution (Figure S14), fluorescence spectra of 1,4-diphenylanthracene derivatives in solution (Figures S15–S17), fluorescence spectra of 1,4-diphenylanthracene derivatives in PMMA film (Figures S18 and S19), fluorescence lifetime decay profiles of pristine, ground, ground-heated and ground-fumed samples (Figure S20), fluorescence lifetime decay profiles of 1,4-diphenylanthracene derivatives in solution (Figure S21), fluorescence lifetime decay profiles of 1,4-diphenylanthracene derivatives in PMMA film (Figure S22), photophysical properties of 1,4-diphenylanthracene derivatives (Tables S1 and S2), single crystal X-ray analysis of 1,4-diphenylanthracene derivatives (Tables S3 and S4, Figures S23–S37), Powder X-ray diffraction (PXRD) patterns (Figure S38), DFT calculation of the obtained crystal structures (Figures S39–S42), UV irradiation experiments for 1,4-diphenylanthracene derivatives (Figures S43 and S44), andNMR spectra of new compounds.
