# Supplementary material for: Mechanofluorochromic Properties of 1,4-Diphenylanthracene Derivatives with Hypsochromic Shift
Source: Molecules. 2024 Jan 14;29(2):407. doi: 10.3390/molecules29020407 (PMC10820785; doi:10.3390/molecules29020407)

Electronic Supplementary Information (ESI) for Molecules

Mechanofluorochromic properties of 1,4-diphenylanthracene derivatives with hypsochromic shift

Fumihiro Kannen[1], Tadatoshi Adachi[1], Manato Nishimura[1], Kenji Yoza[2]

and Takahiro Kusukawa[1]*

[1]Faculty of Molecular Chemistry and Engineering,

Graduate School of Science and Technology, Kyoto Institute of Technology,

Matsugasaki, Sakyo-ku Kyoto 606-8585, Japan

[2]Bruker Japan K.K., 3-9 Moriya-cho, Kanagawa-ku, Yokohama 221-0022, Japan

Outline

1. Supplementary figures and tables p3

1.1 Fluorescence spectra of the pristine, ground, and stored samples of 1,4-diphenylanthracene derivatives p3

1.2 Fluorescence images of the pristine, ground, stored, heated and fumed sample of 1,4-diphenylanthracene derivatives p5

1.3 Fluorescence spectra of the pristine, ground, ground-fumed, and ground-heated samples of 1,4-diphenylanthracene derivatives p11

1.4 Vapochromic properties of 1,4-diphenylanthracene derivatives p13

1.5 Thermochromic properties of 1,4-diphenylanthracene derivatives p14

1.6 IR spectra of the CN-substituted 1,4-diphenylanthracene derivatives p16

1.7 DSC profiles of 1,4-diphenylanthracene derivatives p17

1.8 UV-vis absorption spectra of the pristine and ground samples p18

1.9 UV-vis absorption spectra of 1,4-diphenylanthracene derivatives in $CHCl_3$ solution p20

1.10 Fluorescence spectra of 1,4-diphenylanthracene derivatives in $CHCl_3$ solution p22

1.11 Fluorescence spectra of 1,4-diphenylanthracene derivatives in PMMA film p25

1.12 Fluorescence lifetime decay profiles of pristine, ground, ground-heated and ground-fumed samples p27

1.13 Fluorescence lifetime decay profiles of 1,4-diphenylanthracene derivatives in $CHCl_3$ solution p30
1.14 Fluorescence lifetime decay profiles of 1,4-diphenylanthracene derivatives in PMMA film p31
1.15 Photophysical properties of 1,4-diphenylanthracene derivatives p32
2. X-ray analysis of 1,4-diphenylanthracene derivatives p33
2.1 Single crystal X-ray analysis of 1,4-diphenylanthracene derivatives p33
2.2 Powder X-ray diffraction (PXRD) patterns p41
3. DFT calculation of the obtained crystal structures p43
4. UV irradiation experiments for 1,4-diphenylanthracene derivatives p46
5. NMR spectra of new compounds p47

1. Supplementary figures and tables

1.1 Fluorescence spectra of the pristine, ground and stored (5 min. after grinding) samples of 1,4-diphenylanthracene derivatives

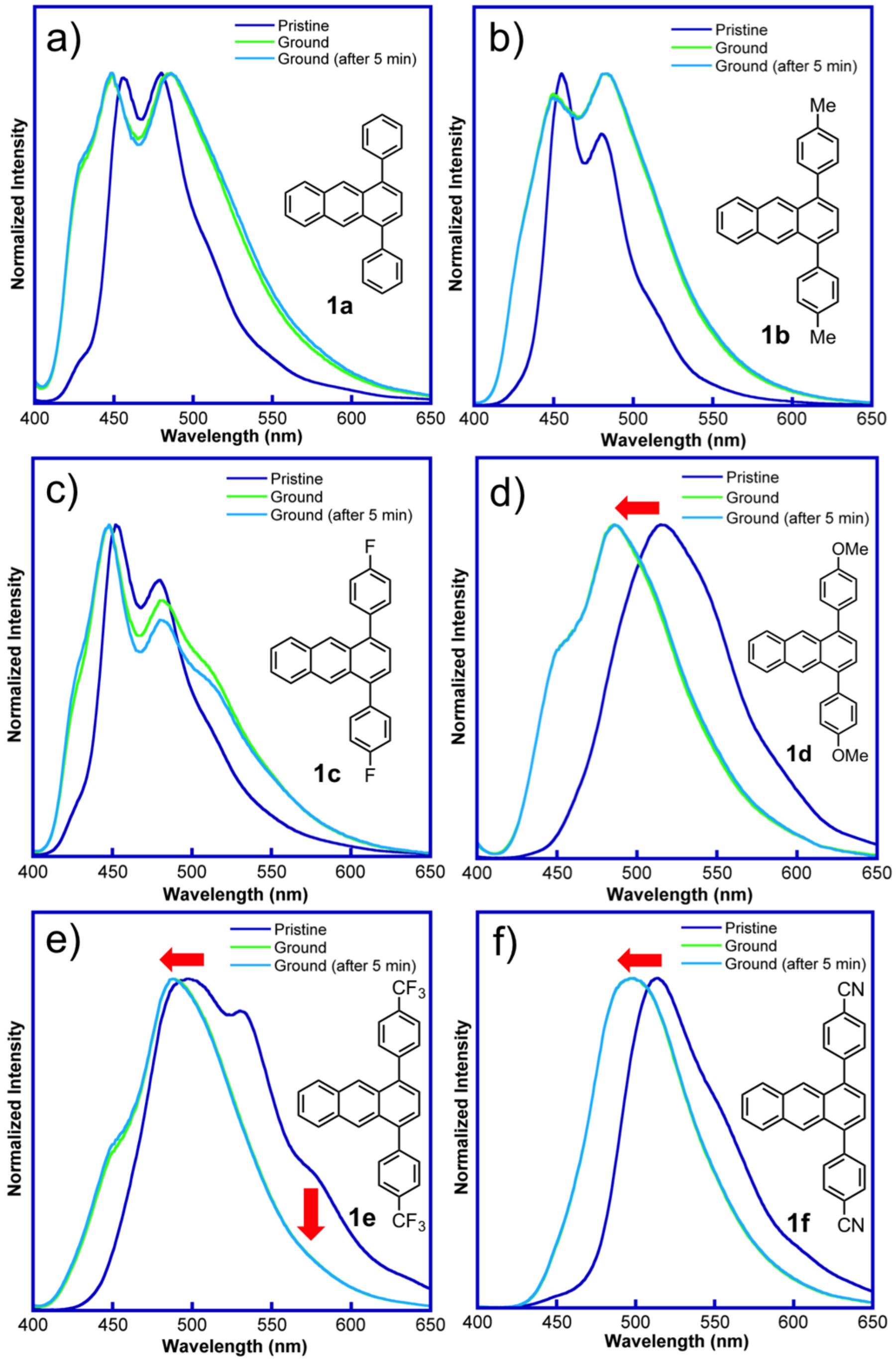

Figure S1. Normalized fluorescence spectra of pristine, ground and stored (5 min. after grinding) samples (λ_{ex} =365 nm), a) **1a** (R=H), b) **1b** (R=Me), c) **1c** (R=F), d) **1d** (R=OMe), e) **1e** (R=CF_3), f) **1f** (R=CN).

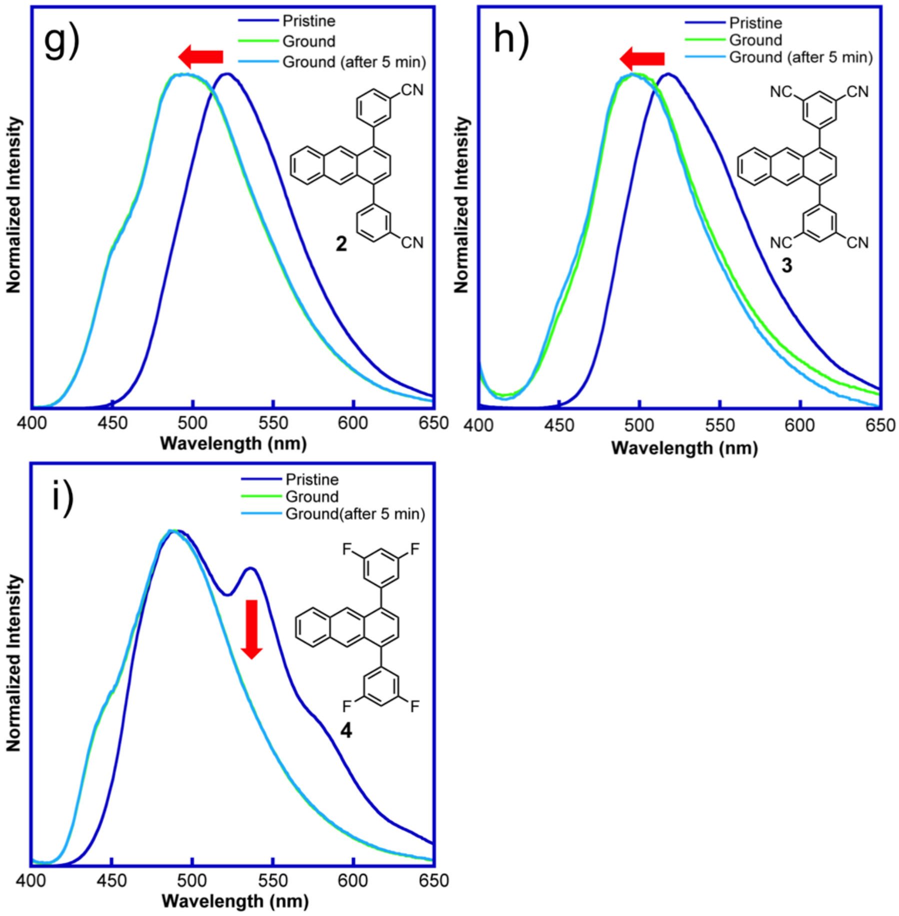

Figure S1 (contd.). Normalized fluorescence spectra of pristine, ground and stored (5 min. after grinding) samples (λ_{ex} =365 nm), g) **2** (3-CN), h) **3** (3,5-CN_2, R'=CN), i) **4** (3,5-F_2, R'=F).

1.2 Fluorescence images of the pristine, ground, stored (5 min after grinding) samples, heated and fumed samples of 1,4-diphenylanthracene derivatives

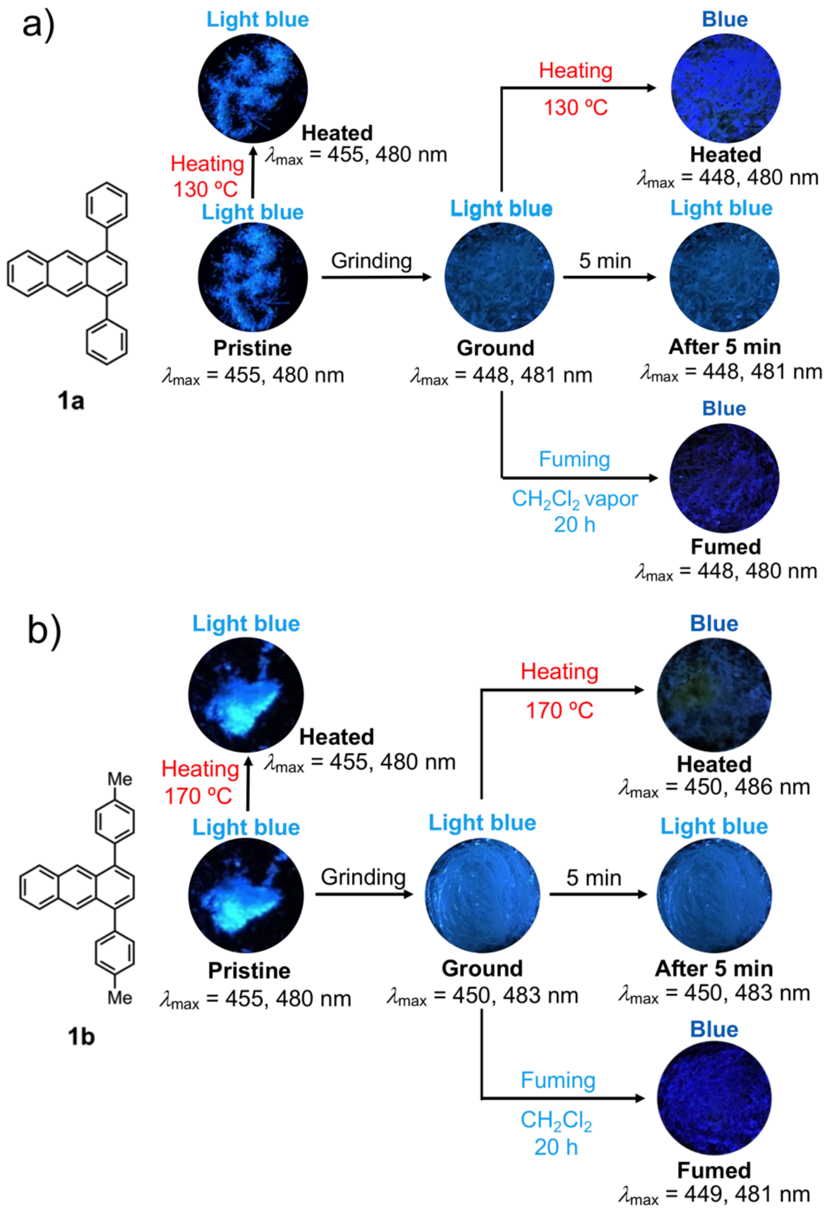

Figure S2. Fluorescence images of 1,4-diphenylanthracene derivatives pristine, ground, stored (5 min after grinding), heated and fumed samples during a 365 nm UV irradiation, a) **1a** (R=H), b) **1b** (R=Me).

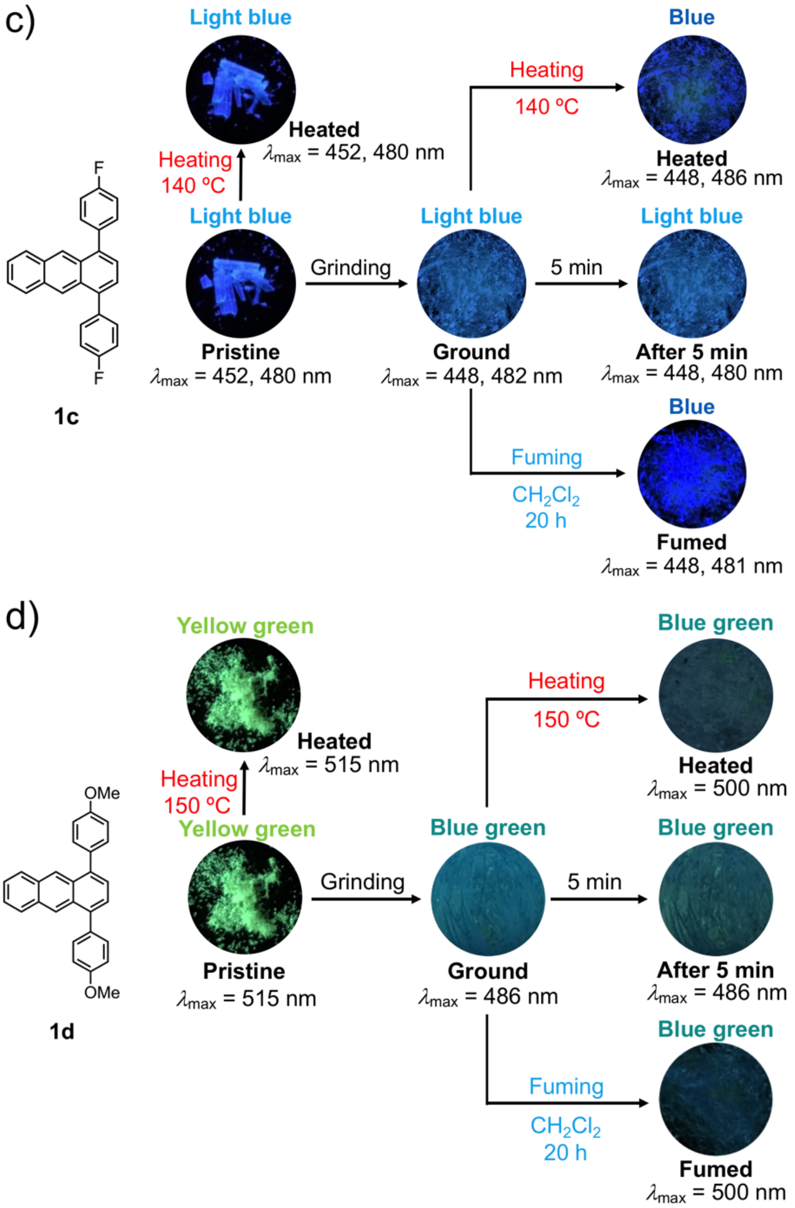

Figure S2 (contd.). Fluorescence images of 1,4-diphenylanthracene derivatives pristine, ground, stored (5 min after grinding), heated and fumed samples during a 365 nm UV irradiation, c) **1c** (R=F), b) **1d** (R=OMe).

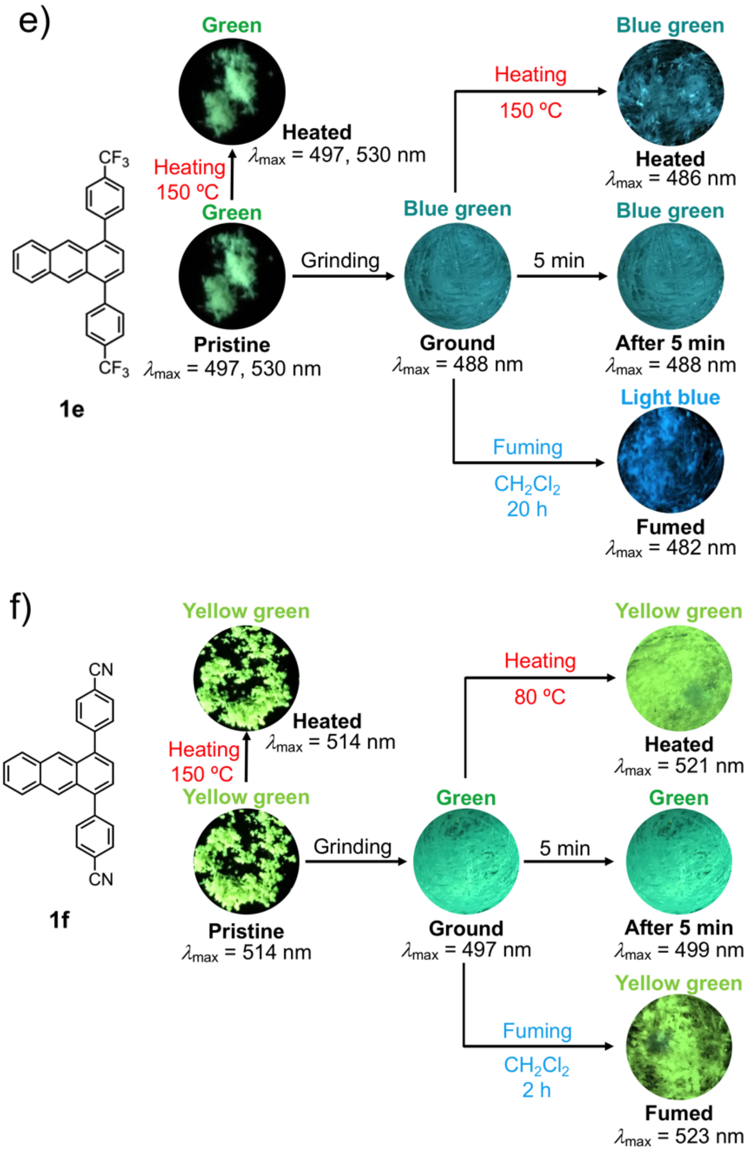

Figure S2 (contd.). Fluorescence images of 1,4-diphenylanthracene derivatives pristine, ground, stored (5 min after grinding), heated and fumed samples during a 365 nm UV irradiation, e) **1e** (R=CF_3), f) **1f** (R=CN).

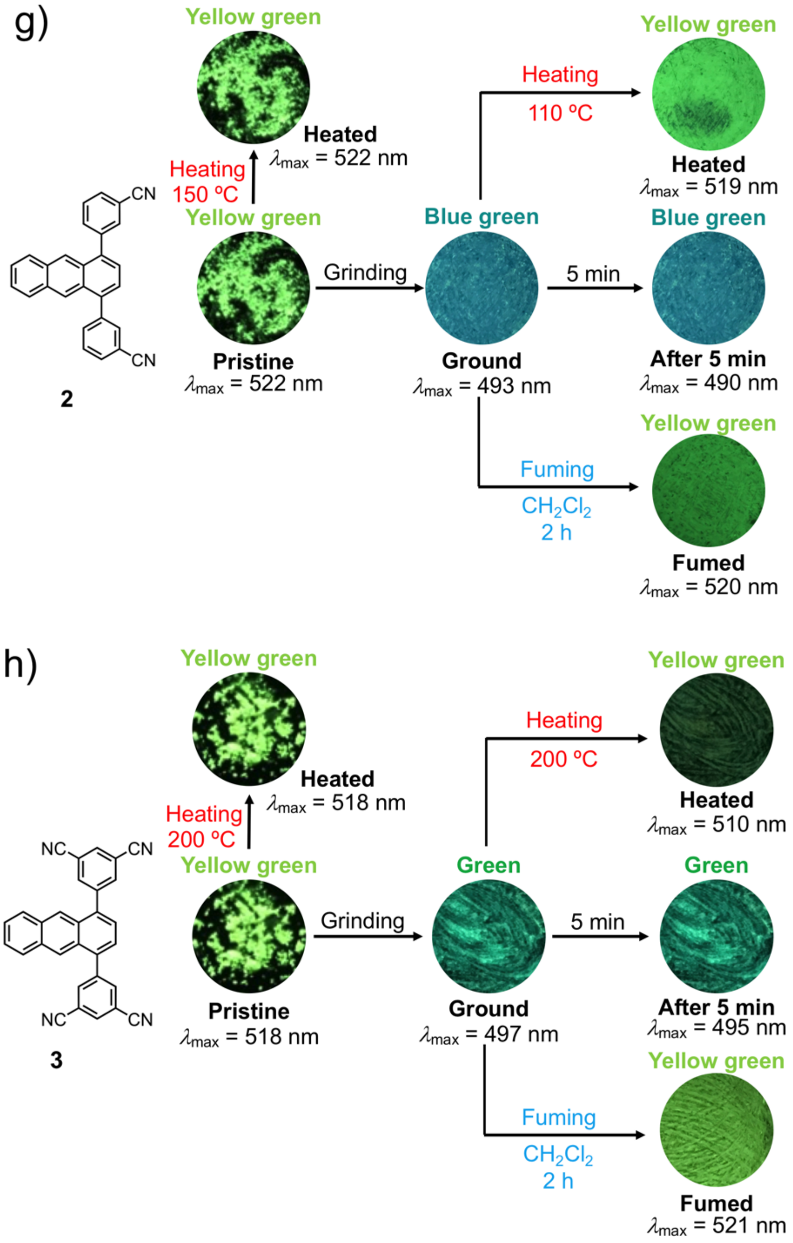

Figure S2 (contd.). Fluorescence images of 1,4-diphenylanthracene derivatives pristine, ground, stored (5 min after grinding), heated and fumed samples during a 365 nm UV irradiation, g) **2** (3-CN), h) **3** (3,5-CN$_2$, R'=CN).

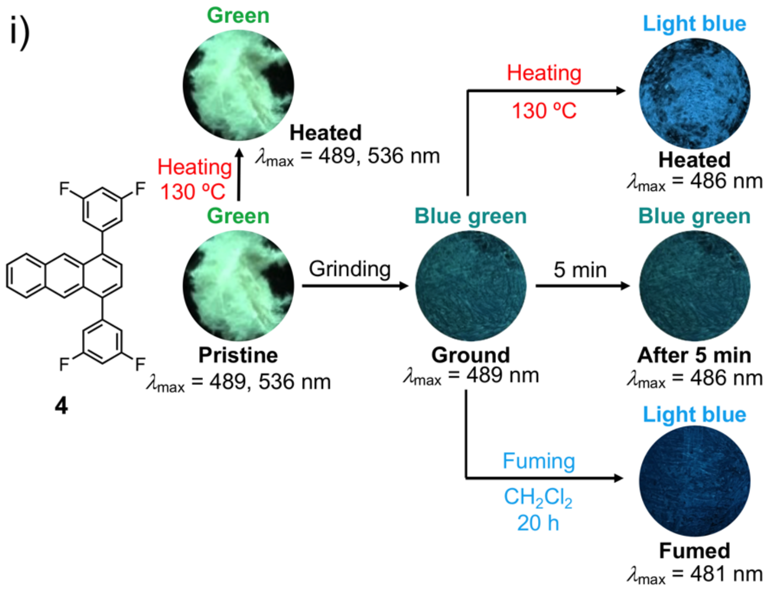

Figure S2 (contd.). Fluorescence images of 1,4-diphenylanthracene derivatives pristine, ground, stored (5 min after grinding), heated and fumed samples during a 365 nm UV irradiation, i) **4** (3,5-F_2, R'=F).

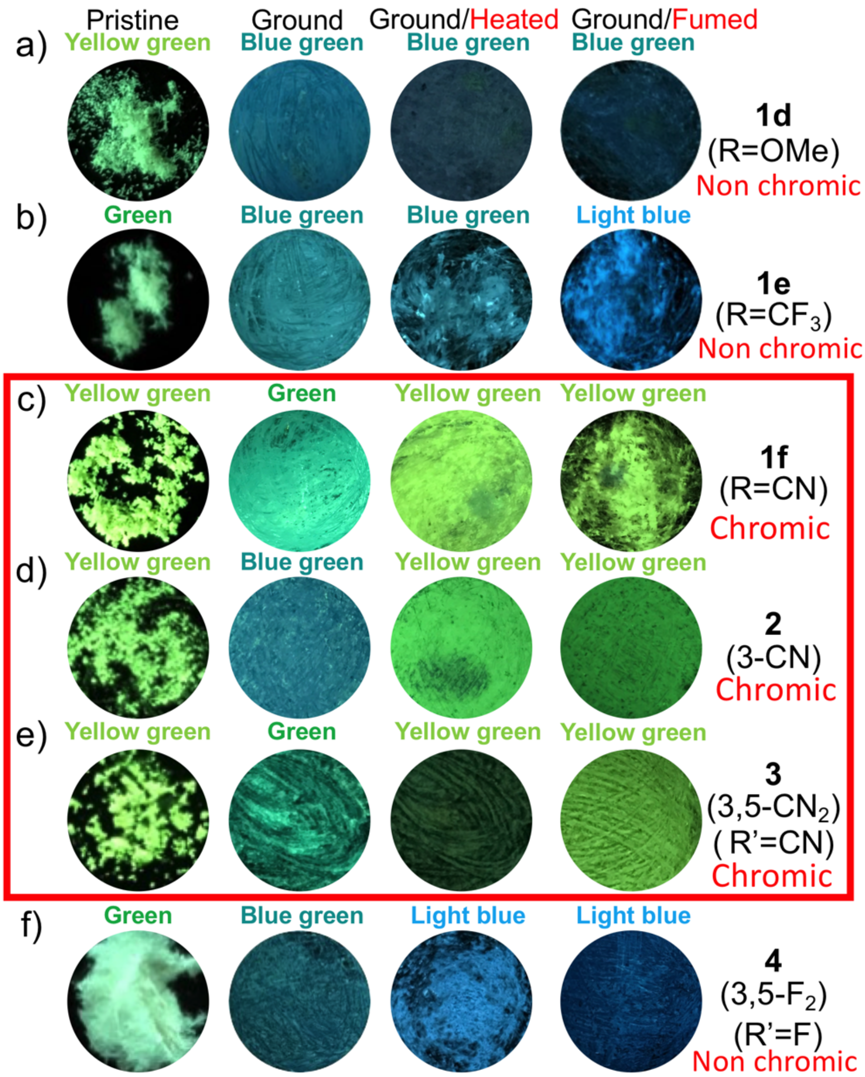

Figure S3. Fluorescent images of the pristine, ground, heated and fumed (CH_2Cl_2 vapor) samples of 1,4-diphenylanthracene derivatives **1d**-**1f**, and **2**-**4** during a 365 nm UV irradiation, a) **1d** (R=OMe, heated at 150 °C for 5 min., fumed with CH_2Cl_2 vapor for 20 h), b) **1e** (R=CF_3, heated at 150 °C for 5 min., fumed with CH_2Cl_2 vapor for 20 h), c) **1f** (R=CN, heated at 80 °C for 5 min., fumed with CH_2Cl_2 vapor for 2 h), d) **2** (3-CN, heated at 110 °C for 5 min., fumed with CH_2Cl_2 vapor for 2 h), e) **3** (3,5-CN_2, R'=CN, heated at 200 °C for 5 min., fumed with CH_2Cl_2 vapor for 2 h), f) **4** (3,5-F_2, R'=F, heated at 130 °C for 5 min., fumed with CH_2Cl_2 vapor for 20 h).

1.3 Fluorescence spectra of the pristine, ground, ground-fumed and ground-heated samples of 1,4-diphenylanthracene derivatives

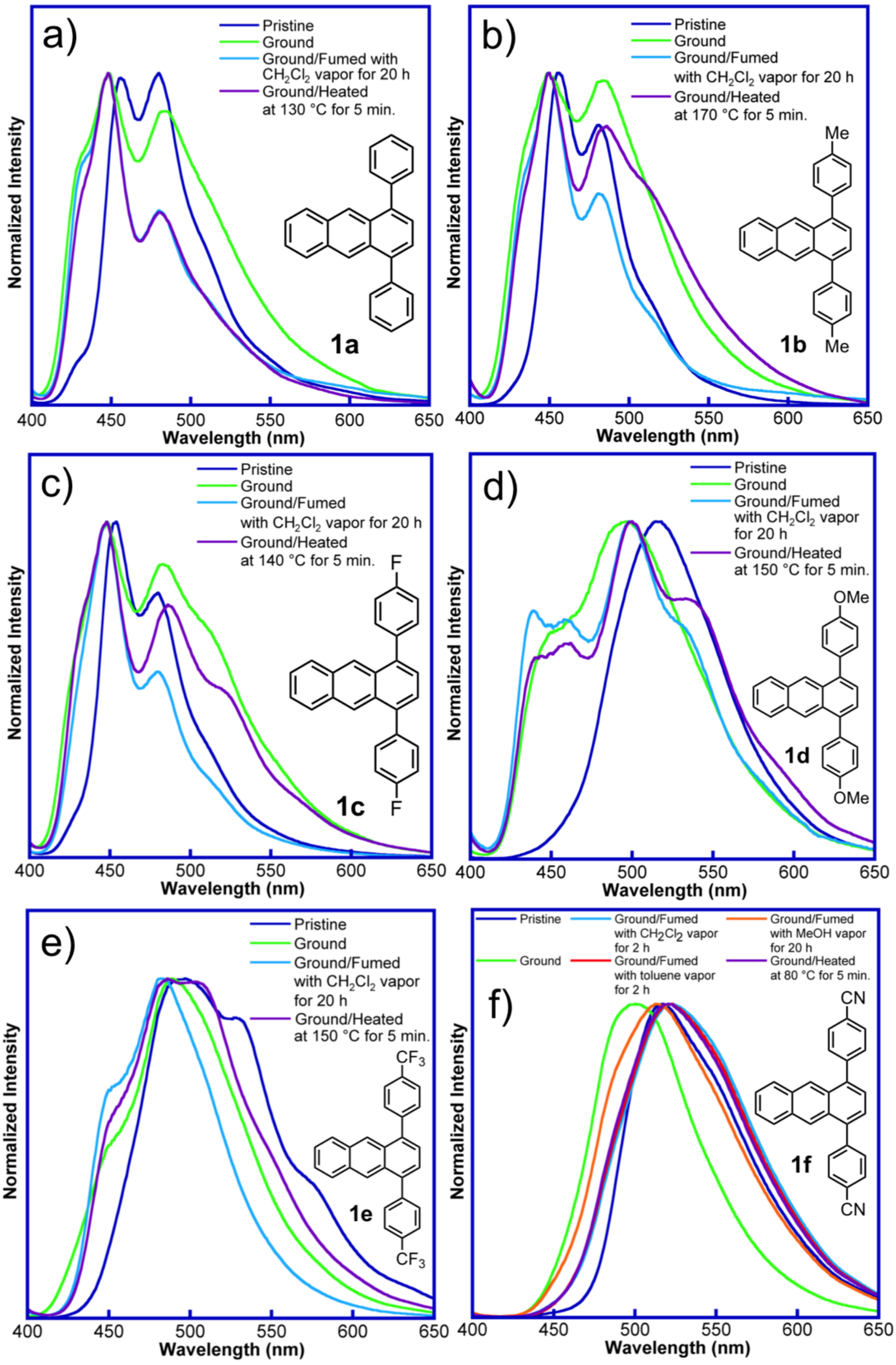

Figure S4. Normalized fluorescence spectra of the pristine, ground, ground-fumed and ground-heated samples (λ_{ex}=365 nm), a) **1a** (R=H), b) **1b** (R=Me), c) **1c** (R=F), d) **1d** (R=OMe), e) **1e** (R=CF_3), f) **1f** (R=CN).

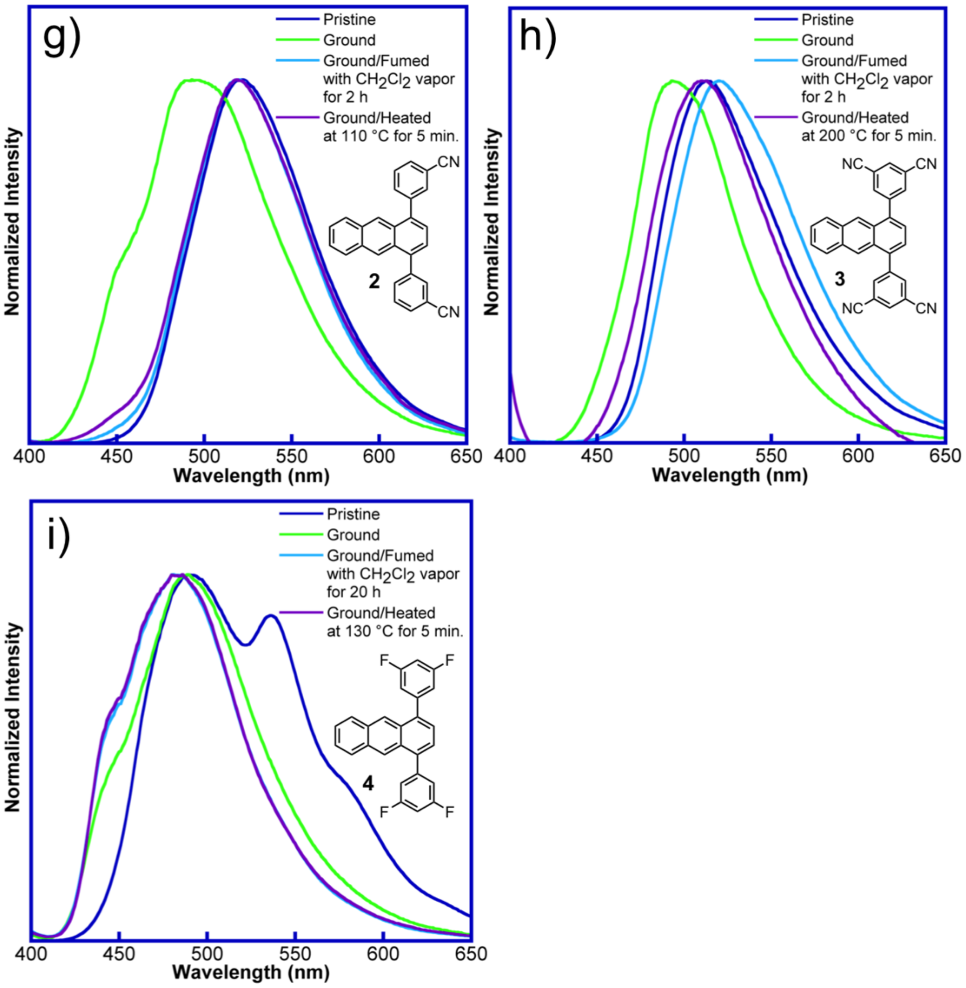

Figure S4 (contd.). Normalized fluorescence spectra of the pristine, ground, ground-fumed and ground-heated samples (λ_{ex}=365 nm), g) **2** (3-CN), h) **3** (3,5-CN_2, R'=CN), i) **4** (3,5-F_2, R'=F).

1.4 Vapochromic properties of 1,4-diphenylanthracene derivatives

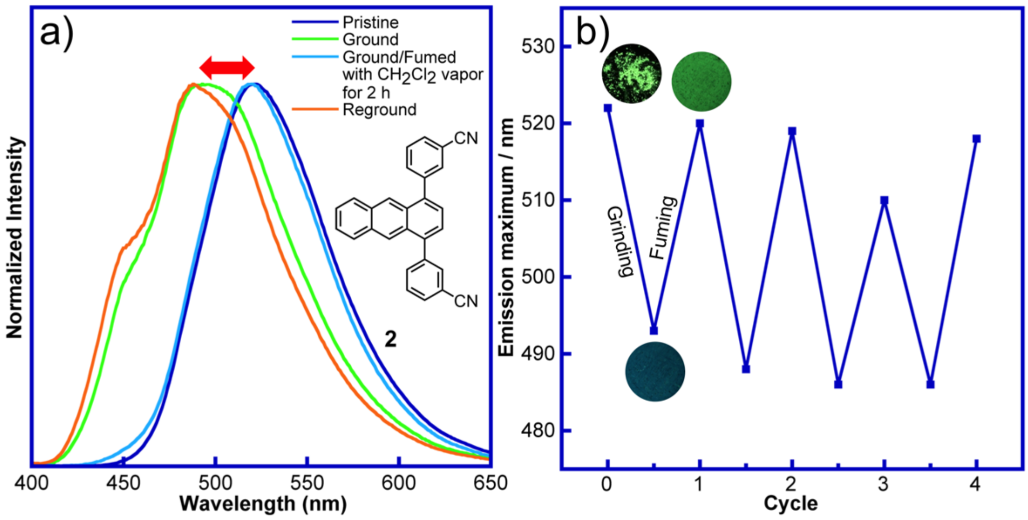

Figure S5. Vapochromic properties of 1,4-diphenylanthracene derivative **2** (3-CN), a) fluorescence spectra of pristine, ground, ground-fumed (CH_2Cl_2 vapor, 2 h) and reground samples, b) repeated grinding-fuming cycles.

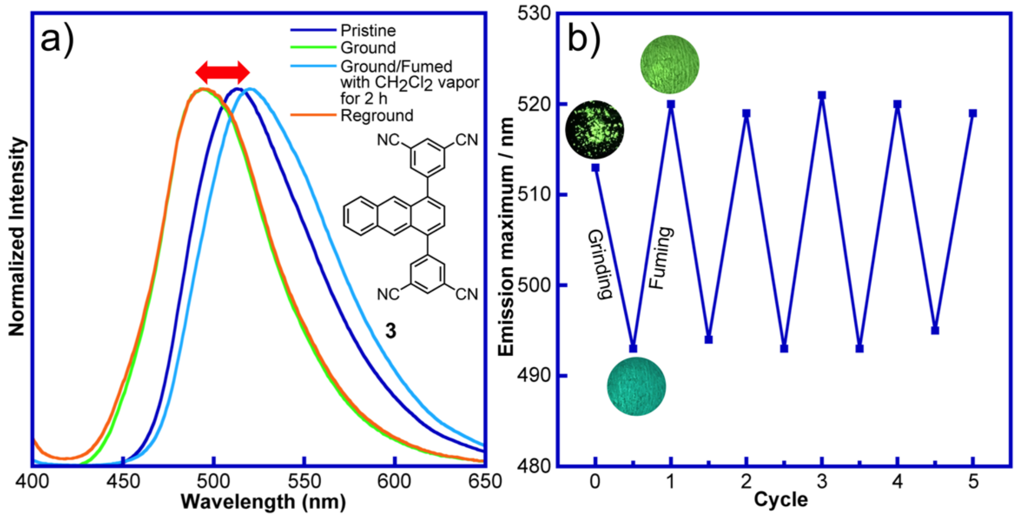

Figure S6. Vapochromic properties of 1,4-diphenylanthracene derivative **3** (3,5-CN_2, R'=CN), a) fluorescence spectra of pristine, ground, ground-fumed (CH_2Cl_2 vapor, 2 h) and reground samples, b) repeated grinding-fuming cycles.

1.5 Thermochromic properties of 1,4-diphenylanthracene derivatives

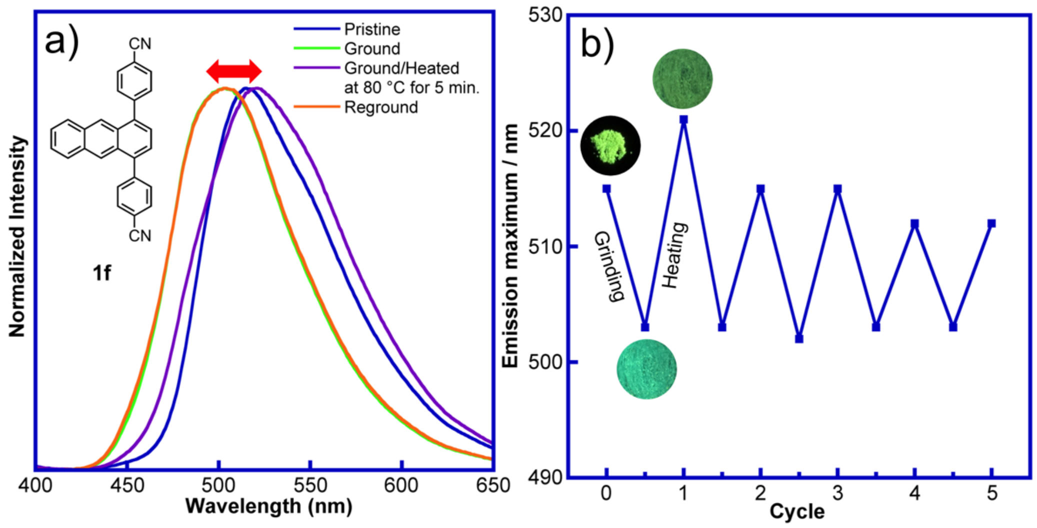

Figure S7. Thermochromic properties of 1,4-diphenylanthracene derivative **1f** (R=CN), a) fluorescence spectra of pristine, ground, ground-heated (80 °C for 5 min.) and reground samples, b) repeated grinding-heating cycles.

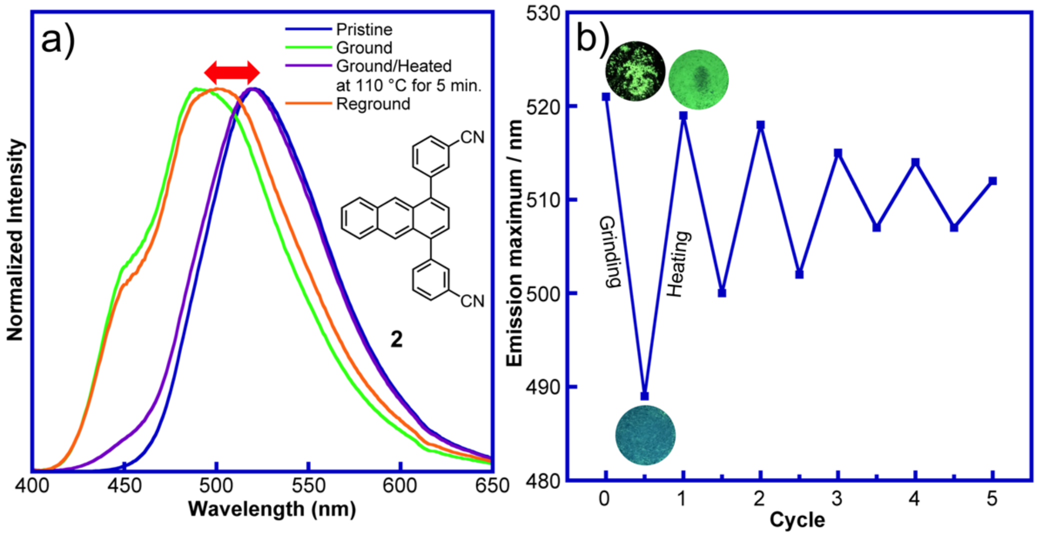

Figure S8. Thermochromic properties of 1,4-diphenylanthracene derivative **2** (3-CN), a) fluorescence spectra of pristine, ground, ground-heated (110 °C for 5 min.) and reground samples, b) repeated grinding-heating cycles.

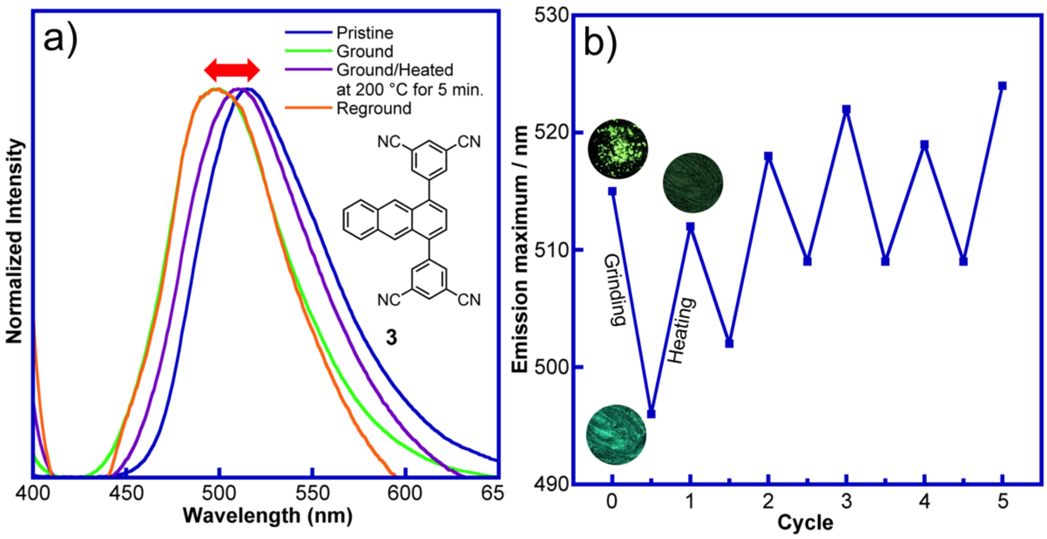

Figure S9. Thermochromic properties of 1,4-diphenylanthracene derivative **3** (3,5-CN_2, R'=CN), a) fluorescence spectra of pristine, ground, ground-heated (200 °C for 5 min.) and reground samples, b) repeated grinding-heating cycles.

1.6 IR spectra of the CN-substituted 1,4-diphenylanthracene derivatives 1f, 2

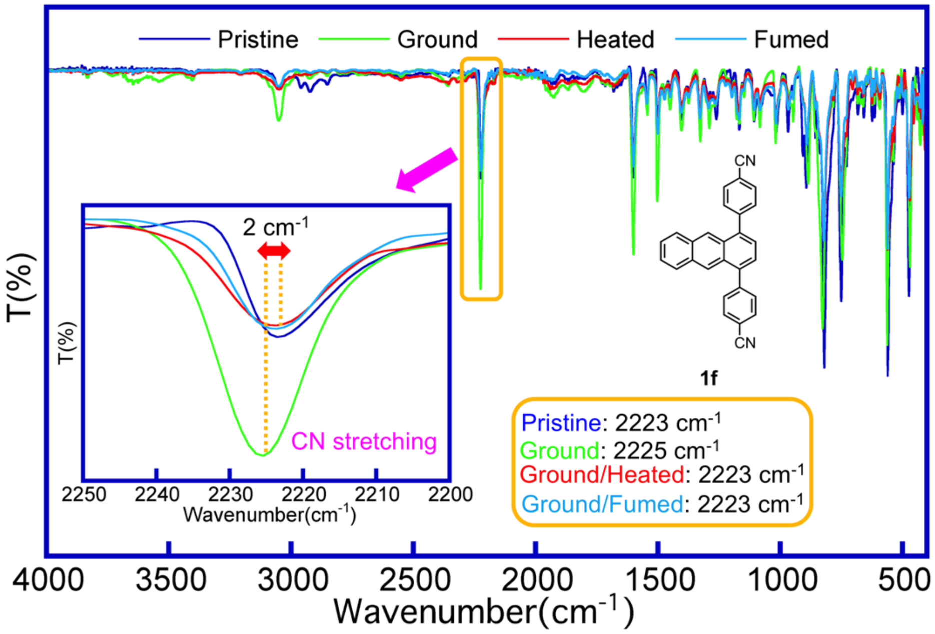

Figure S10. IR spectra of the CN-substituted 1,4-diphenylanthracene derivative **1f** before grinding (pristine), after grinding (ground), ground-heated (80 °C for 5 min) and ground-fumed (with CH_2Cl_2 vapor for 2 h) samples in the CN stretching region.

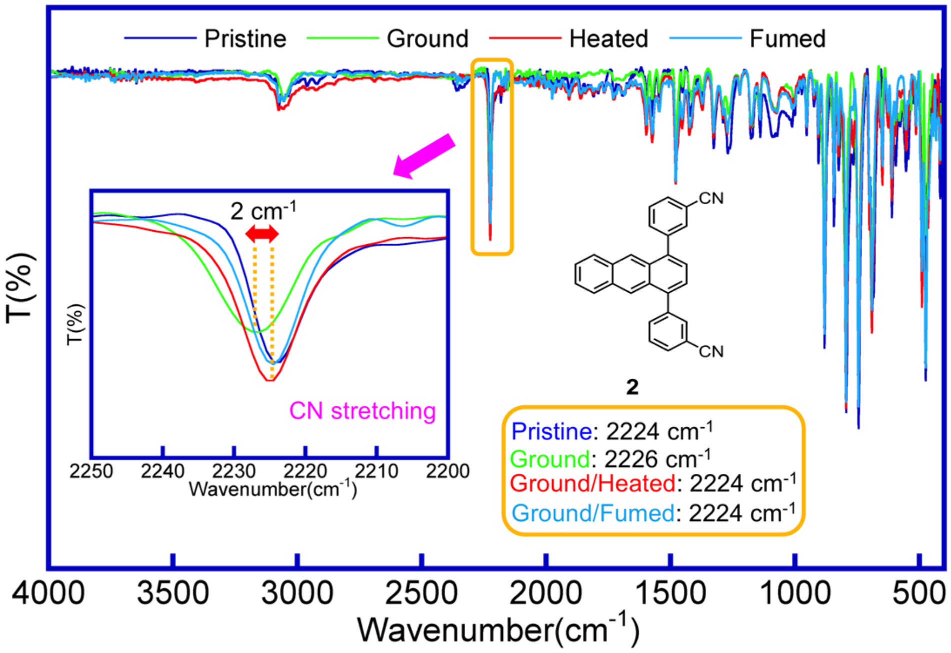

Figure S11. IR spectra of the CN-substituted 1,4-diphenylanthracene derivative **2** before grinding (pristine), after grinding (ground), ground-heated (110 °C for 5 min) and ground-fumed (with CH_2Cl_2 vapor for 2 h) samples in the CN stretching region.

1.7 DSC profiles of 1,4-diphenylanthracene derivatives

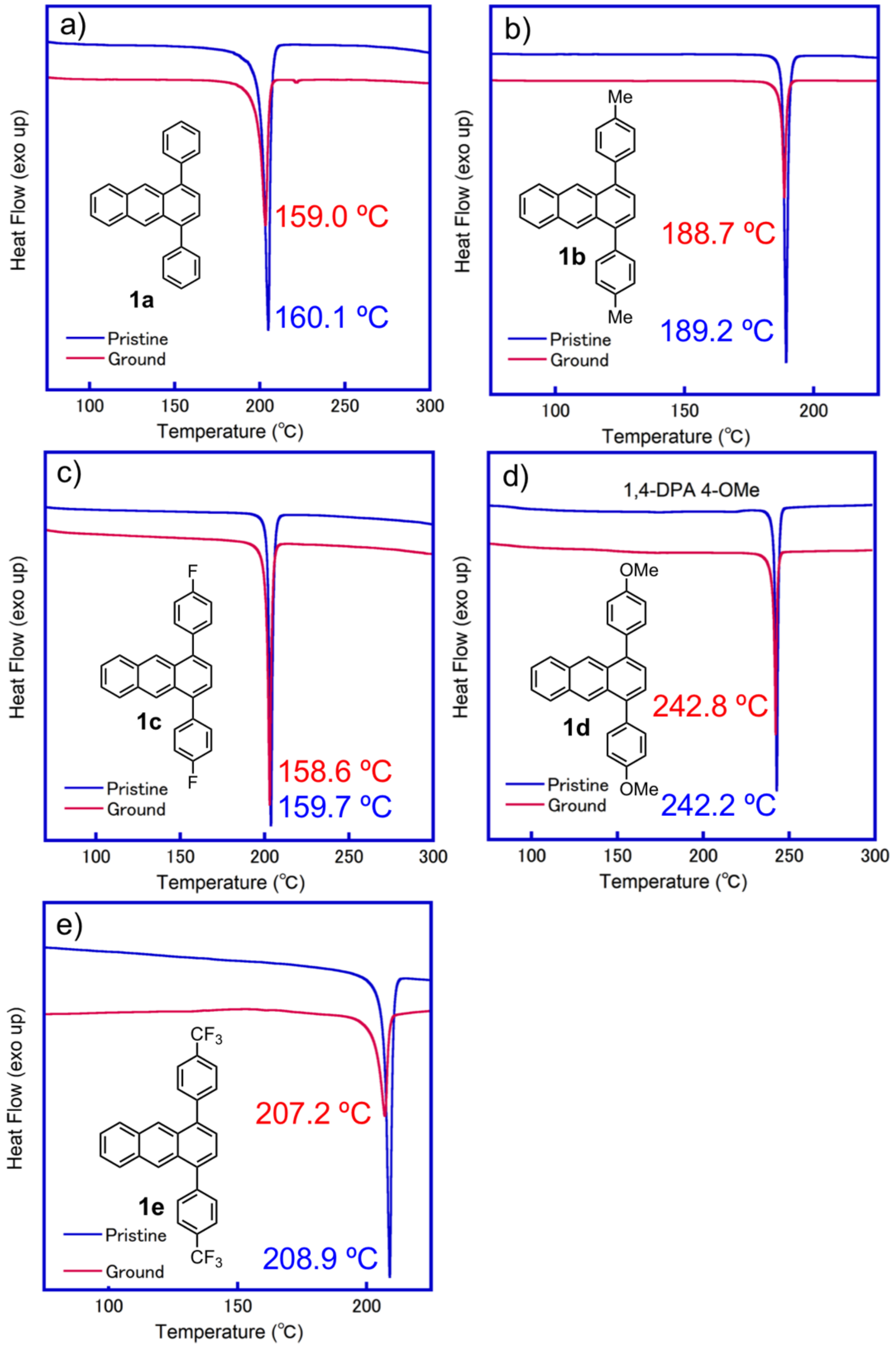

Figure S12. DSC profiles of 1,4-diphenylanthracene derivatives before grinding (pristine) and after grinding (ground), a) **1a** (R=H), b) **1b** (R=Me), c) **1c** (R=F), d) **1d** (R=OMe), e) **1e** (R=CF_3).

1.8 UV-vis absorption spectra of the pristine and ground samples

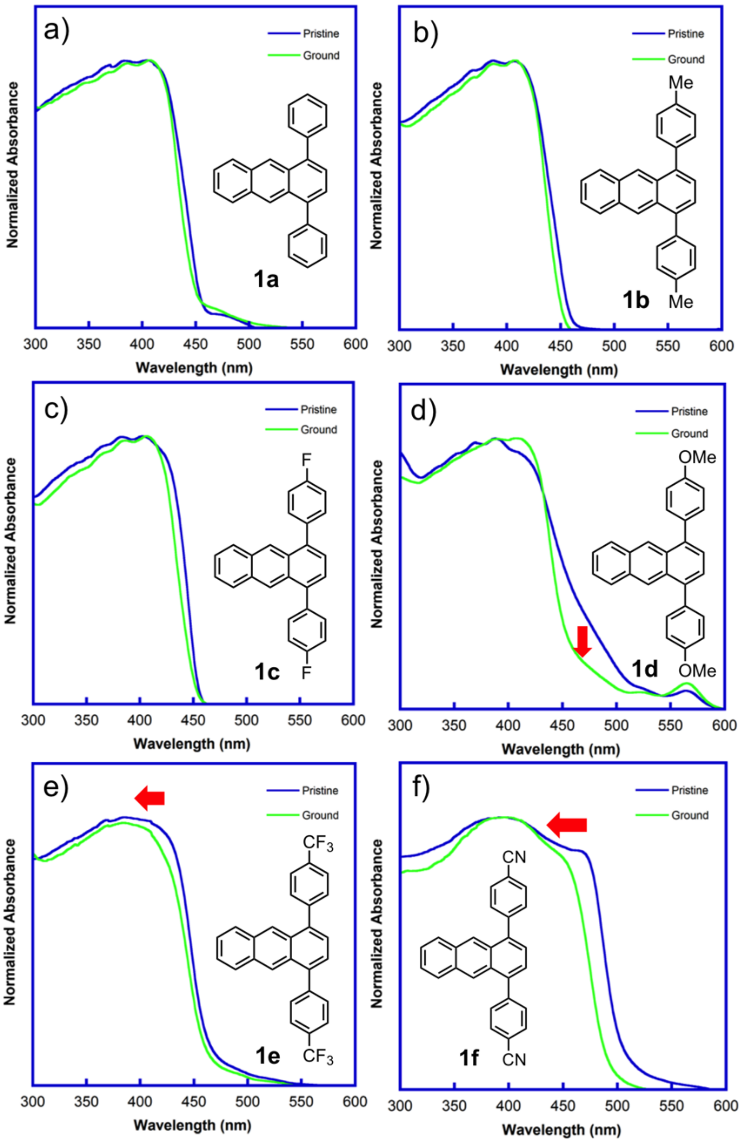

Figure S13. UV-vis absorption spectra of the pristine and ground samples of the 1,4-diphenylanthracene derivatives, a) **1a** (R=H), b) **1b** (R=Me), c) **1c** (R=F), d) **1d** (R=OMe), e) **1e** (R=CF_3), f) **1f** (R=CN).

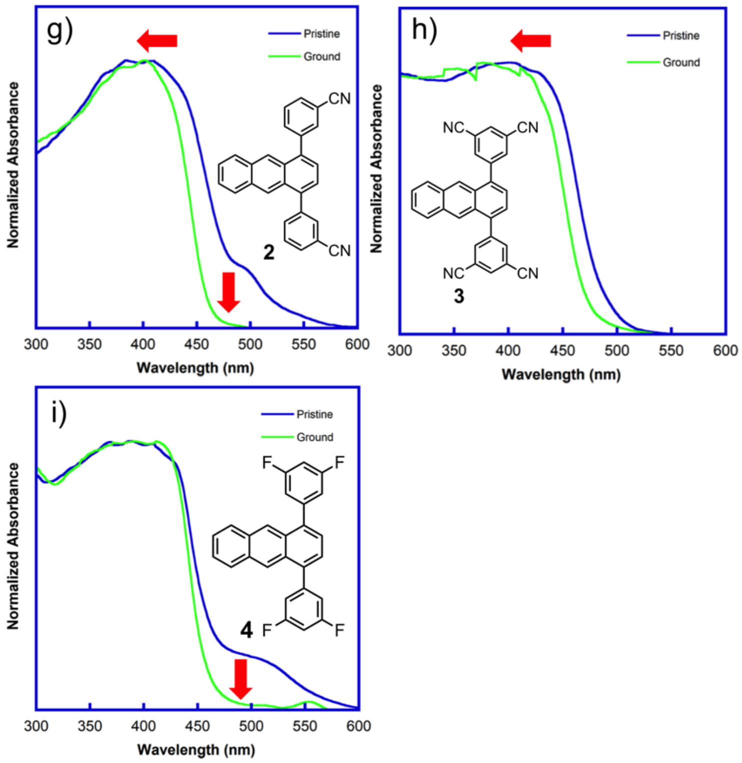

Figure S13 (contd.). UV-vis absorption spectra of the pristine and ground samples of the 1,4-diphenylanthracene derivatives, g) **2** (3-CN), h) **3** (3,5-CN_2, R'=CN), i) **4** (3,5-F_2, R'=F).

1.9 UV-vis absorption spectra of 1,4-diphenylanthracene derivatives (in $CHCl_3$)

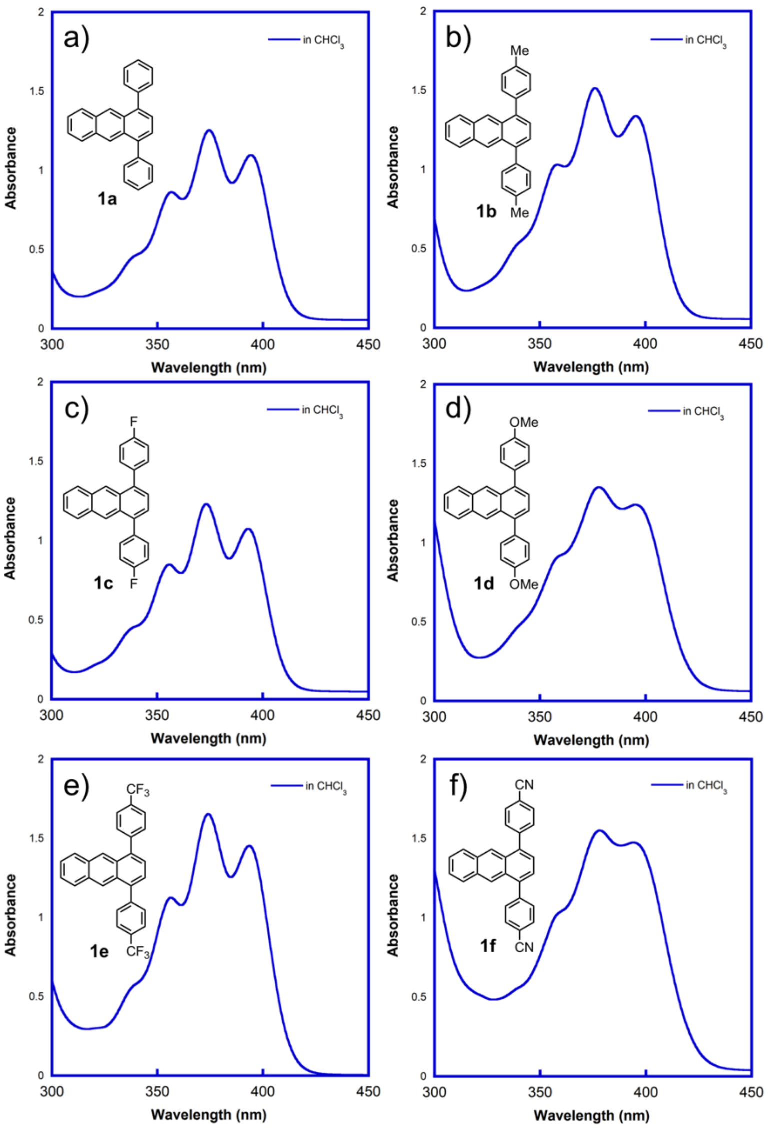

Figure S14. UV-vis absorption spectra of 1,4-diphenylanthracene derivatives (0.1 mM in $CHCl_3$), a) **1a** (R=H), b) **1b** (R=Me), c) **1c** (R=F), d) **1d** (R=OMe), e) **1e** (R=CF_3), f) **1f** (R=CN).

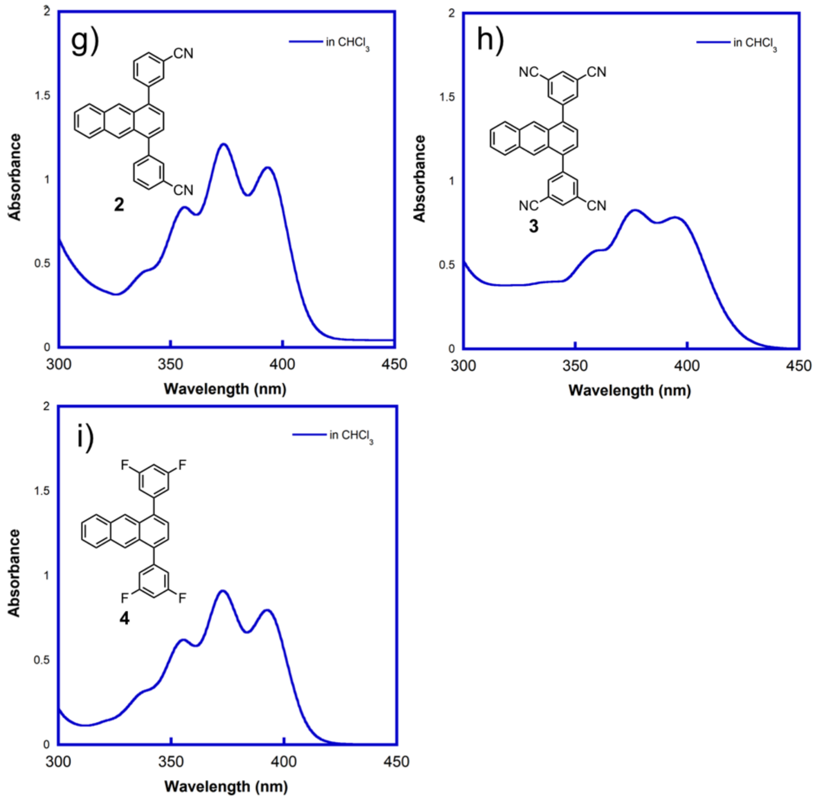

Figure S14 (contd.). UV-vis absorption spectra of 1,4-diphenylanthracene derivatives (0.1 mM in $CHCl_3$), g) **2** (3-CN), h) **3** (3,5-CN_2, R'=CN), i) **4** (3,5-F_2, R'=F).

1.10 Fluorescence spectra of 1,4-diphenylanthracene derivatives (in $CHCl_3$)

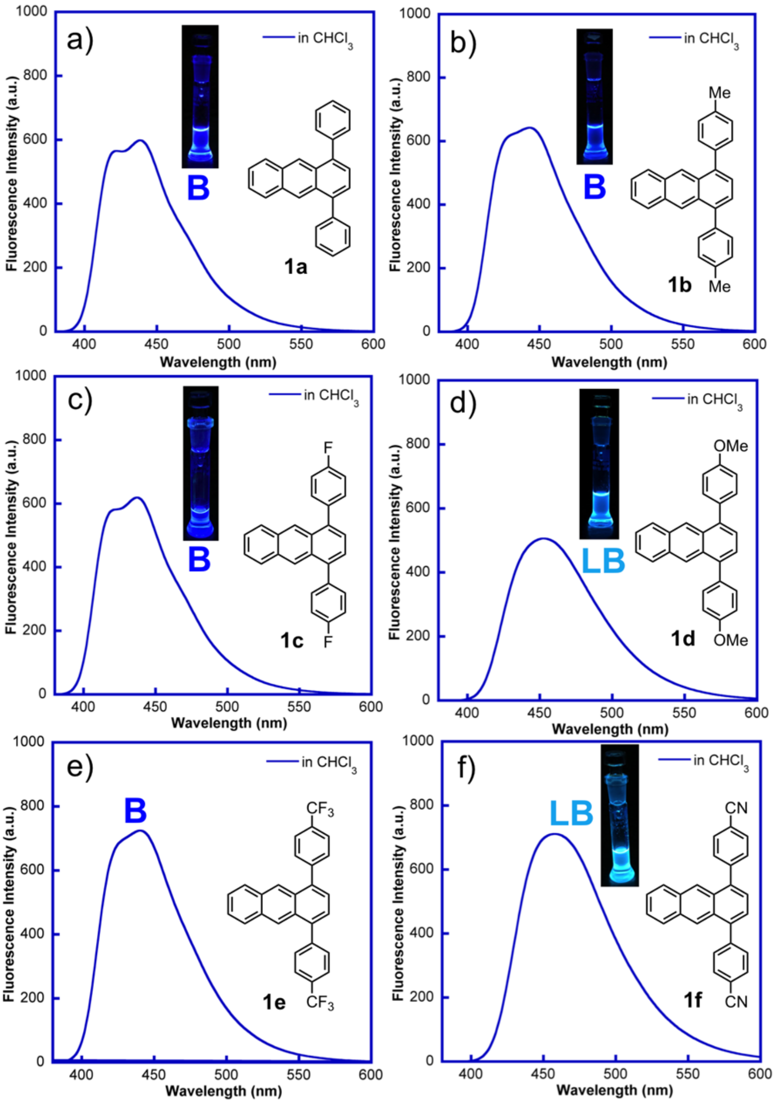

Figure S15. Fluorescence spectra of 1,4-diphenylanthracene derivatives (25 μM in $CHCl_3$, λ_{ex} = 365 nm): a) **1a** (R=H), b) **1b** (R=Me), c) **1c** (R=F), d) **1d** (R=OMe), e) **1e** (R=CF_3), f) **1f** (R=CN).

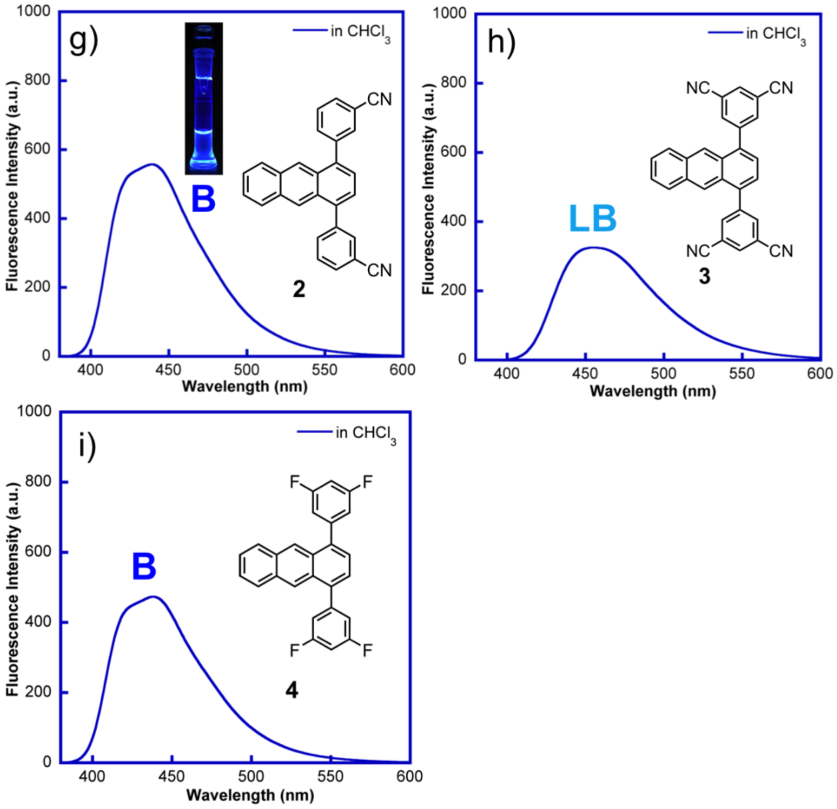

Figure S15 (contd.). Fluorescence spectra of 1,4-diphenylanthracene derivatives (25 μM in $CHCl_3$, λ_{ex} = 365 nm): g) **2** (3- CN), h) **3** (3,5-CN_2, R'=CN), i) **4** (3,5-F_2, R'=F).

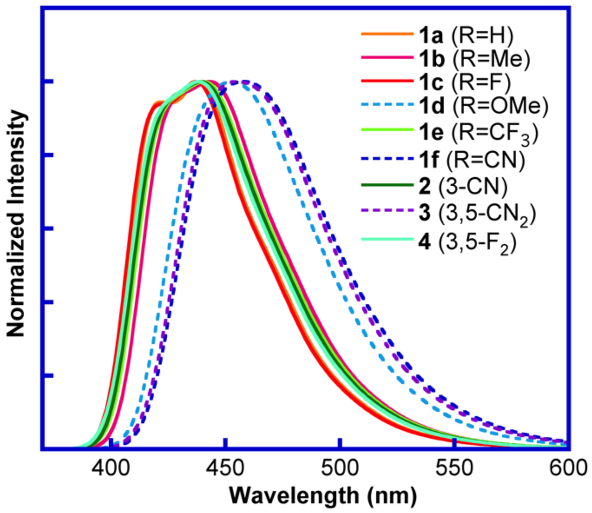

Figure S16. Fluorescence spectra of 1,4-diphenylanthracene derivatives (25 μM in $CHCl_3$, λ_{ex} = 365 nm).

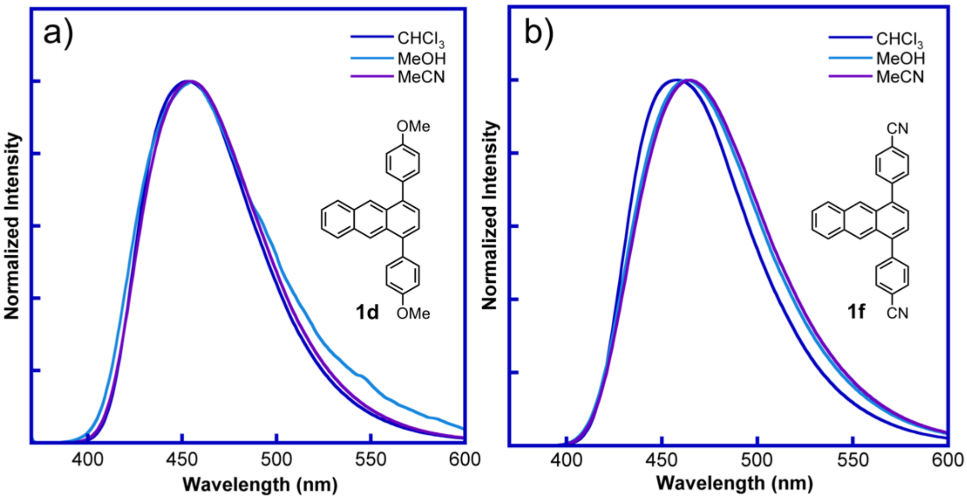

Figure S17. Fluorescence spectra of 1,4-diphenylanthracene derivatives (25 μM in different solvents, λ_{ex} = 365 nm): a) **1d** (R=OMe), b) **1f** (R=CN).

1.11 Fluorescence spectra of 1,4-diphenylanthracene derivatives in PMMA film

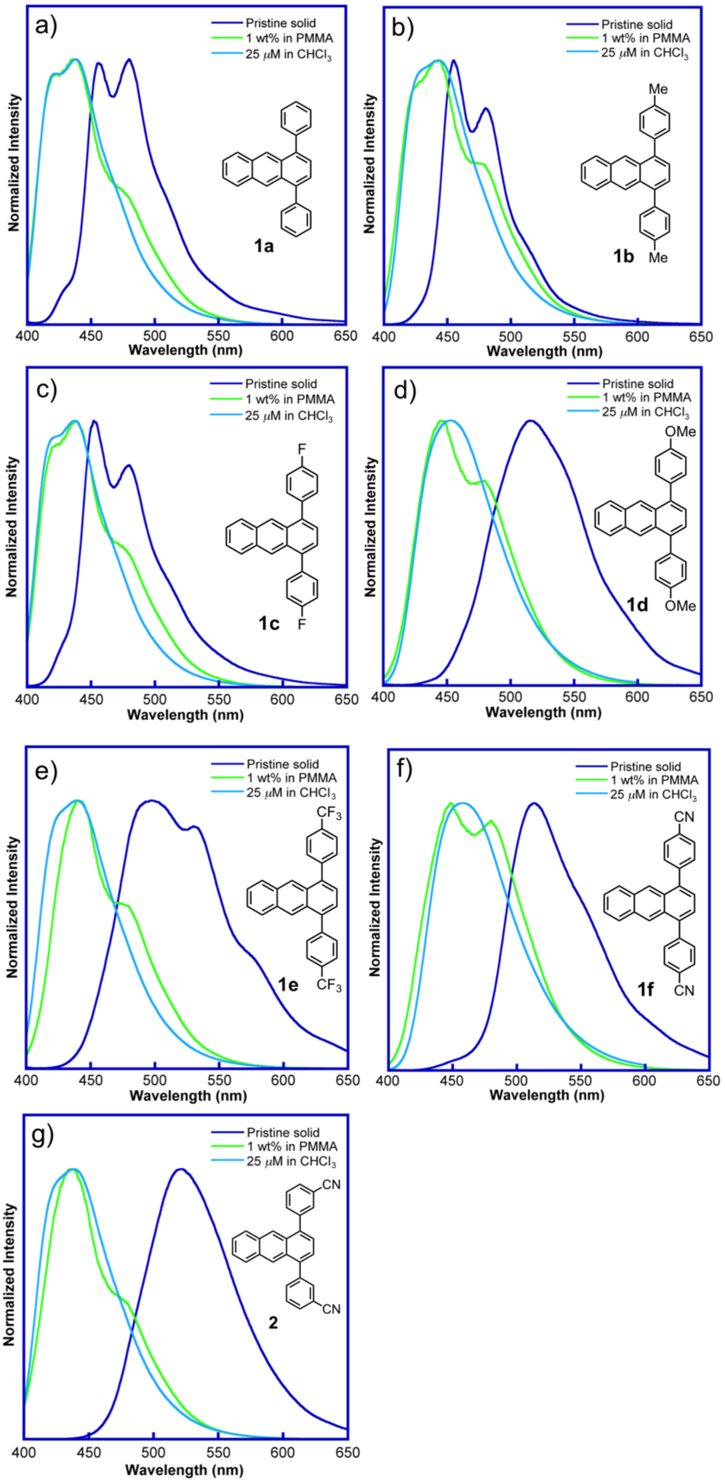

Figure S18. Fluorescence spectra of 1,4-diphenylanthracene derivatives in pristine solid, 25 μM in $CHCl_3$ solution, 1 wt% in PMMA film (λ_{ex} = 365 nm), a) **1a** (R=H), b) **1b** (R=Me), c) **1c** (R=F), d) **1d** (R=OMe), e) **1e** (R=CF_3), f) **1f** (R=CN), g) **2** (3-CN).

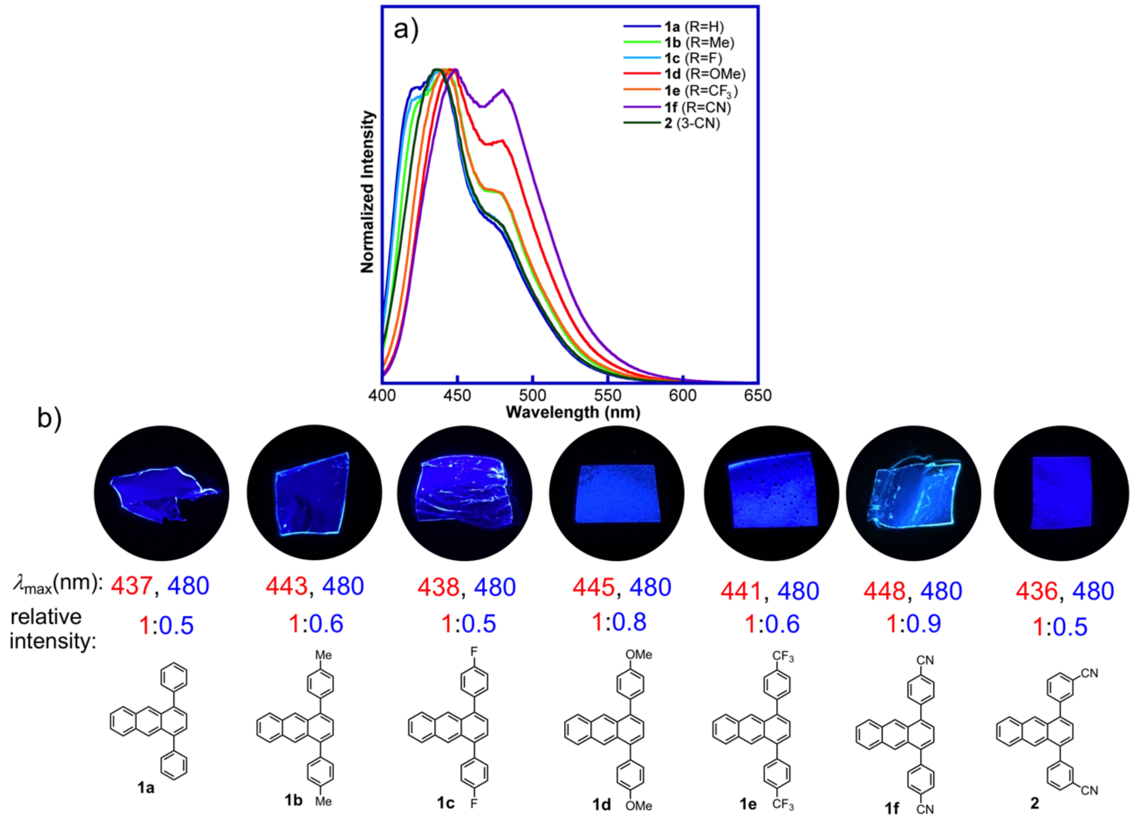

Figure S19. Fluorescence spectra and photographs of 1,4-diphenylanthracene derivatives in PMMA film (1 wt%), a) comparison of normalized fluorescence spectra (λ_{ex} = 365 nm), b) photographs in PMMA film (under 365 nm UV light) and relative intensities of fluorescence spectra.

1.12 Fluorescence lifetime decay profiles of pristine, ground, ground-heated and ground-fumed samples of 1,4-diphenylanthracene derivatives

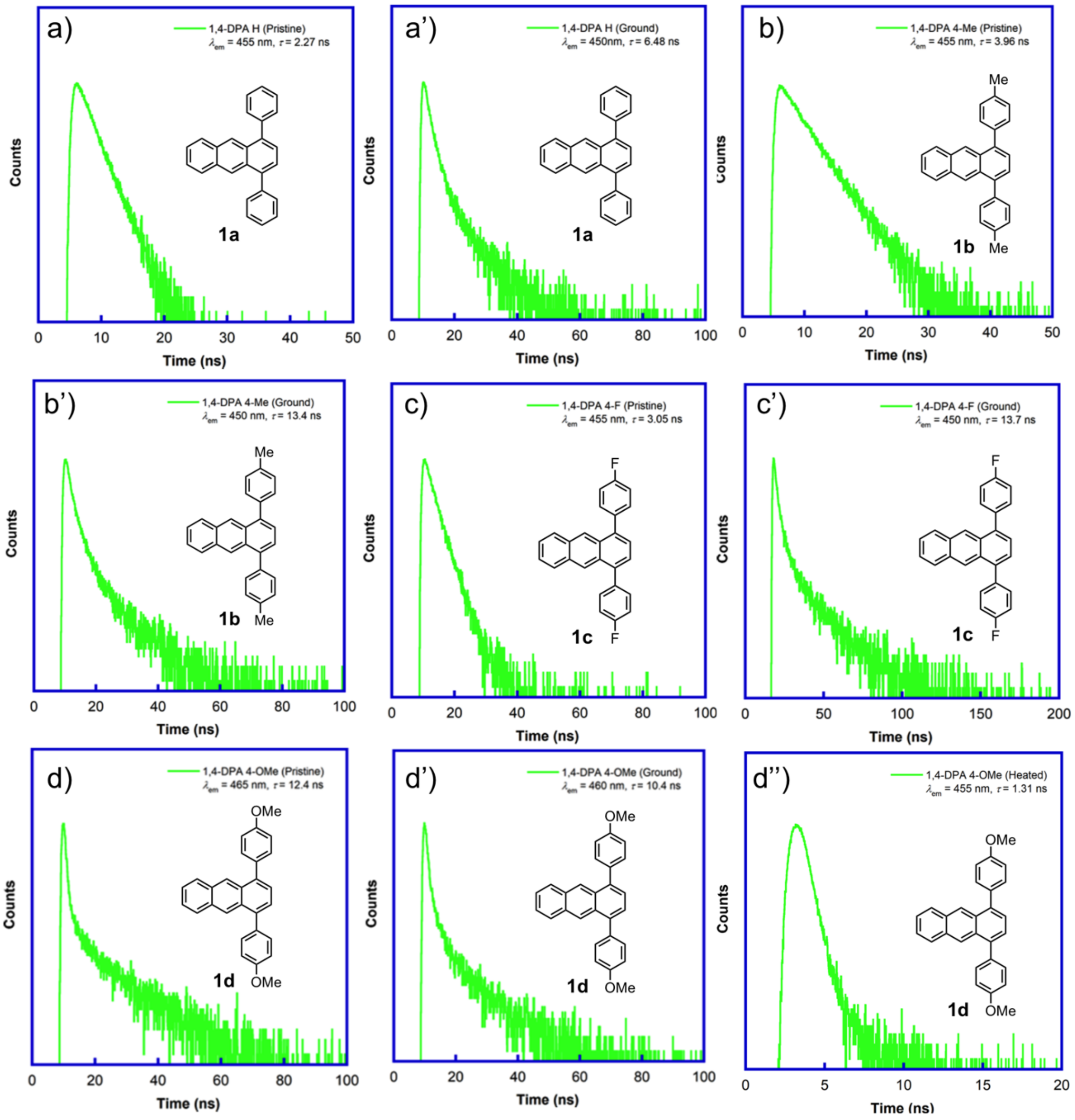

Figure S20. Fluorescence lifetime decay profile of pristine, ground, ground-heated and ground-fumed samples of 1,4-diphenylanthracene derivatives (λ_{ex} = 365 nm): a) **1a** (R=H, pristine), a') **1a** (R=H, ground), b) **1b** (R=Me, pristine), b') **1b** (R=Me, ground), c) **1c** (R=F, pristine), c') **1c** (R=F, ground), d) **1d** (R=OMe, pristine), d') **1c** (R=OMe, ground), d'') **1d** (R=OMe, ground-heated, 150 °C for 5 min.).

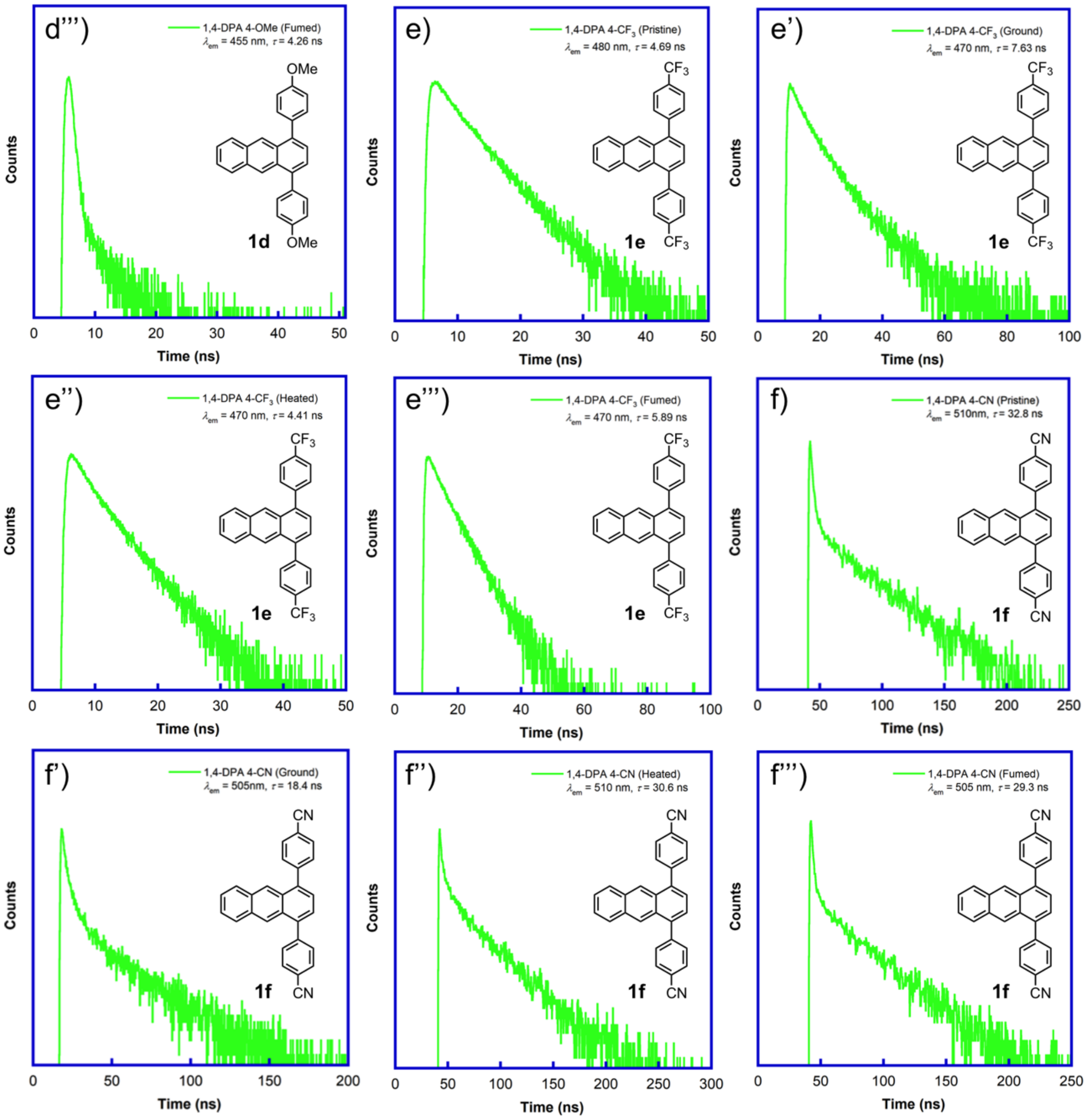

Figure S20 (contd.). Fluorescence lifetime decay profile of pristine, ground, ground-heated and ground-fumed samples of 1,4-diphenylanthracene derivatives (λ_{ex} = 365 nm): d''') **1c** (R=OMe, ground-fumed, CH_2Cl_2 vapor for 20 h), e) **1e** (R=CF_3, pristine), e') **1e** (R=CF_3, ground), e'') **1e** (R=CF_3, ground-heated, 150 °C, 5 min.), e''') **1e** (R=CF_3, ground-fumed, CH_2Cl_2 vapor for 20 h), f) **1f** (R=CN, pristine), f') **1f** (R=CN, ground), f'') **1f** (R=CN, ground-heated, 80 °C, 5 min.), f''') **1f** (4-CN, ground-fumed, CH_2Cl_2 vapor for 2 h).

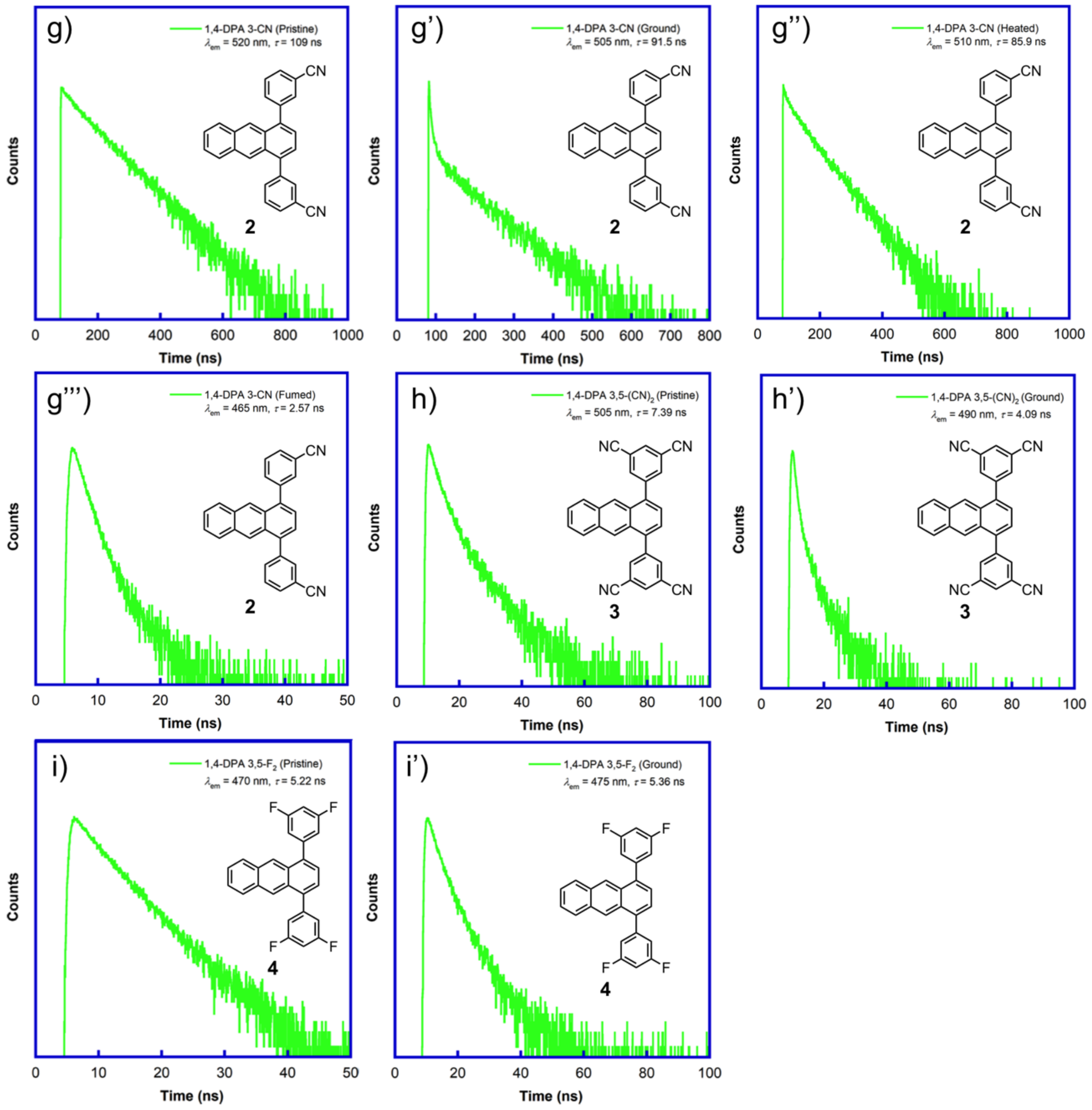

Figure S20 (cont.). Fluorescence lifetime decay profile of pristine, ground, ground-heated and ground-fumed samples of 1,4-diphenylanthracene derivatives (λ_{ex} = 365 nm): g) **2** (3-CN, pristine), g') **2** (3-CN, ground), g'') **2** (3-CN, ground-heated, 110 °C, 5 min.), g''') **2** (3-CN, ground-fumed, CH_2Cl_2 vapor for 2h), h) **3** (3,5-CN_2, R'=CN, pristine), h') **3** (3,5-CN_2, R'=CN, ground), i) **4** (3,5-F_2, R'=F, pristine), i') **4** (3,5-F_2, R'=F, ground).

1.13 Fluorescence lifetime decay profiles of 1,4-diphenylanthracene derivatives in $CHCl_3$ solution

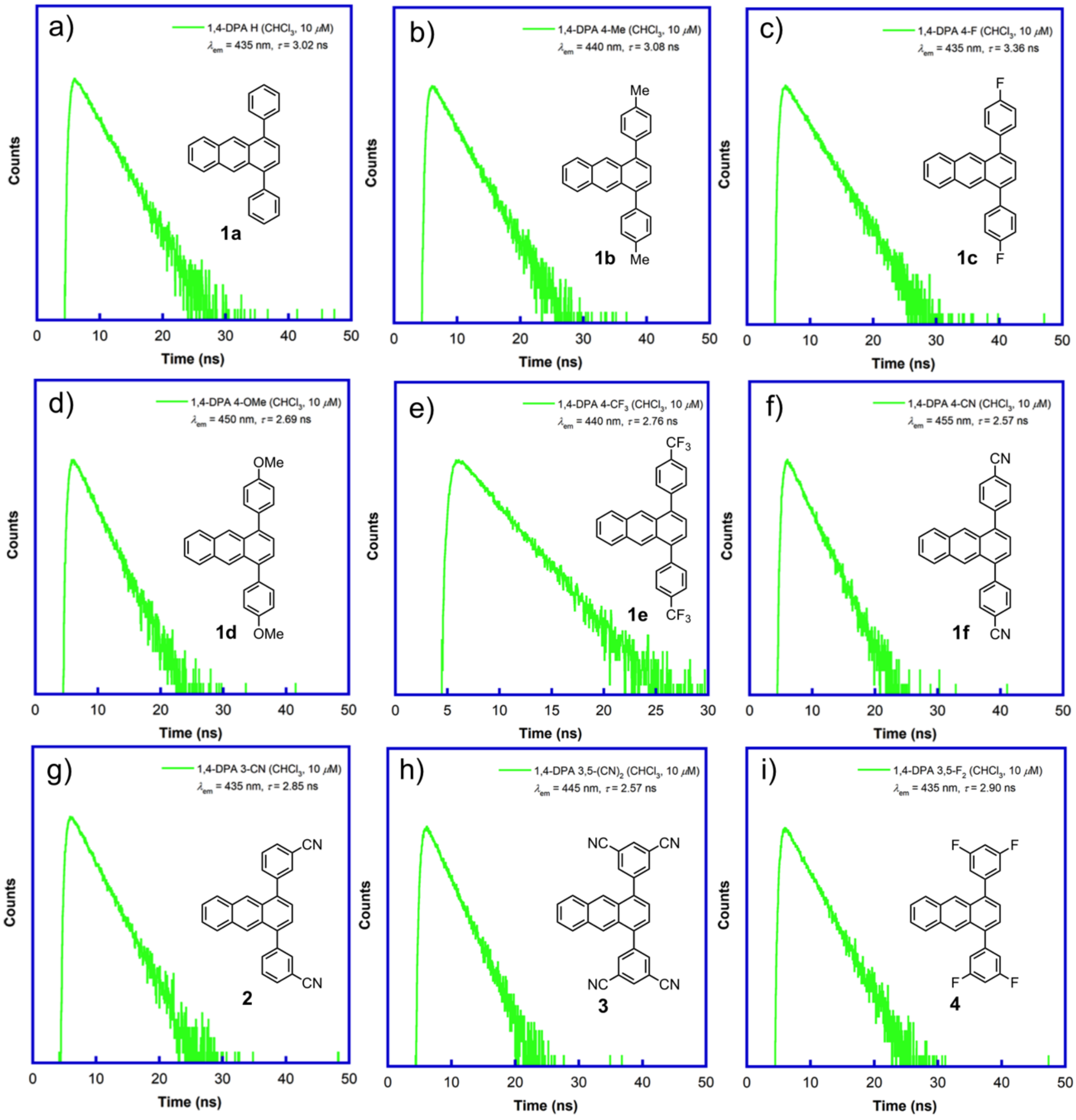

Figure S21. Fluorescence lifetime decay profile of 1,4-diphenylanthracene derivatives in $CHCl_3$ (10 μM, λ_{ex} = 365 nm) after Ar bubbling: a) **1a** (R=H), b) **1b** (R=Me), c) **1c** (R=F), d) **1d** (R=OMe), e) **1e** (R=CF_3), f) **1f** (R=CN), g) **2** (3-CN), h) **3** (3,5-CN_2, R'=CN), i) **4** (3,5-F_2, R'=F).

1.14 Fluorescence lifetime decay profiles of 1,4-diphenylanthracene derivatives in PMMA film

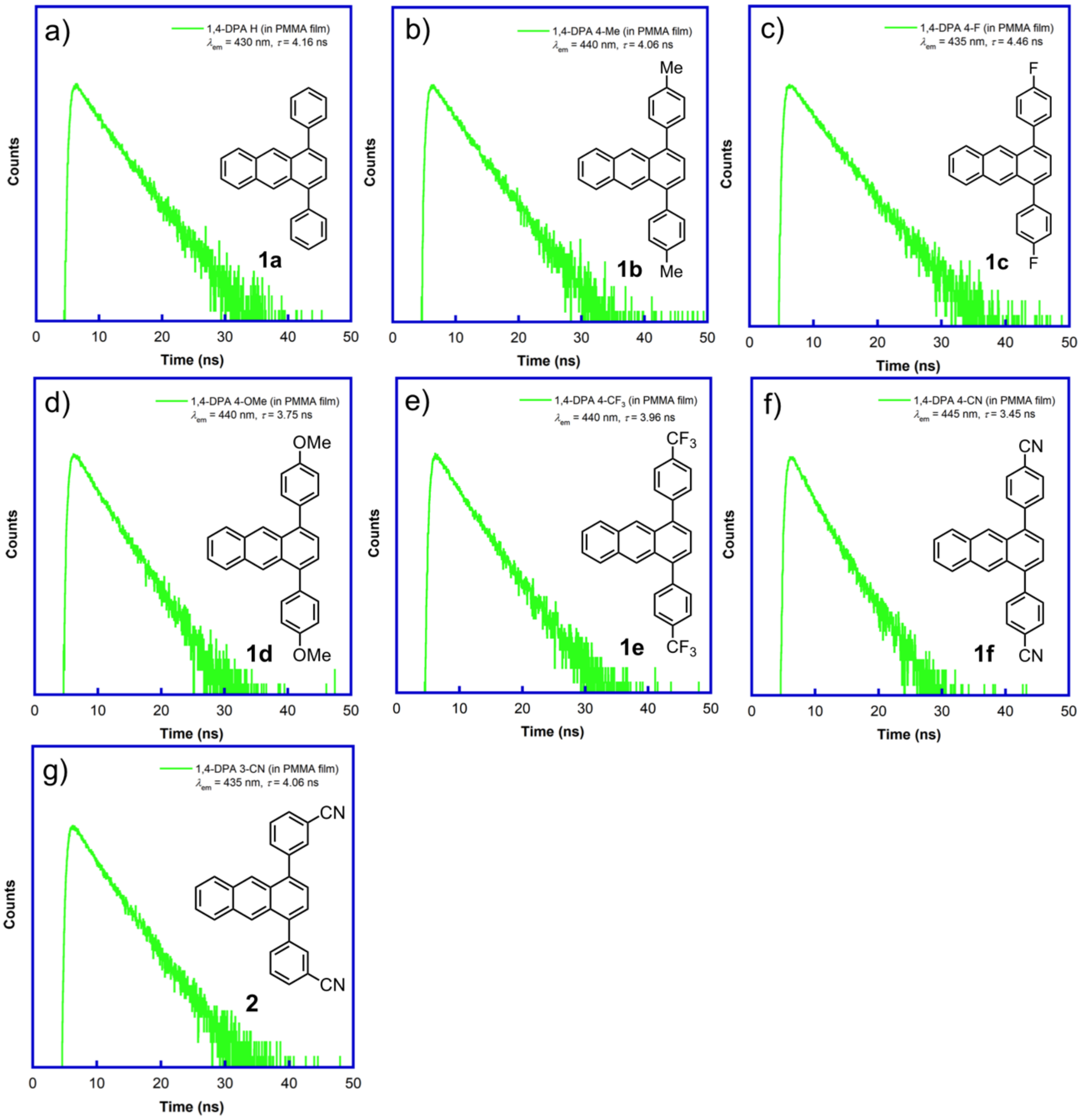

Figure S22. Fluorescence lifetime decay profiles of 1,4-diphenylanthracene derivatives in PMMA film (1 wt%, λ_{ex} = 365 nm): a) **1a** (R=H), b) **1b** (R=Me), c) **1c** (R=F), d) **1d** (R=OMe), e) **1e** (R=CF_3), f) **1f** (R=CN), g) **2** (3-CN).

1.15 Photophysical properties of 1,4-diphenylanthracene derivatives

Table S1. Photophysical properties of 1,4-diphenylanthracene derivatives

compds.	ϕ_f[a]		τ[b] / ns		k_f / ns^{-1}		k_{nr} / ns^{-1}	
	soln.[c]	pristine (ground)	soln.[c]	pristine (ground)	soln.	pristine (ground)	soln.	pristine (ground)
**1a** (R=H)	0.47	0.20 (0.23)	3.02	2.27 (6.48)	0.156	0.088 (0.035)	0.175	0.352 (0.119)
**1b** (R=Me)	0.49	0.31 (0.21)	3.08	3.96 (13.4)	0.159	0.078 (0.016)	0.166	0.174 (0.059)
**1c** (R=F)	0.49	0.24 (0.25)	3.36	3.05 (13.7)	0.146	0.079 (0.018)	0.152	0.249 (0.055)
**1d** (R=OMe)	0.40	0.15 (0.18)	2.69	12.4 (10.4)	0.149	0.012 (0.017)	0.223	0.069 (0.079)
**1e** (R=CF_3)	0.51	0.37 (0.33)	2.76	4.69 (7.63)	0.185	0.079 (0.043)	0.178	0.134 (0.088)
**1f** (R=CN)	0.55	0.26 (0.33)	2.57	32.8 (18.4)	0.214	0.008 (0.018)	0.175	0.023 (0.036)
**2** (3-CN)	0.46	0.71 (0.37)	2.85	109 (91.5)	0.161	0.007 (0.004)	0.189	0.003 (0.007)
**3** (3,5-CN_2)	0.41	0.15 (0.05)	2.57	7.39 (4.09)	0.160	0.020 (0.012)	0.230	0.115 (0.232)
**4** (3,5-F_2)	0.50	0.44 (0.22)	2.90	5.22 (5.36)	0.172	0.084 (0.041)	0.172	0.107 (0.146)

[a] Fluorescence quantum yield determined by a calibrated integrating sphere, [b] Averaged fluorescence lifetimes, [c] $CHCl_3$ solution (10 μM) after Ar bubbling.

Table S2. Photophysical properties of 1,4-diphenylanthracene derivatives in PMMA film

PMMA films	λ_{max}/nm[a], Luminous color[b]		ϕ_f[c]	τ[d] / ns	k_f[e] / ns^{-1}	k_{nr}[f] / ns^{-1}
**1a** (R=H)	437	B	0.64	4.16	0.154	0.087
**1b** (R=Me)	443	B	0.66	4.06	0.163	0.084
**1c** (R=F)	438	B	0.63	4.42	0.143	0.084
**1d** (R=OMe)	445	B	0.67	3.75	0.179	0.088
**1e** (R=CF_3)	441	B	0.64	3.96	0.162	0.091
**1f** (R=CN)	448	LB	0.71	3.45	0.206	0.084
**2** (3-CN)	436	B	0.62	4.06	0.153	0.094

[a]Solid-state (1wt% in PMMA film) emission (λ_{ex}=365 nm), [b]Luminous color under 365 nm UV irradiation, B: blue, LB: light blue, [c]Fluorescence quantum yield determined by a calibrated integrating sphere at λ_{ex}=365 nm, [d]Averaged fluorescence lifetime at λ_{ex}=365 nm, [e]Rate constant for fluorescence, [f]Rate constant for nonradiative decay.

2. X-ray analysis of 1,4-diphenylanthracene derivatives
2.1. Single crystal X-ray analysis

Table S3. Crystallographic data for **1a-1d**

Compounds	**1a** (R=H)	**1b** (R=Me)	**1c** (R=F)	**1d** (R=OMe)
Chemical formula	$C_{26}H_{18}$	$C_{28}H_{22}$	$C_{26}H_{16}F_2$	$C_{28}H_{22}O_2$
Temperature /K	293	100	100	100
Crystal system	orthorhombic	orthorhombic	triclinic	orthorhombic
Space group	$P2_12_12_1$	$P2_12_12_1$	P-1	$P2_12_12_1$
a /Å	5.8982(11)	5.9154(2)	5.8772(2)	5.9797(3)
b /Å	14.477(4)	13.6349(3)	11.5283(6)	13.9553(7)
c /Å	20.400(4)	23.8451(6)	14.1198(7)	23.2841(10)
α /°			113.856(2)	
β /°			95.664(2)	
γ /°			90.875(2)	
V /Å^3	1741.9(7)	1923.25(9)	869.07(7)	1943.02(16)
Z	4	4	2	4
Radiation type	MoK$_\alpha$	MoK$_\alpha$	MoK$_\alpha$	MoK$_\alpha$
$D_{calcd.}$ /Mgm^{-3}	1.260	1.238	1.400	1.335
Collected reflections	5485	19998	11351	23611
Unique reflections	3191	3916	3032	3959
R_{int}	0.0488	0.0485	0.0369	0.0794
R_1 ($I > 2\sigma(I)$)	0.0712	0.0395	0.0393	0.0476
R_1 (all data)	0.1368	0.0425	0.0452	0.0585
R_w (all data)	0.2276	0.1045	0.1067	0.1186
Goodness-of fit	1.007	1.018	1.029	1.046

Table S4. Crystallographic data for **1f** and **2**

Compounds	**1f** (R=CN)	**2** (3-CN)
Chemical formula	$C_{28}H_{16}N_2$	$C_{28}H_{16}N_2$
Temperature /K	100	293
Crystal system	triclinic	triclinic
Space group	*P*-1	*P*-1
a /Å	11.0734(2)	8.2867(7)
b /Å	12.9373(2)	10.2528(8)
c /Å	14.2271(2)	12.4967(11)
α /°	80.5840(10)	75.726(7)
β /°	86.7280(10)	78.035(7)
γ /°	75.6040(10)	82.179(7)
V /Å^3	1974.29(5)	1002.56(15)
Z	4	2
Radiation type	CuK$_\alpha$	MoK$_\alpha$
$D_{calcd.}$ /Mgm^{-3}	1.298	1.260
Collected reflections	18220	6102
Unique reflections	6871	4100
R_{int}	0.0513	0.0254
R_1 ($I > 2\sigma(I)$)	0.0470	0.0579
R_1 (all data)	0.0633	0.1105
R_w (all data)	0.1327	0.1691
Goodness-of fit	1.016	1.031

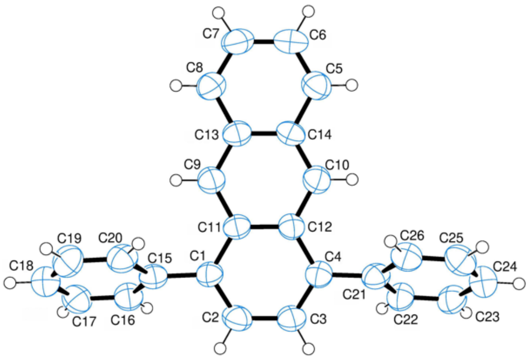

Figure S23. ORTEP drawing of **1a** (R=H).

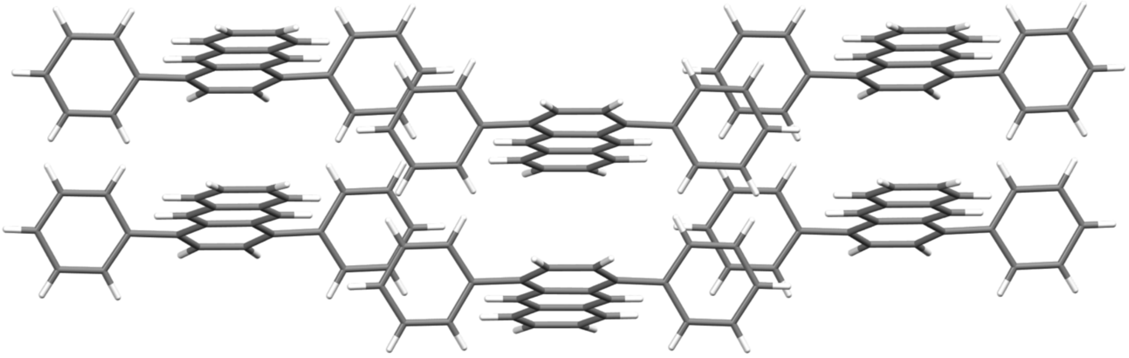

Figure S24. Crystal packing of **1a** (R=H).

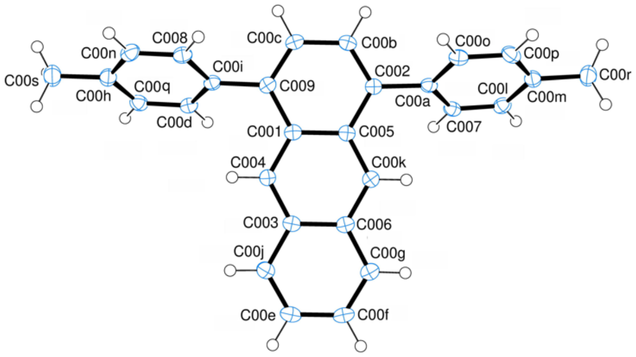

Figure S25. ORTEP drawing of **1b** (R=Me).

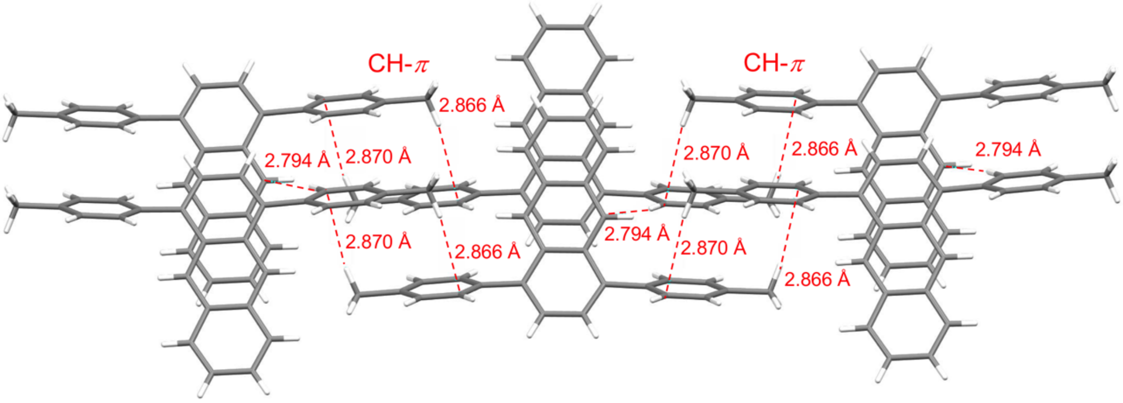

Figure S26. Crystal packing of **1b** (R=Me).

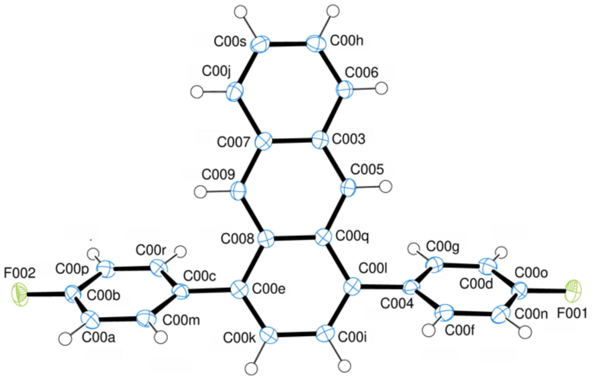

Figure S27. ORTEP drawing of **1c** (R=F).

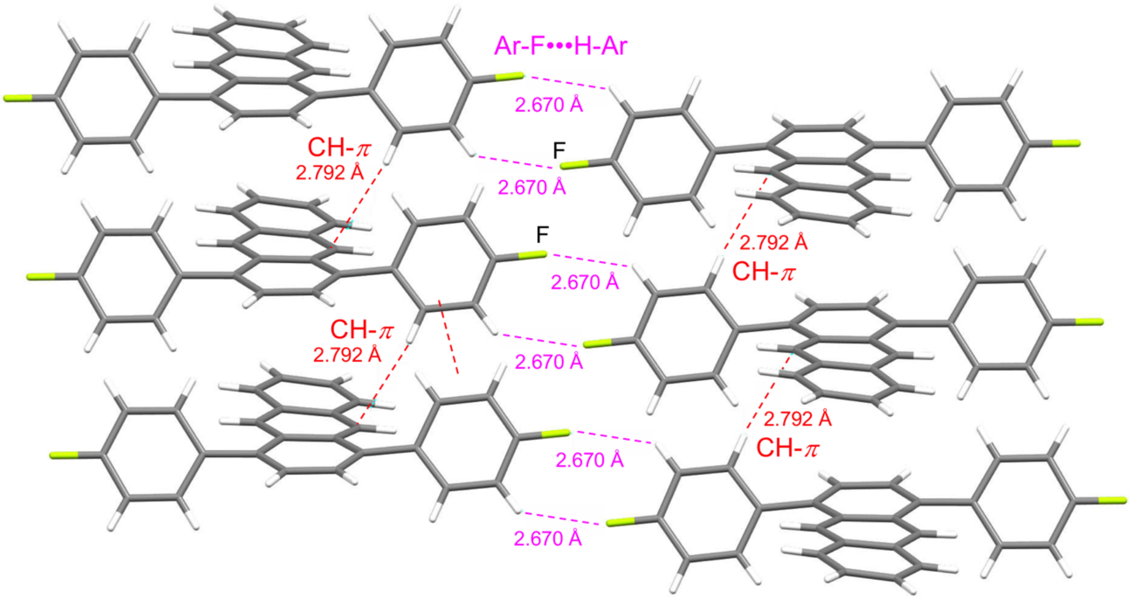

Figure S28. Crystal packing of **1c** (R=F).

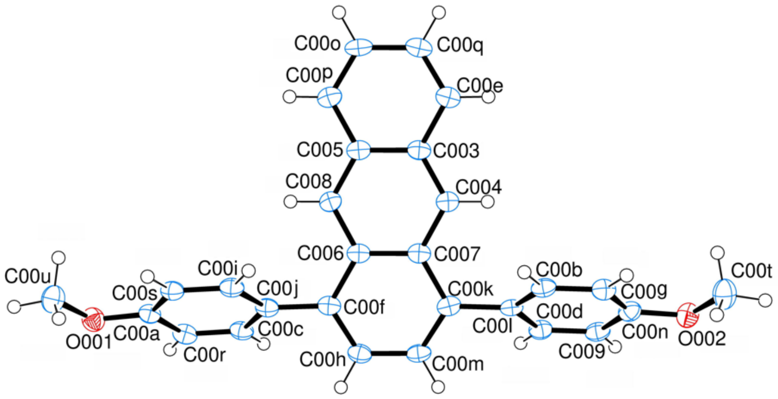

Figure S29. ORTEP drawing of **1d** (R=OMe).

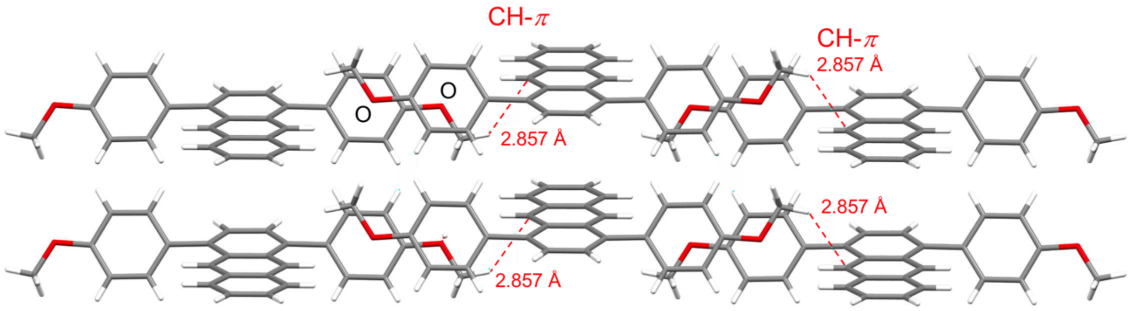

Figure S30. Crystal packing of **1d** (R=OMe).

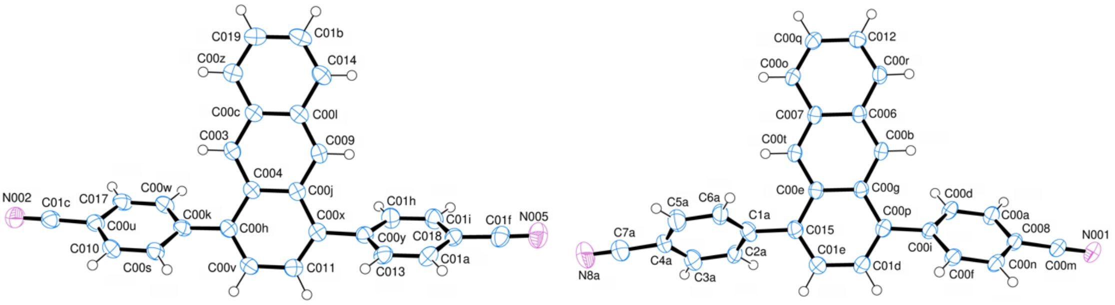

Figure S31. ORTEP drawing of **1f** (R=CN).

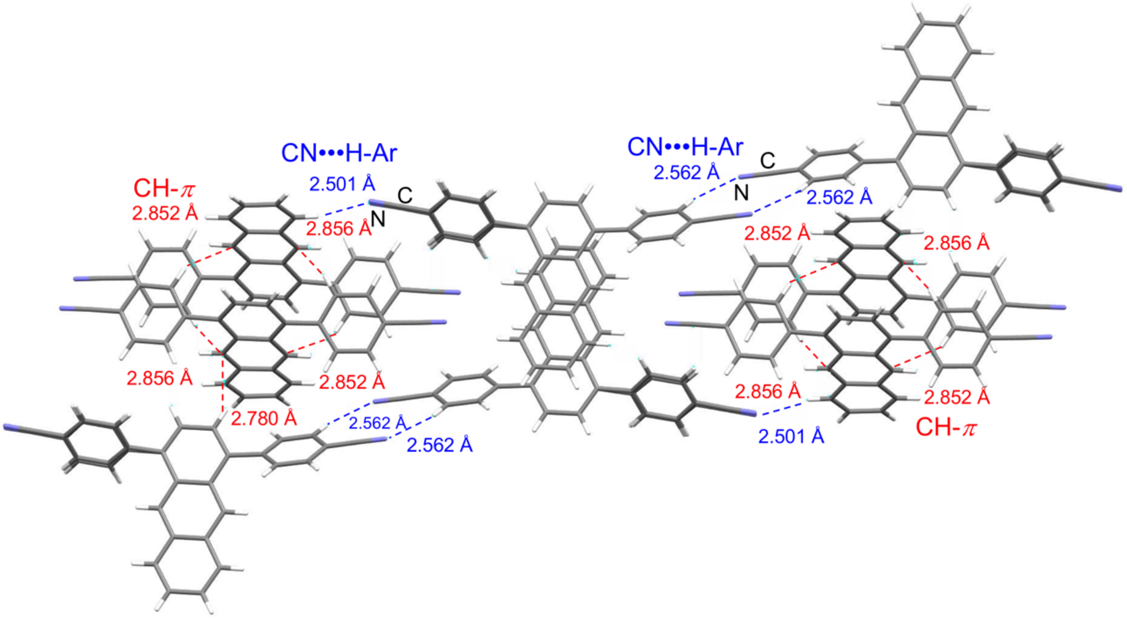

Figure S32. Crystal packing of **1f** (R=CN).

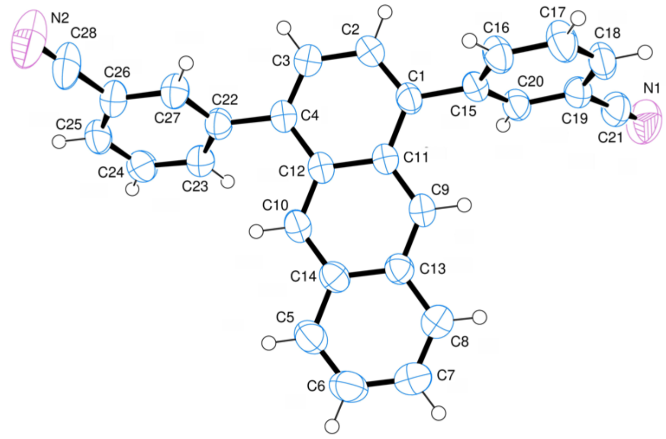

Figure S33. ORTEP drawing of **2** (3-CN).

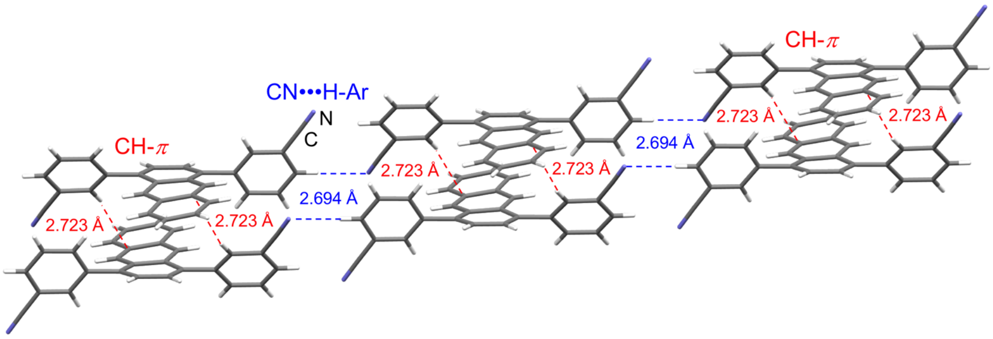

Figure S34. Crystal packing of **2** (3-CN).

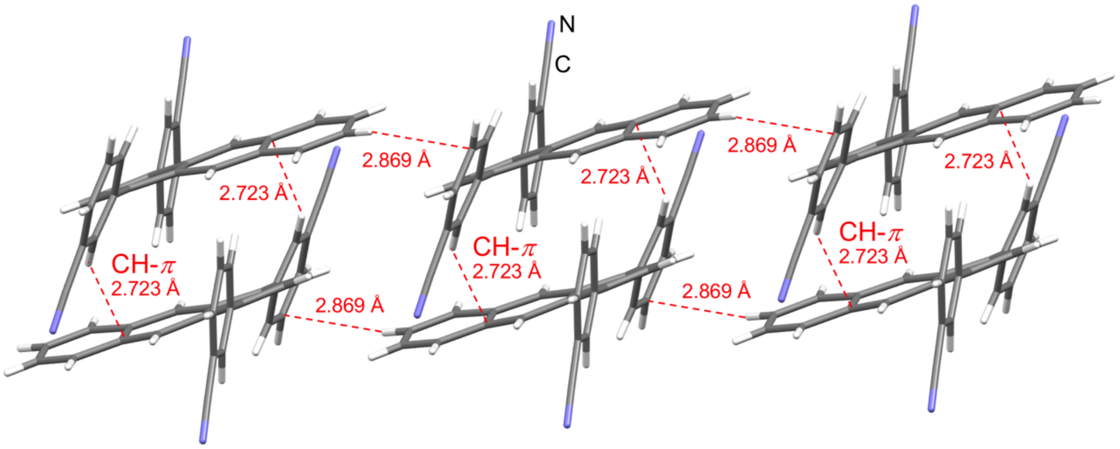

Figure S35. Crystal packing of **2** (3-CN).

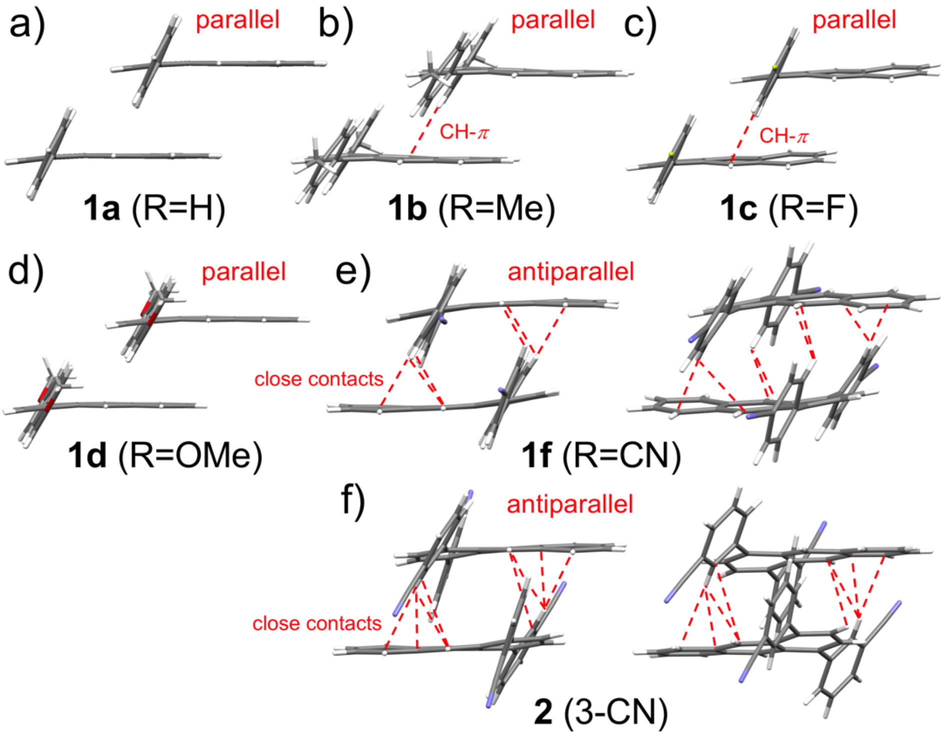

Figure S36. Crystal packing of 1,4-diphenylanthracene derivatives showing bimolecular interactions, a) **1a** (R=H), b) **1b** (R=Me), c) **1c** (R=F), d) **1d** (R=OMe), e) **1f** (R=CN), f) **2** (3-CN).

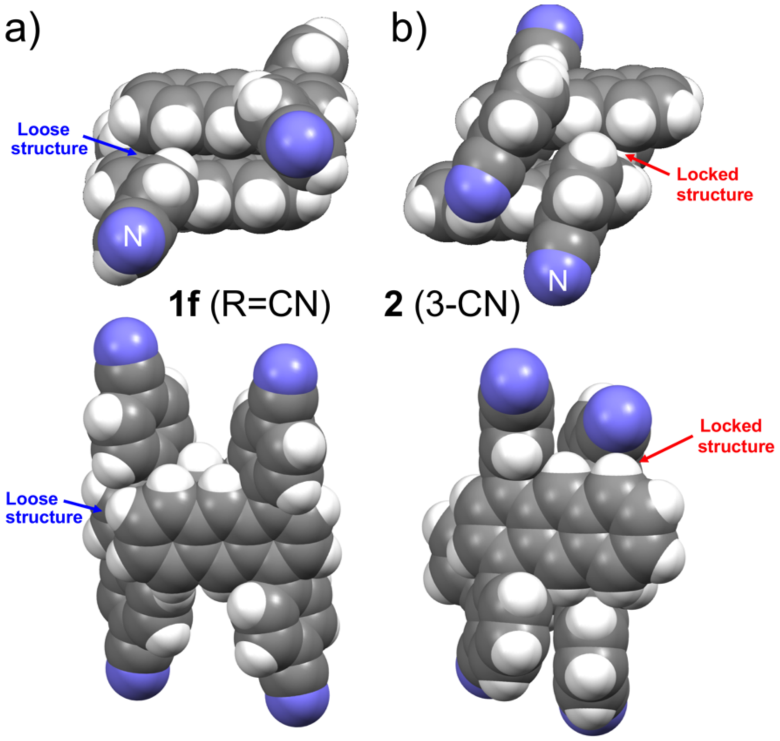

Figure S37. Crystal packing structure of 1,4-diphenylanthracene derivatives showing bimolecular interactions, a) **1f** (R=CN), b) **2** (3-CN).

2.2. Powder X-ray diffraction (PXRD) pattern of 1,4-diphenylanthracence derivatives

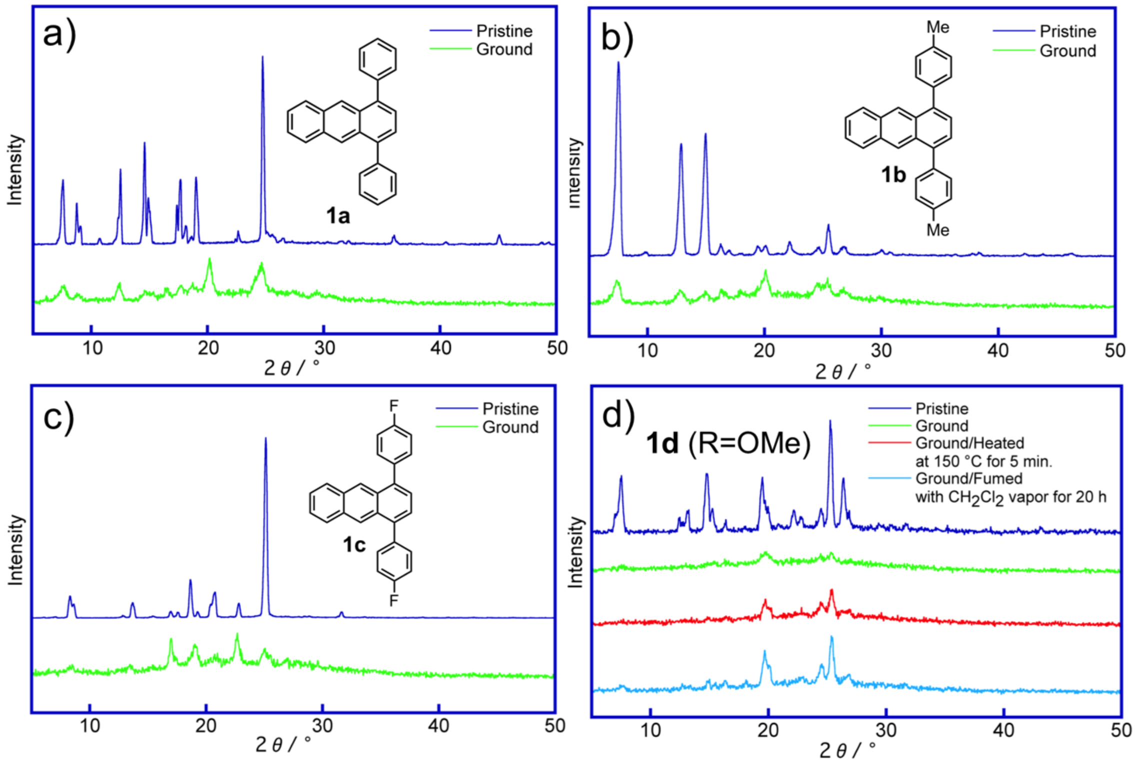

Figure S38. Powder X-ray diffraction (PXRD) patterns of 1,4-diphenylanthracene derivatives in pristine, ground, ground-fumed and ground-heated state, a) **1a** (R=H), b) **1b** (R=Me), c) **1c** (R=F), d) **1d** (R=OMe).

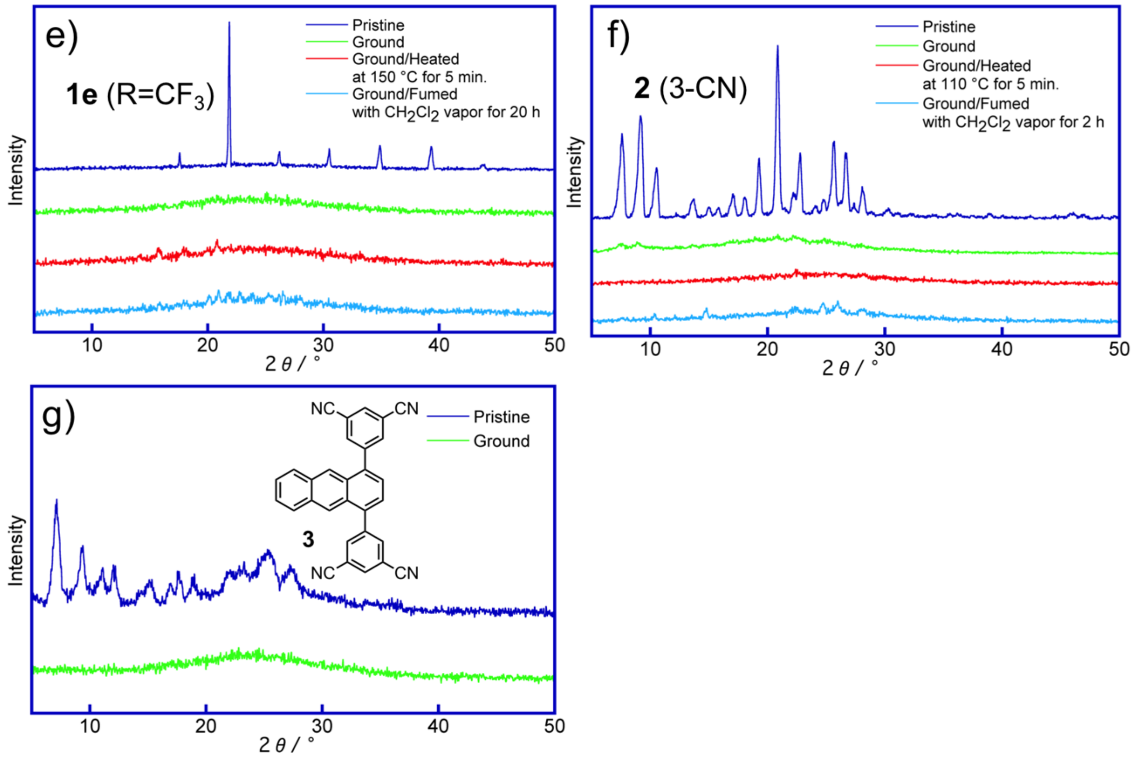

Figure S38 (contd.). Powder X-ray diffraction (PXRD) patterns of 1,4-diphenylanthracene derivatives in pristine, ground, ground-fumed and ground-heated state, e) **1e** (R= CF_3), f) **2** (3-CN), g) **3** (3,5-CN_2, R'=CN).

3. DFT calculation of the obtained crystal structures

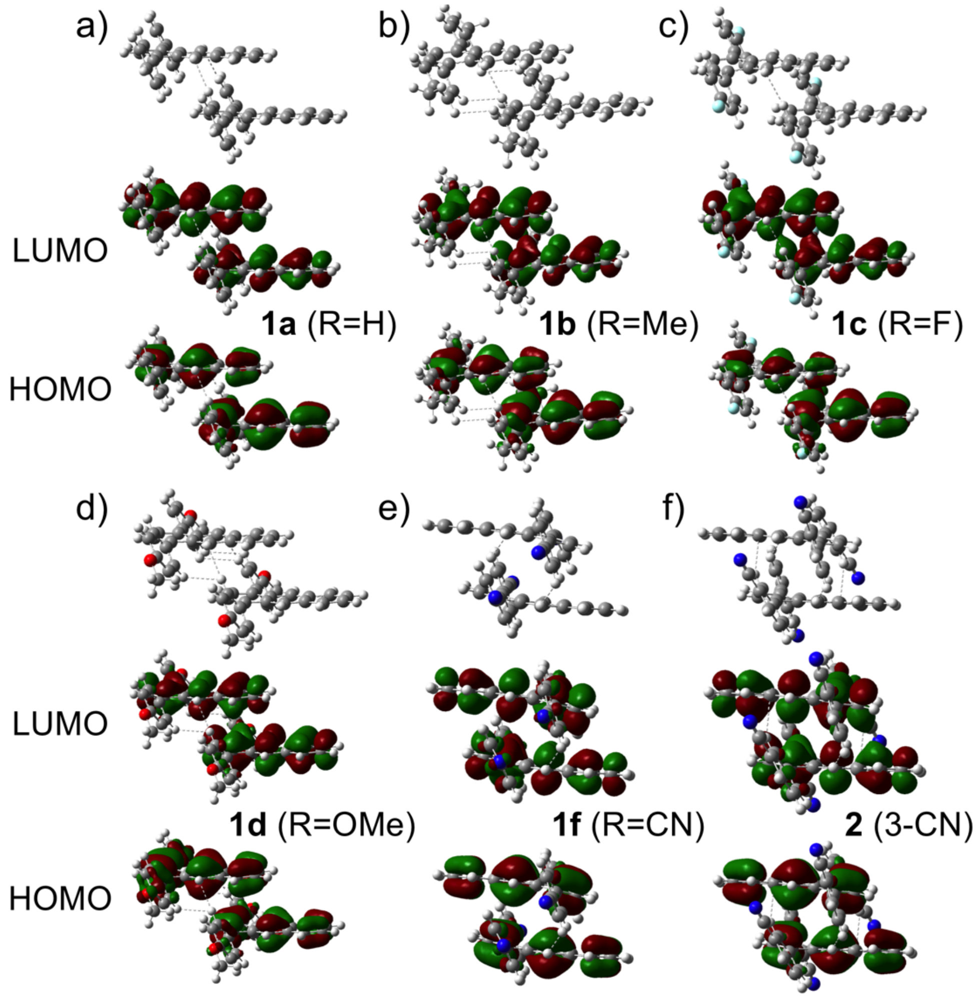

Figure S39. Electron cloud distribution of HOMO and LUMO from crystal structures (2 molecules) of 1,4-diphenylanthracene derivatives calculated by B3LYP-D3/6-31G*, a) **1a** (R=H), b) **1b** (R=Me), c) **1c** (R=F), d) **1d** (R=OMe), e) **1f** (R=CN), f) **2** (3-CN).

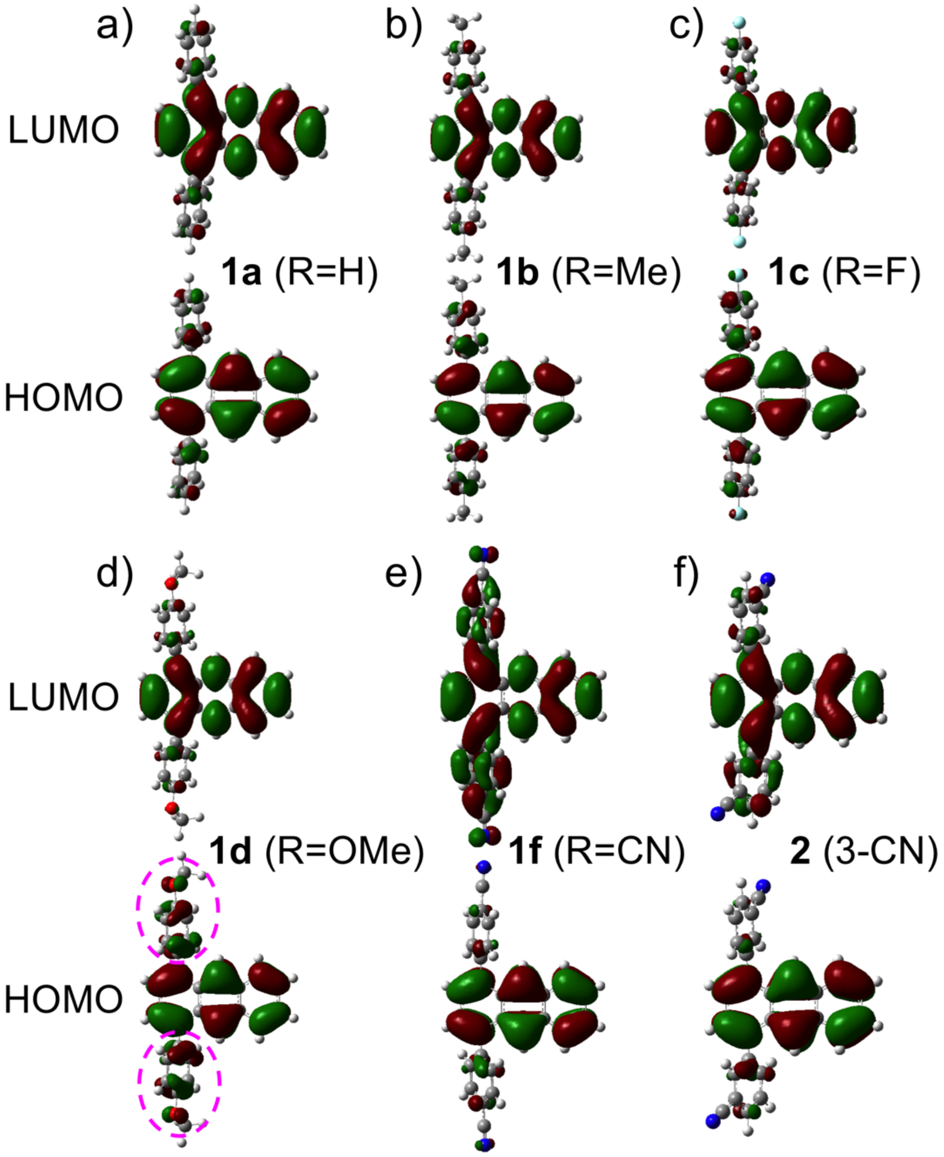

Figure S40. Electron cloud distribution of HOMO and LUMO from crystal structures (1 molecule) of 1,4-diphenylanthracene derivatives calculated by B3LYP-D3/6-31G*, a) **1a** (R=H), b) **1b** (R=Me), c) **1c** (R=F), d) **1d** (R=OMe), e) **1f** (R=CN), f) **2** (3-CN).

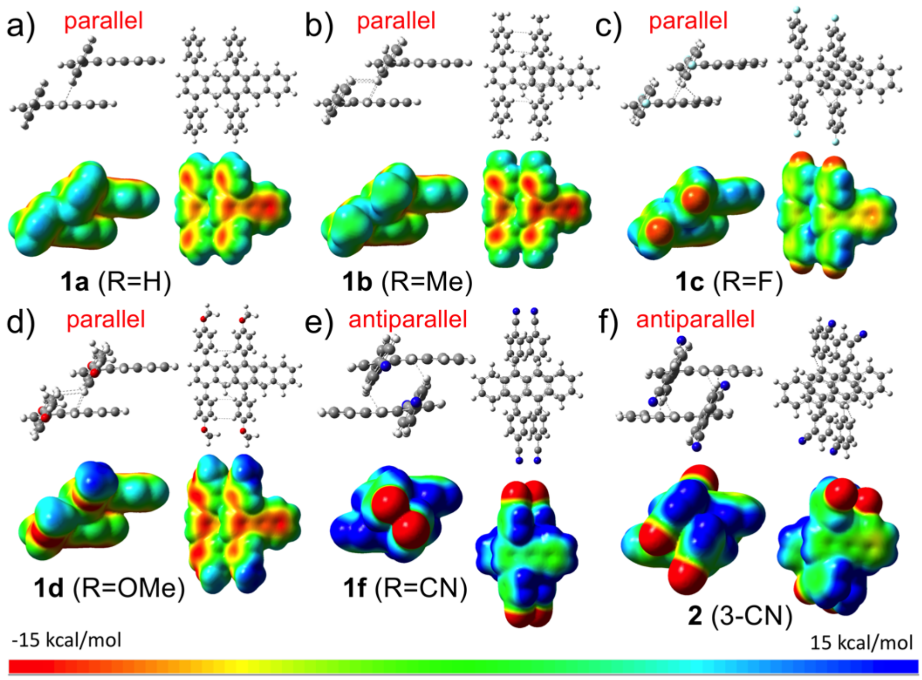

Figure S41. Electrostatic potential maps of 1,4-diphenylanthracene derivatives (bimolecular packing structures) calculated using B3LYP-D3/6-31G* from the obtained crystal data, a) **1a** (R=H), b) **1b** (R=Me), c) **1c** (R=F), d) **1d** (R=OMe), e) **1f** (R=CN), f) **2** (3-CN).

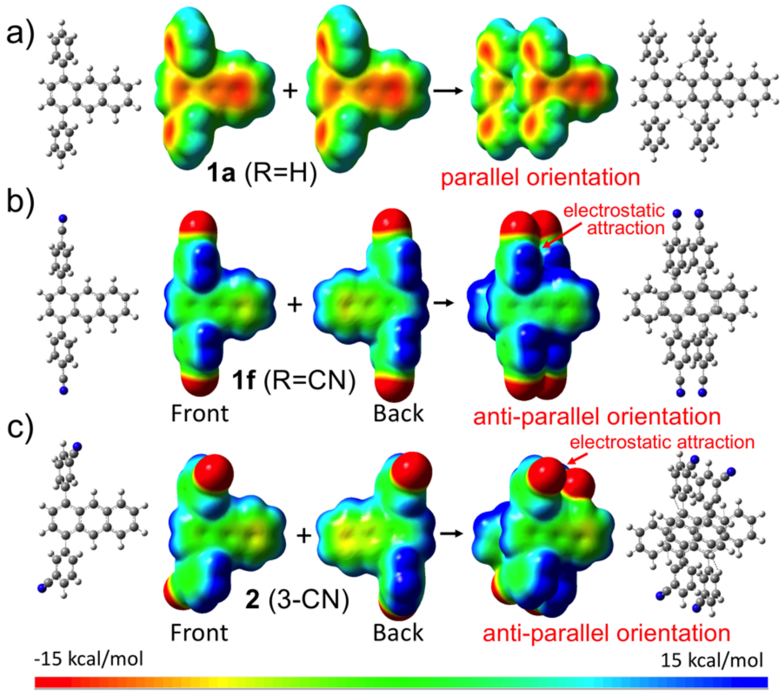

Figure S42. Schematic representation of the electrostatic potential maps for the bimolecular packing structure formation of 1,4-diphenylanthracene derivatives calculated using B3LYP-D3/6-31G* from the obtained crystal data, a) **1a** (R=H), b) **1f** (R=CN), c) **2** (3-CN).

4. UV irradiation experiments for 1,4-diphenylanthracene derivatives

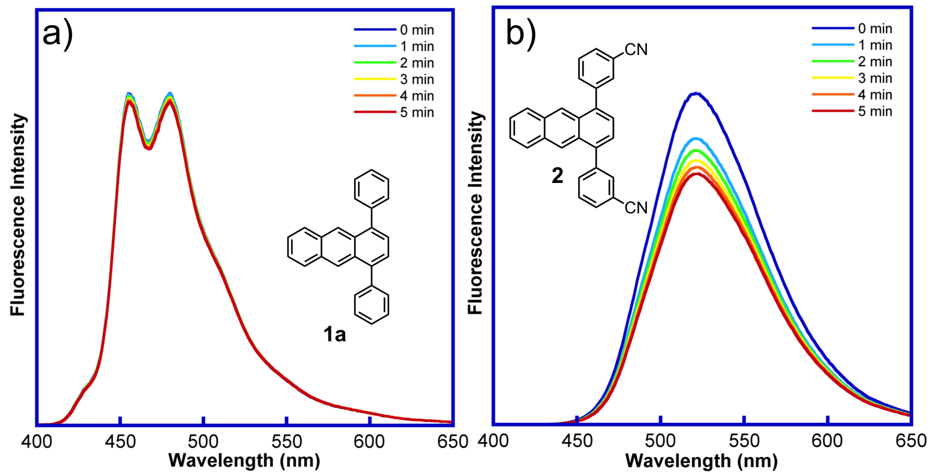

Figure S43. Fluorescence spectra of 1,4-diphenylanthracene derivatives after 365 nm UV irradiation in the solid state (λ_{ex} =365 nm), a) **1a** (R=H), b) **2** (3-CN).

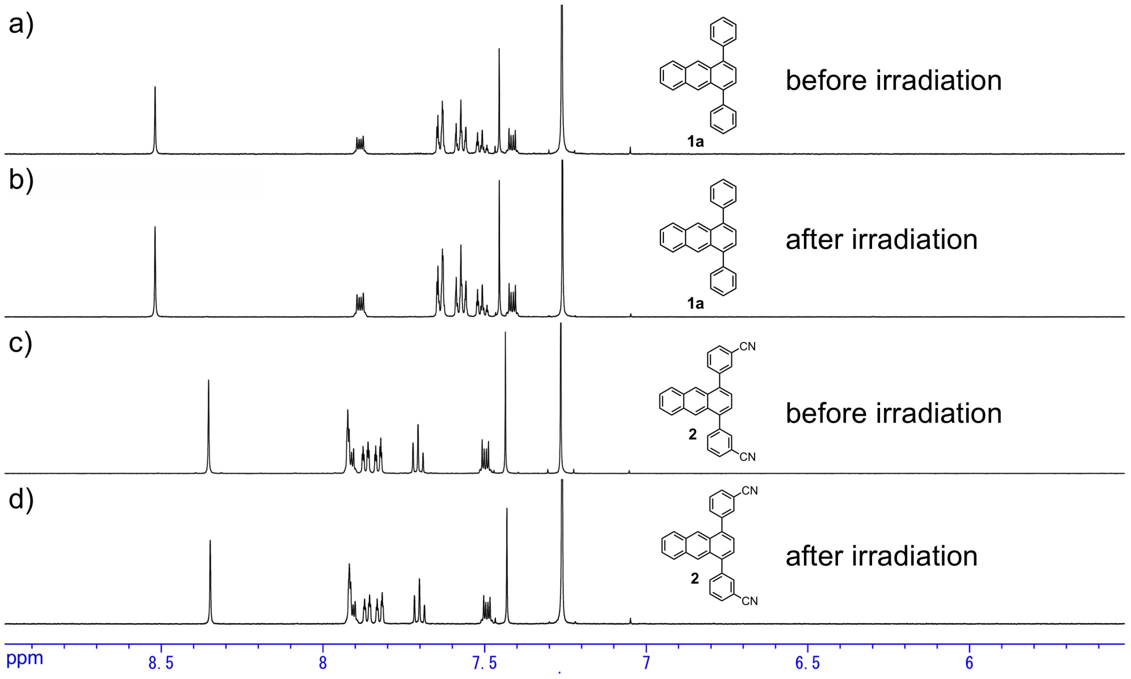

Figure S44. ^{1}H NMR spectra ($CDCl_3$) of 1,4-diphenylanthracene derivatives before and after 365 nm UV irradiation (5 min) in the solid state (λ_{ex} =365 nm), a) **1a** (R=H, before irradiation), b) **1a** (R=H, after irradiation of 365 nm UV light, 5 min), c) **2** (3-CN, before irradiation), d) **2** (3-CN, after irradiation of 365 nm UV light, 5 min).

5. NMR spectra of new compounds

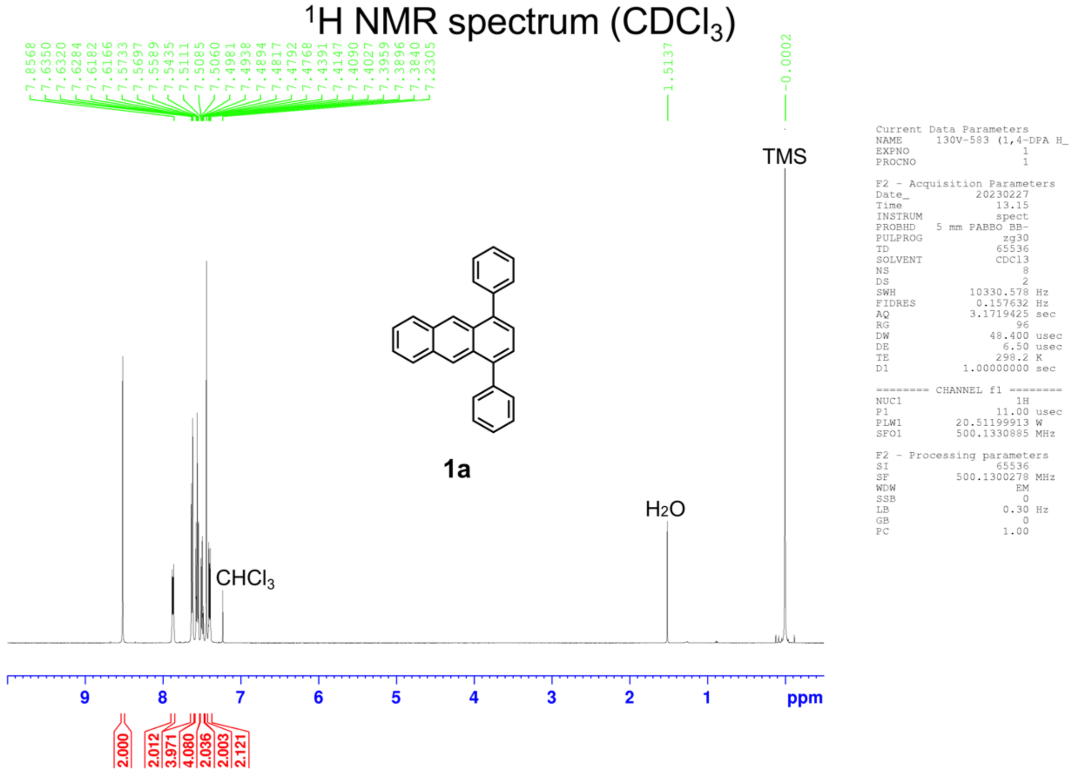

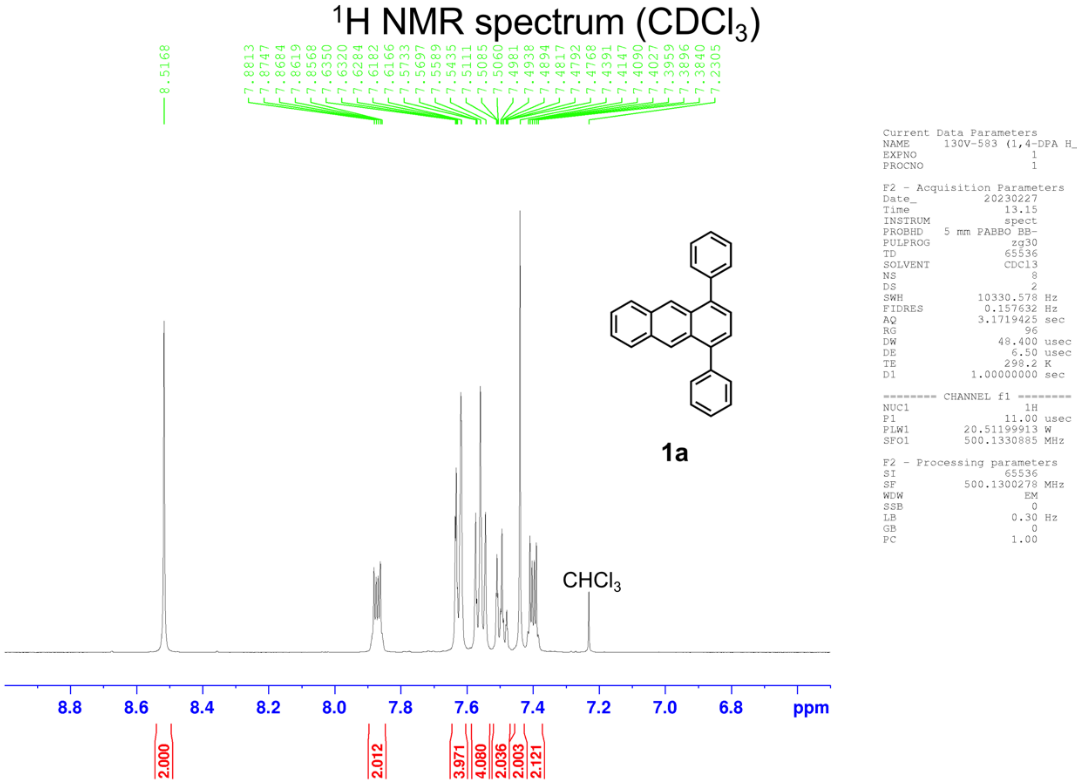

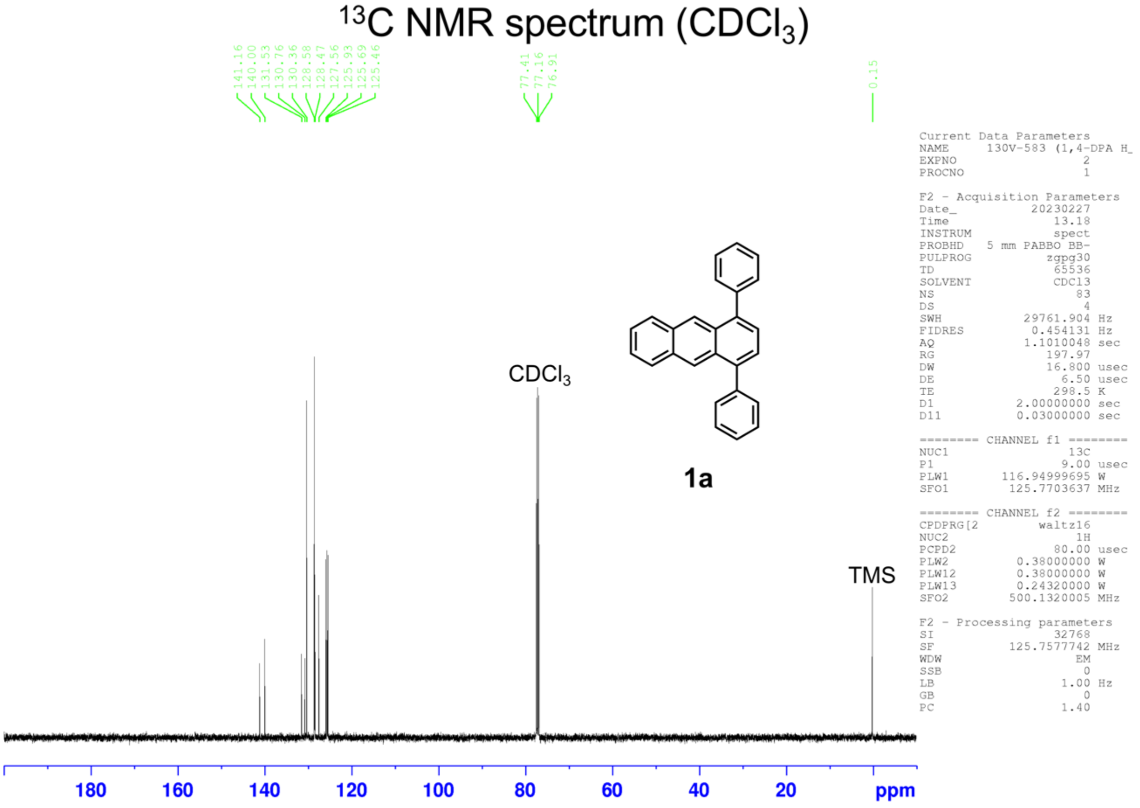

^{13}C NMR spectrum (CDCl$_3$)
141.16
140.00
131.53
130.76
130.36
128.58
128.47
127.56
125.93
125.69
125.46
77.41
77.16
76.91
0.15
CDCl$_3$
TMS
1a
180 160 140 120 100 80 60 40 20 ppm
Current Data Parameters
NAME 130V-583 (1,4-DPA H_
EXPNO 2
PROCNO 1
F2 - Acquisition Parameters
Date_ 20230227
Time 13.18
INSTRUM spect
PROBHD 5 mm PABBO BB-
PULPROG zgpg30
TD 65536
SOLVENT CDCl3
NS 83
DS 4
SWH 29761.904 Hz
FIDRES 0.454131 Hz
AQ 1.1010048 sec
RG 197.97
DW 16.800 usec
DE 6.50 usec
TE 298.5 K
D1 2.00000000 sec
D11 0.03000000 sec
======== CHANNEL f1 ========
NUC1 13C
P1 9.00 usec
PLW1 116.94999695 W
SFO1 125.7703637 MHz
======== CHANNEL f2 ========
CPDPRG[2 waltz16
NUC2 1H
PCPD2 80.00 usec
PLW2 0.38000000 W
PLW12 0.38000000 W
PLW13 0.24320000 W
SFO2 500.1320005 MHz
F2 - Processing parameters
SI 32768
SF 125.7577742 MHz
WDW EM
SSB 0
LB 1.00 Hz
GB 0
PC 1.40

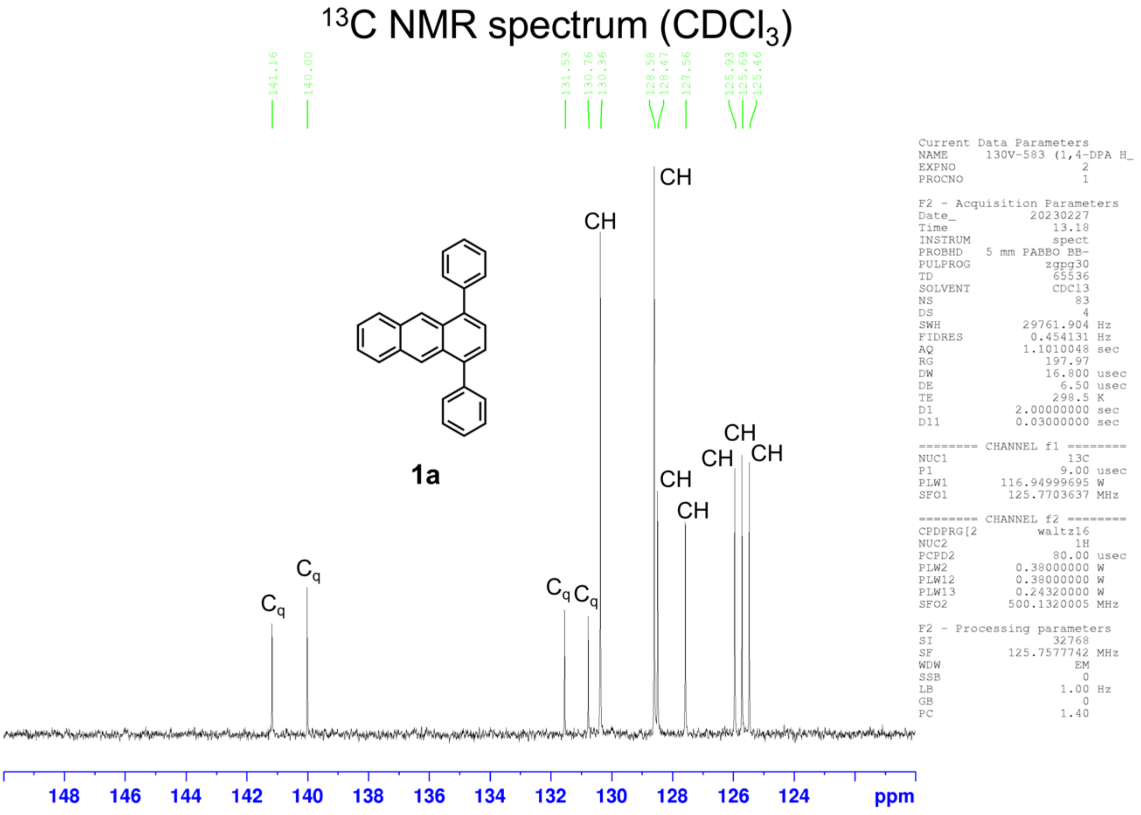

^{13}C NMR spectrum (CDCl$_3$)
141.16
140.00
131.53
130.76
130.36
128.58
128.47
127.56
125.93
125.69
125.46
1a
C$_q$
C$_q$
C$_q$
C$_q$
CH
CH
CH
CH
CH
CH
CH
148 146 144 142 140 138 136 134 132 130 128 126 124 ppm

DEPT 135 NMR spectrum ($CDCl_3$)

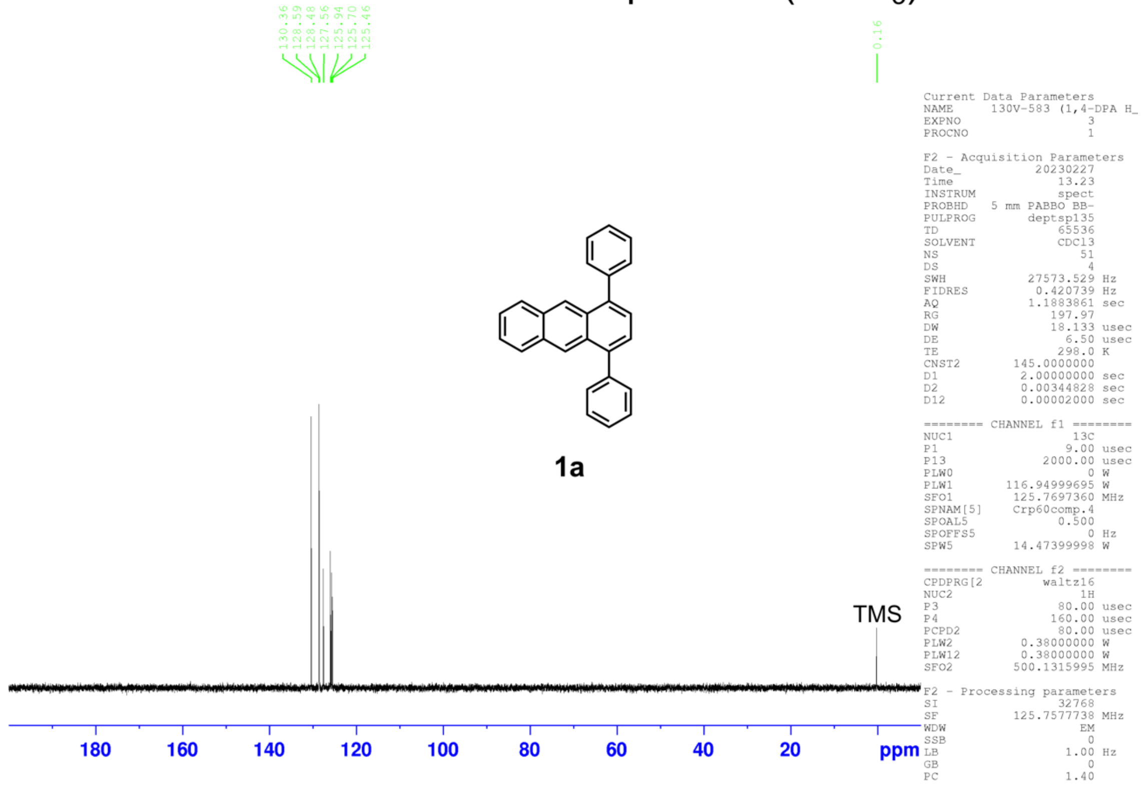

DEPT 135 NMR spectrum ($CDCl_3$)

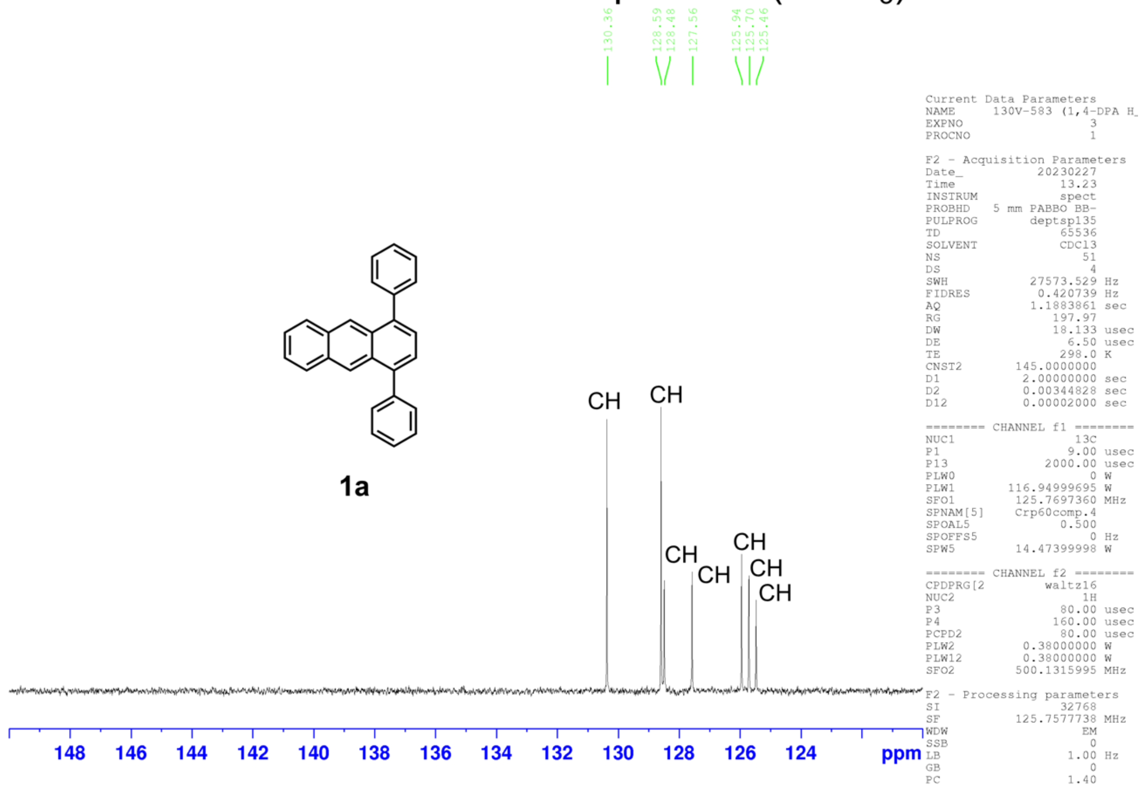

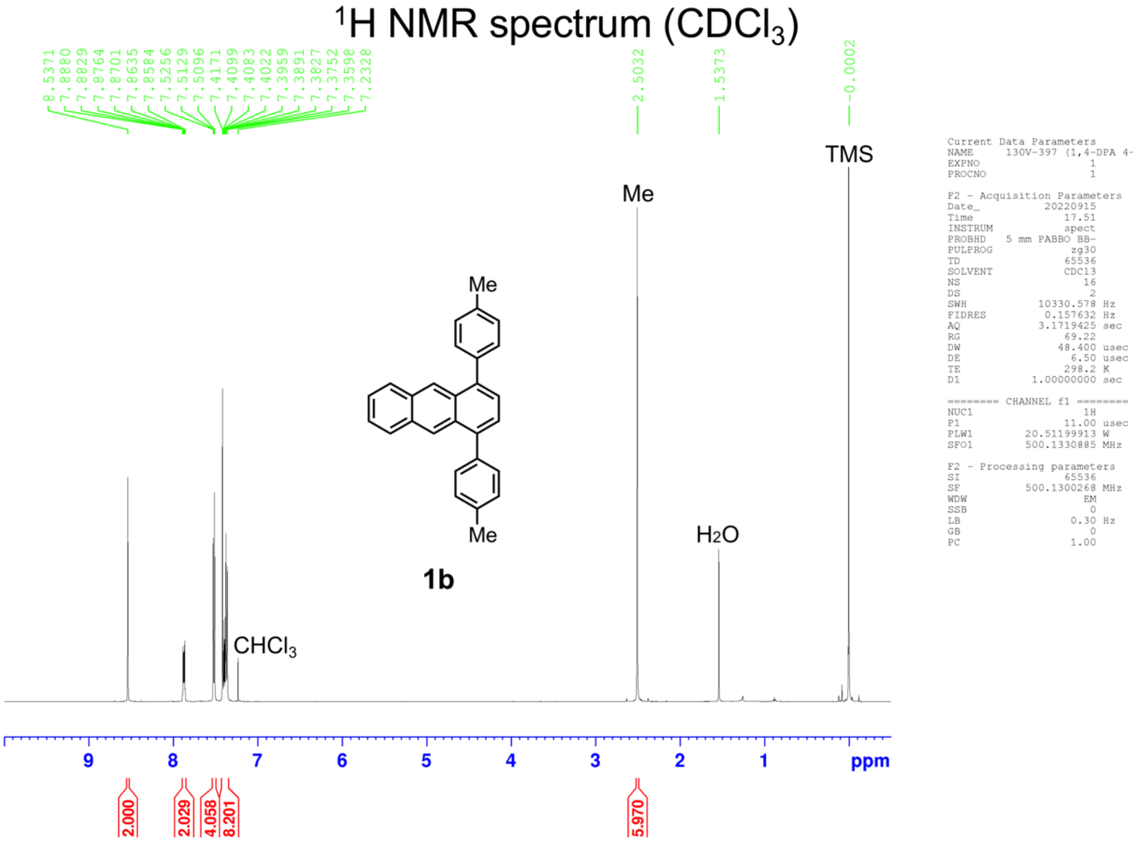

1H NMR spectrum (CDCl3)
8.5371
7.8880
7.8829
7.8764
7.8701
7.8635
7.8584
7.5256
7.5129
7.5096
7.4171
7.4099
7.4083
7.4022
7.3959
7.3891
7.3827
7.3752
7.3598
7.2328
2.5032
1.5373
-0.0002
TMS
Me
Me
Me
H2O
CHCl3
1b
9
8
7
6
5
4
3
2
1
ppm
2.000
2.029
4.058
8.201
5.970
Current Data Parameters
NAME 130V-397 (1,4-DPA 4-
EXPNO 1
PROCNO 1
F2 - Acquisition Parameters
Date_ 20220915
Time 17.51
INSTRUM spect
PROBHD 5 mm PABBO BB-
PULPROG zg30
TD 65536
SOLVENT CDCl3
NS 16
DS 2
SWH 10330.578 Hz
FIDRES 0.157632 Hz
AQ 3.1719425 sec
RG 69.22
DW 48.400 usec
DE 6.50 usec
TE 298.2 K
D1 1.00000000 sec
======== CHANNEL f1 ========
NUC1 1H
P1 11.00 usec
PLW1 20.51199913 W
SFO1 500.1330885 MHz
F2 - Processing parameters
SI 65536
SF 500.1300268 MHz
WDW EM
SSB 0
LB 0.30 Hz
GB 0
PC 1.00

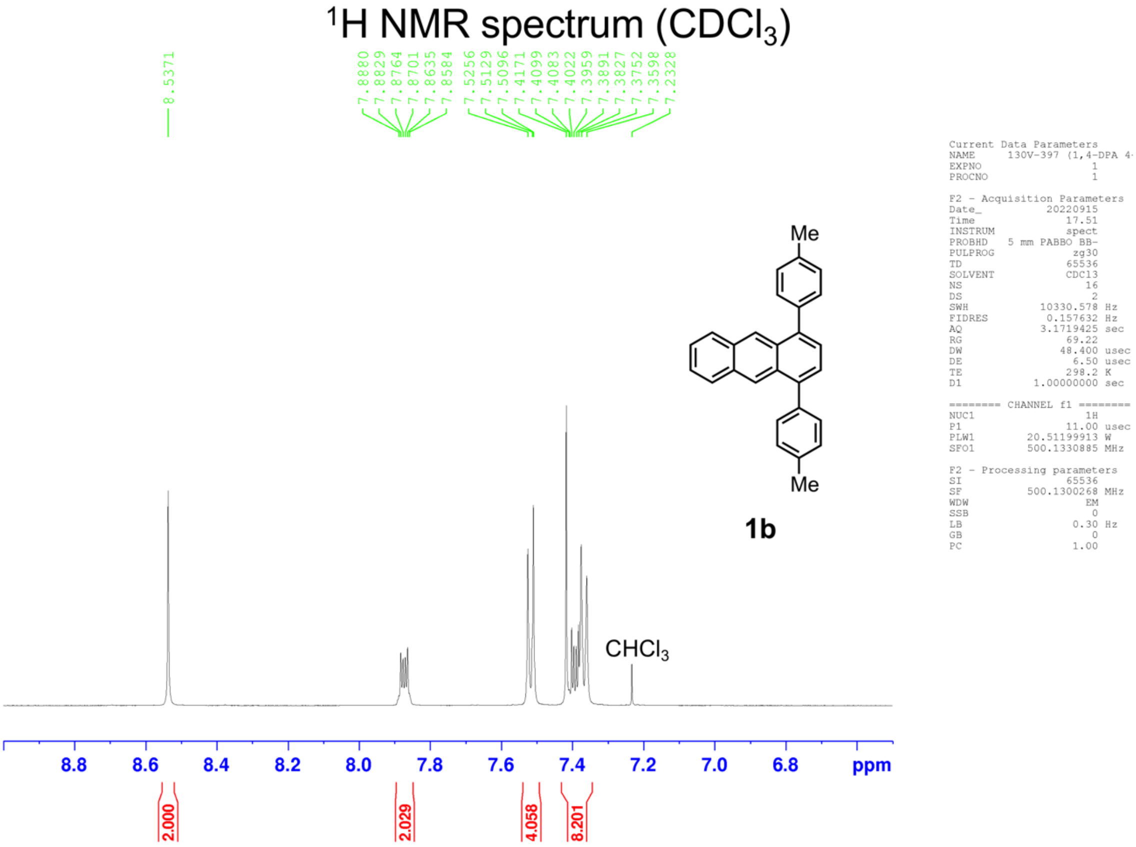

1H NMR spectrum (CDCl3)
8.5371
7.8880
7.8829
7.8764
7.8701
7.8635
7.8584
7.5256
7.5129
7.5096
7.4171
7.4099
7.4083
7.4022
7.3959
7.3891
7.3827
7.3752
7.3598
7.2328
Me
Me
1b
CHCl3
8.8
8.6
8.4
8.2
8.0
7.8
7.6
7.4
7.2
7.0
6.8
ppm
2.000
2.029
4.058
8.201
Current Data Parameters
NAME 130V-397 (1,4-DPA 4-
EXPNO 1
PROCNO 1
F2 - Acquisition Parameters
Date_ 20220915
Time 17.51
INSTRUM spect
PROBHD 5 mm PABBO BB-
PULPROG zg30
TD 65536
SOLVENT CDCl3
NS 16
DS 2
SWH 10330.578 Hz
FIDRES 0.157632 Hz
AQ 3.1719425 sec
RG 69.22
DW 48.400 usec
DE 6.50 usec
TE 298.2 K
D1 1.00000000 sec
======== CHANNEL f1 ========
NUC1 1H
P1 11.00 usec
PLW1 20.51199913 W
SFO1 500.1330885 MHz
F2 - Processing parameters
SI 65536
SF 500.1300268 MHz
WDW EM
SSB 0
LB 0.30 Hz
GB 0
PC 1.00

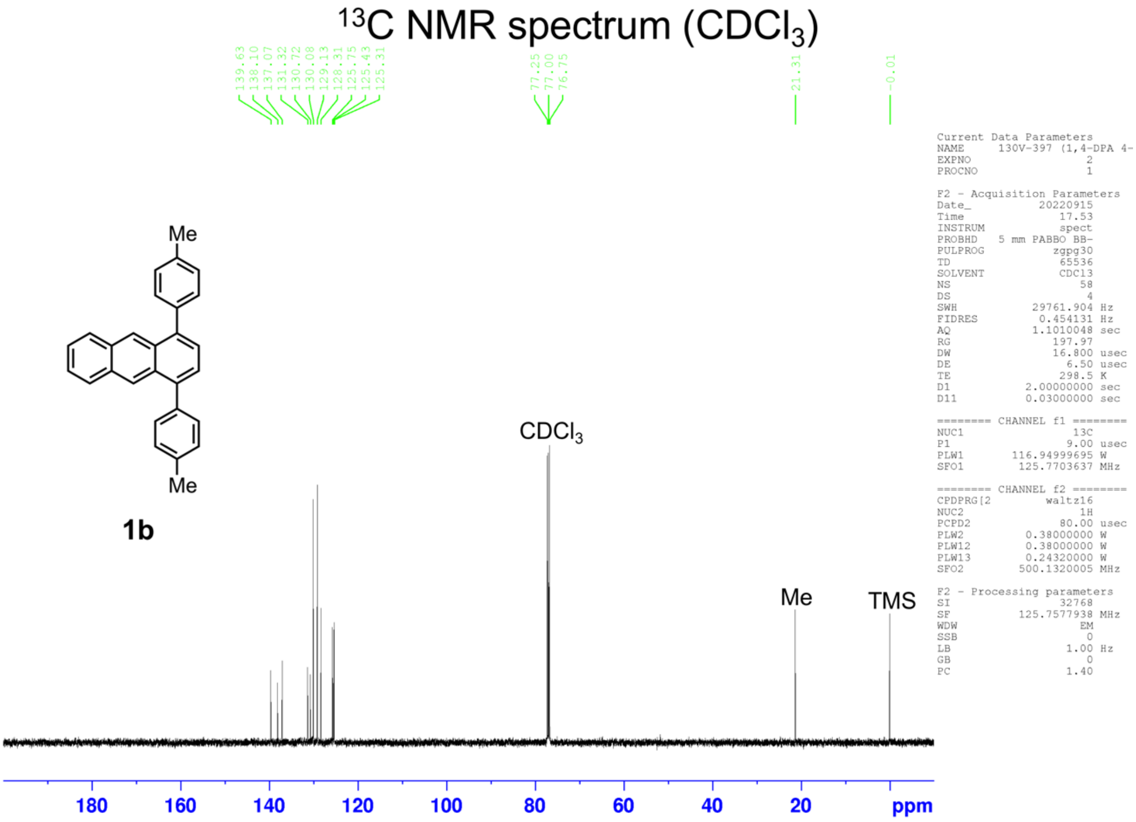
13C NMR spectrum (CDCl3)
139.63
138.10
137.07
131.32
130.72
130.08
129.13
128.31
125.75
125.43
125.31
77.25
77.00
76.75
21.31
-0.01
Me
Me
1b
CDCl3
Me
TMS
180
160
140
120
100
80
60
40
20
ppm

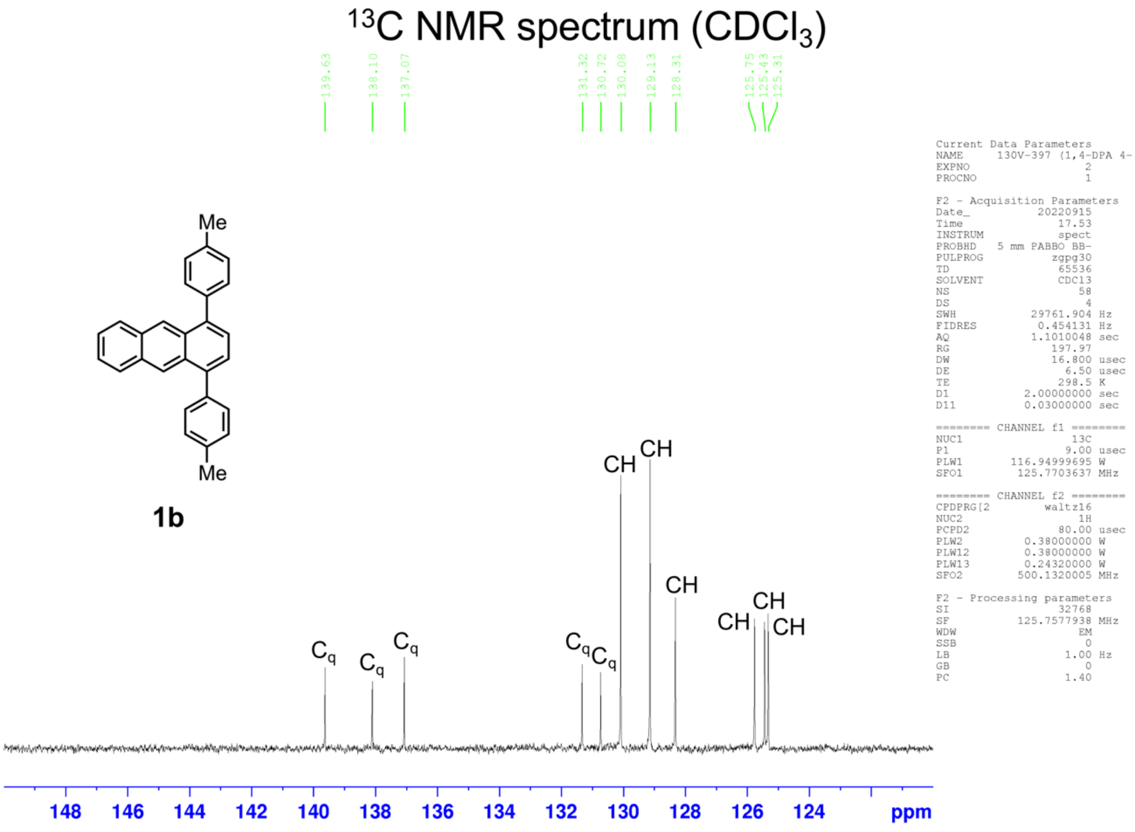
13C NMR spectrum (CDCl3)
139.63
138.10
137.07
131.32
130.72
130.08
129.13
128.31
125.75
125.43
125.31
Me
Me
1b
Cq
Cq
Cq
Cq
Cq
CH
CH
CH
CH
CH
CH
148
146
144
142
140
138
136
134
132
130
128
126
124
ppm

DEPT 135 NMR spectrum ($CDCl_3$)

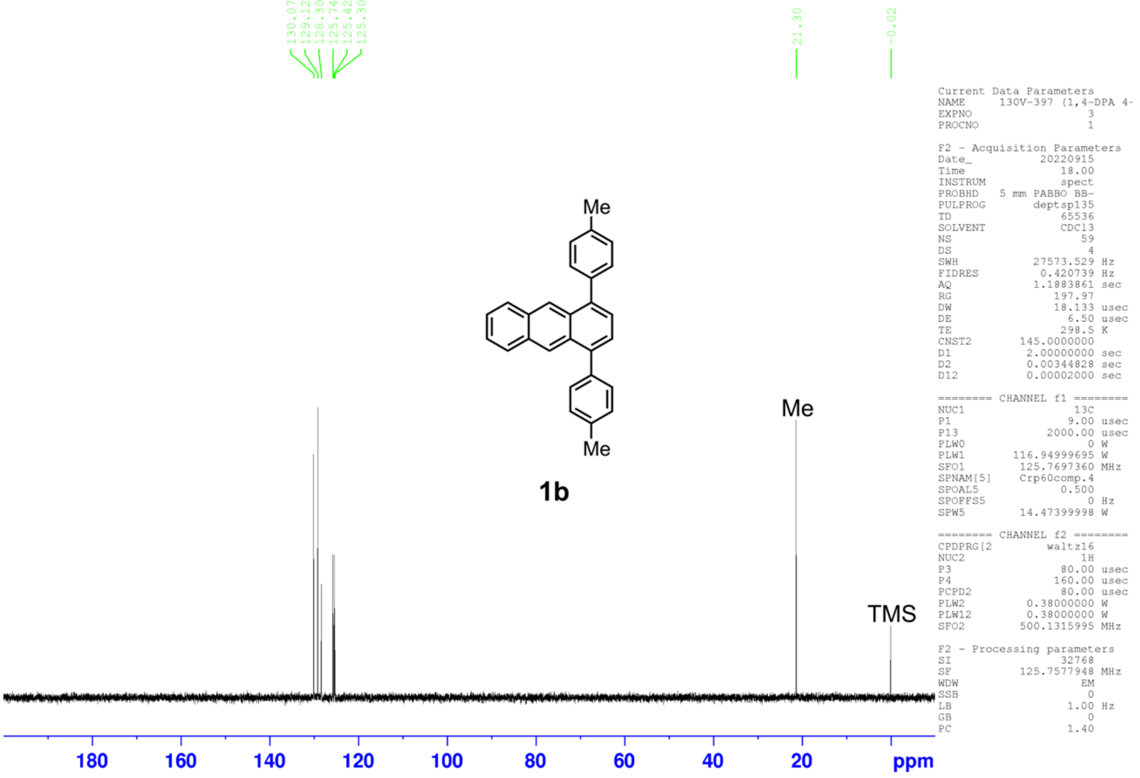

DEPT 135 NMR spectrum ($CDCl_3$)

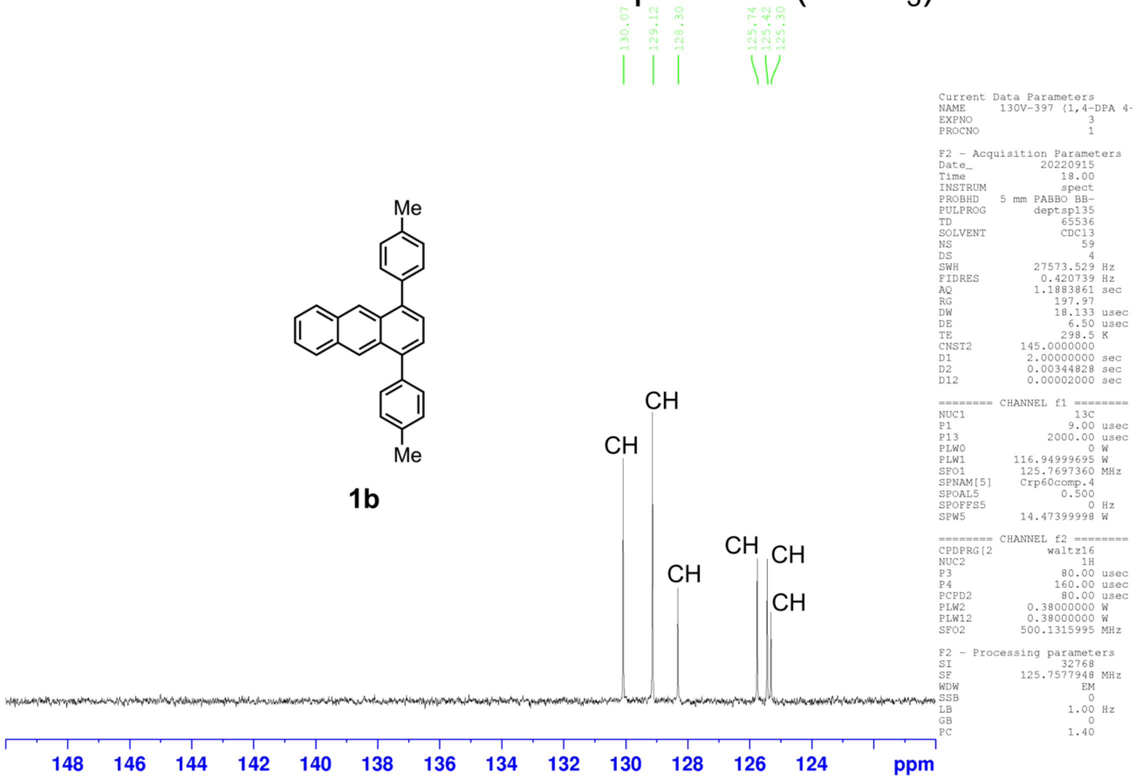

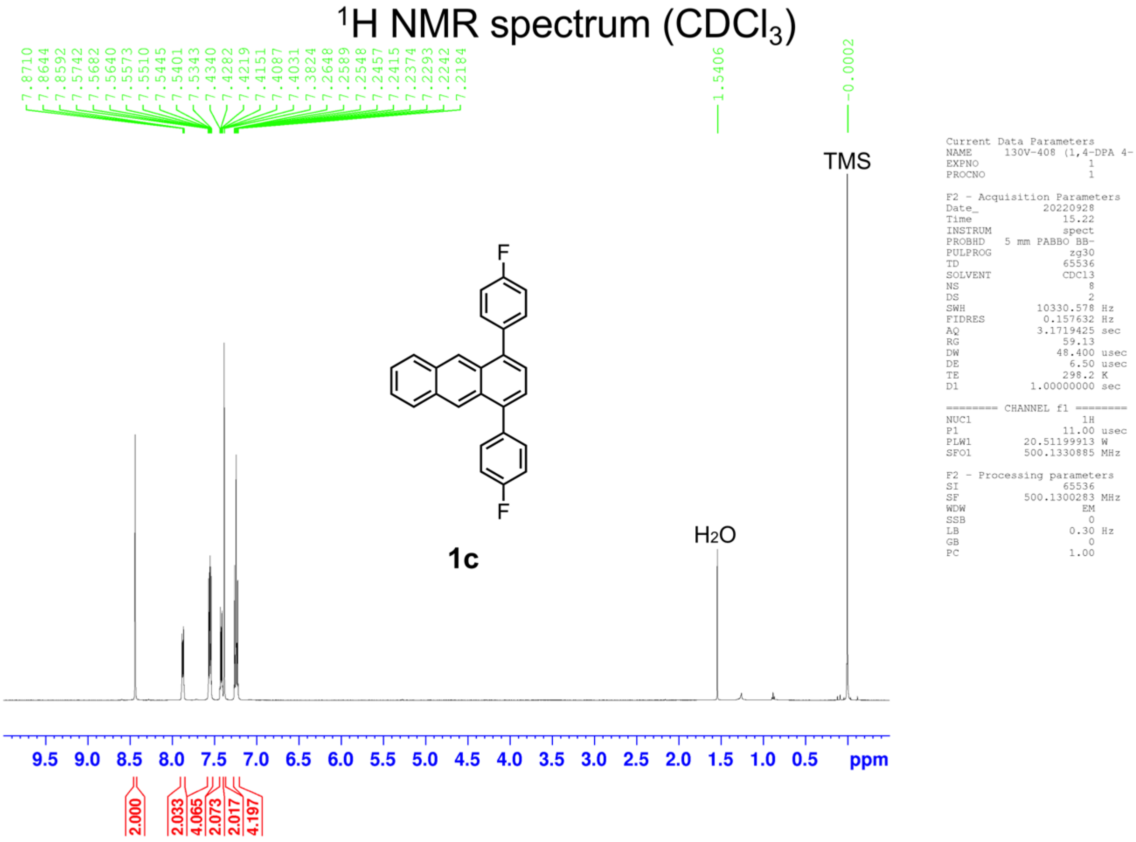
1H NMR spectrum (CDCl3)
7.8710
7.8644
7.8592
7.5742
7.5682
7.5640
7.5573
7.5510
7.5445
7.5401
7.5343
7.4340
7.4282
7.4219
7.4151
7.4087
7.4031
7.3824
7.2648
7.2589
7.2548
7.2457
7.2415
7.2374
7.2293
7.2242
7.2184
1.5406
-0.0002
TMS
F
F
1c
H2O
9.5 9.0 8.5 8.0 7.5 7.0 6.5 6.0 5.5 5.0 4.5 4.0 3.5 3.0 2.5 2.0 1.5 1.0 0.5 ppm
2.000
2.033
4.065
2.073
2.017
4.197
Current Data Parameters
NAME 130V-408 (1,4-DPA 4-
EXPNO 1
PROCNO 1
F2 - Acquisition Parameters
Date_ 20220928
Time 15.22
INSTRUM spect
PROBHD 5 mm PABBO BB-
PULPROG zg30
TD 65536
SOLVENT CDCl3
NS 8
DS 2
SWH 10330.578 Hz
FIDRES 0.157632 Hz
AQ 3.1719425 sec
RG 59.13
DW 48.400 usec
DE 6.50 usec
TE 298.2 K
D1 1.00000000 sec
======== CHANNEL f1 ========
NUC1 1H
P1 11.00 usec
PLW1 20.51199913 W
SFO1 500.1330885 MHz
F2 - Processing parameters
SI 65536
SF 500.1300283 MHz
WDW EM
SSB 0
LB 0.30 Hz
GB 0
PC 1.00

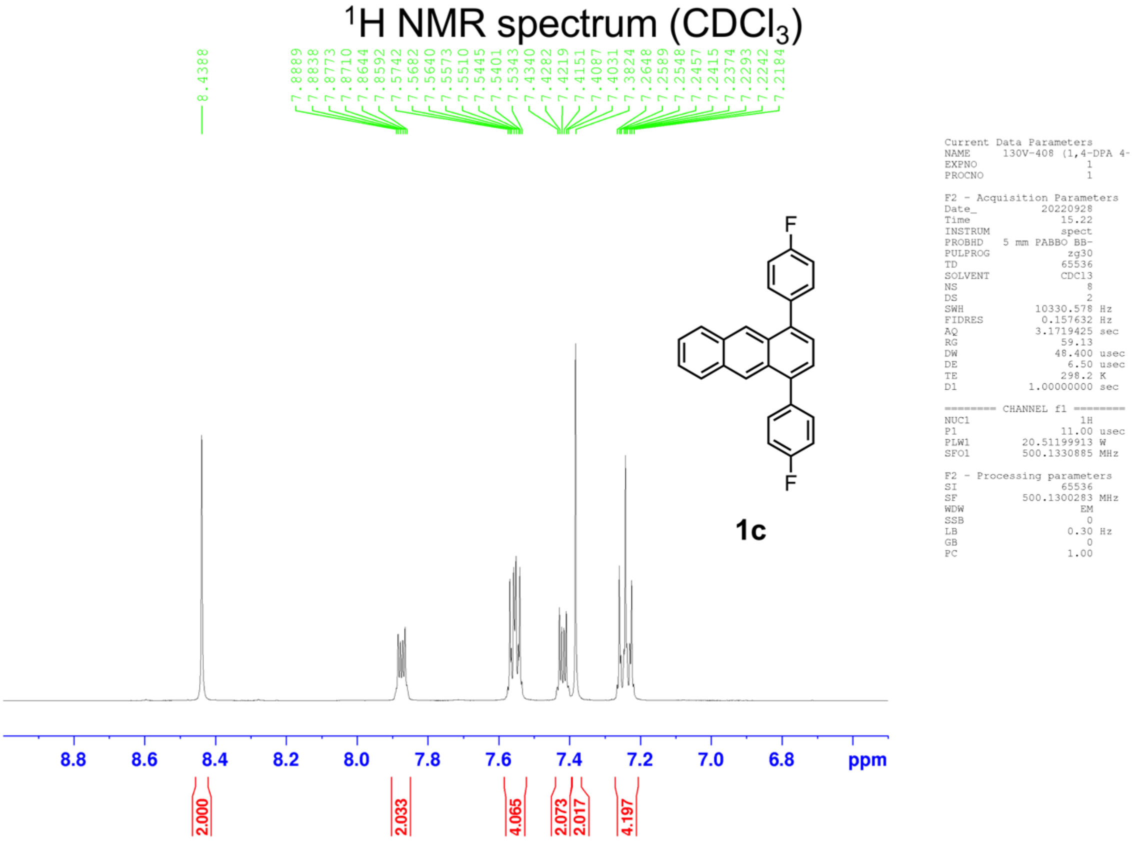
1H NMR spectrum (CDCl3)
8.4388
7.8889
7.8838
7.8773
7.8710
7.8644
7.8592
7.5742
7.5682
7.5640
7.5573
7.5510
7.5445
7.5401
7.5343
7.4340
7.4282
7.4219
7.4151
7.4087
7.4031
7.3824
7.2648
7.2589
7.2548
7.2457
7.2415
7.2374
7.2293
7.2242
7.2184
F
F
1c
8.8 8.6 8.4 8.2 8.0 7.8 7.6 7.4 7.2 7.0 6.8 ppm
2.000
2.033
4.065
2.073
2.017
4.197

^{1}H NMR spectrum ($CDCl_3$)

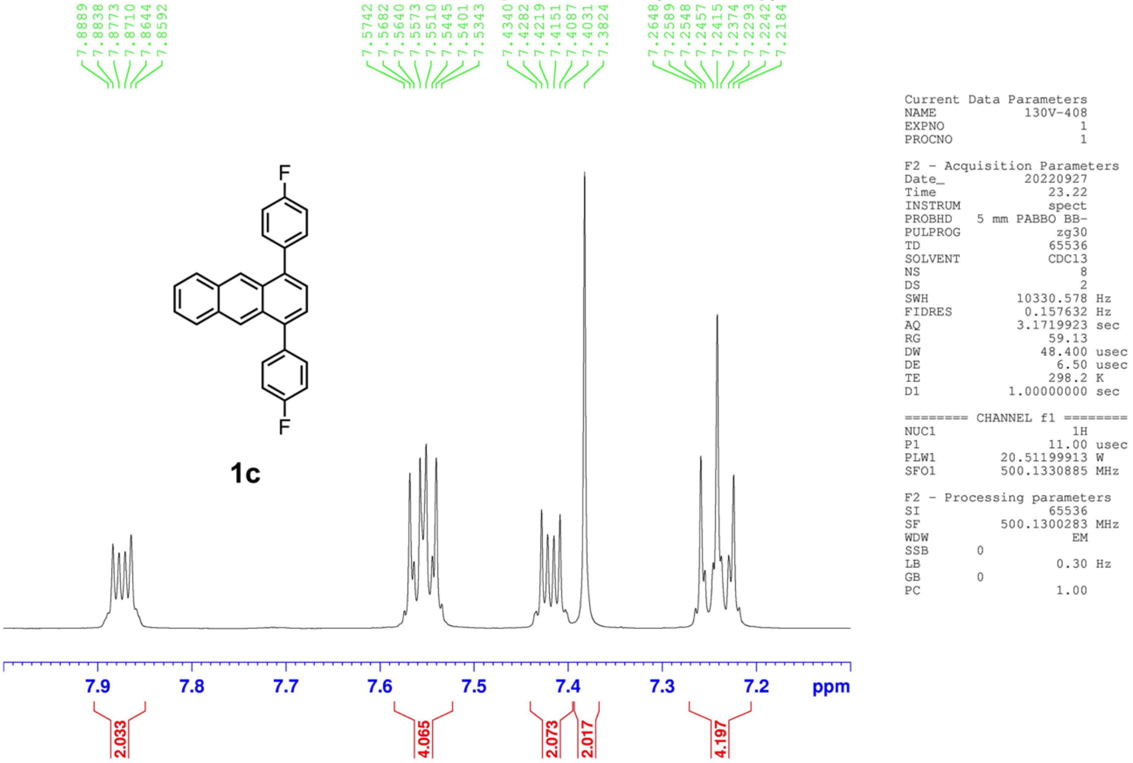

^{13}C NMR spectrum ($CDCl_3$)

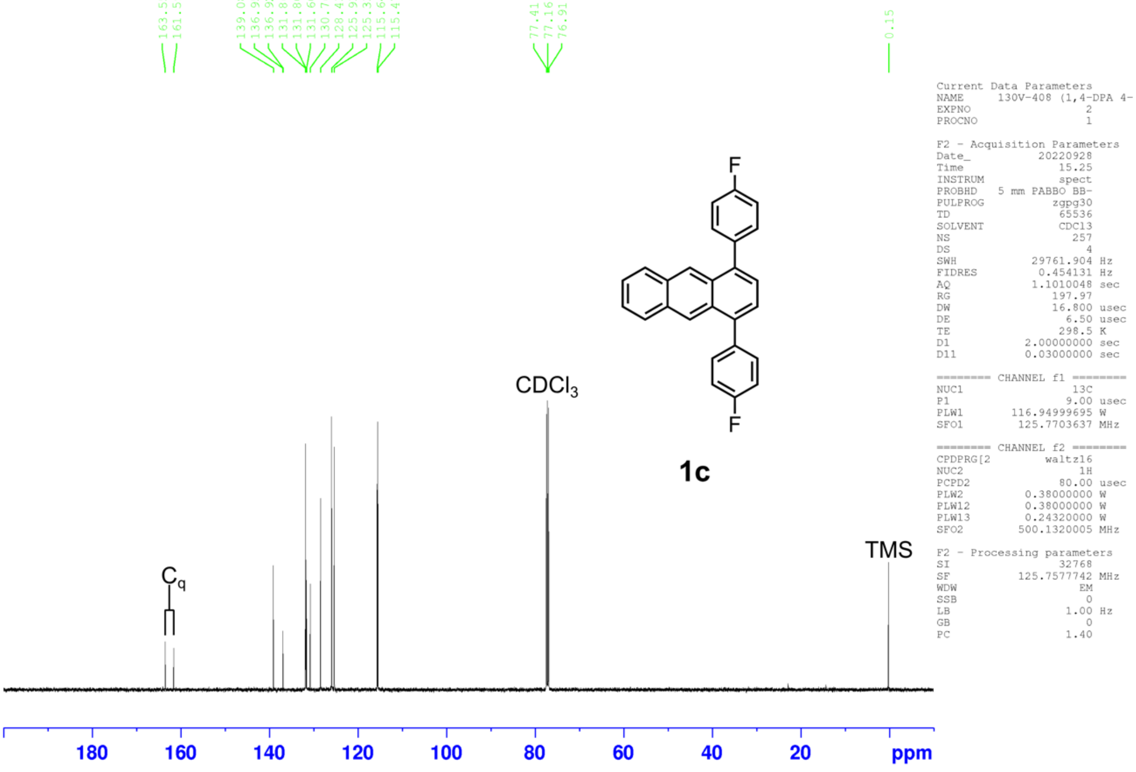

^{13}C NMR spectrum ($CDCl_3$)

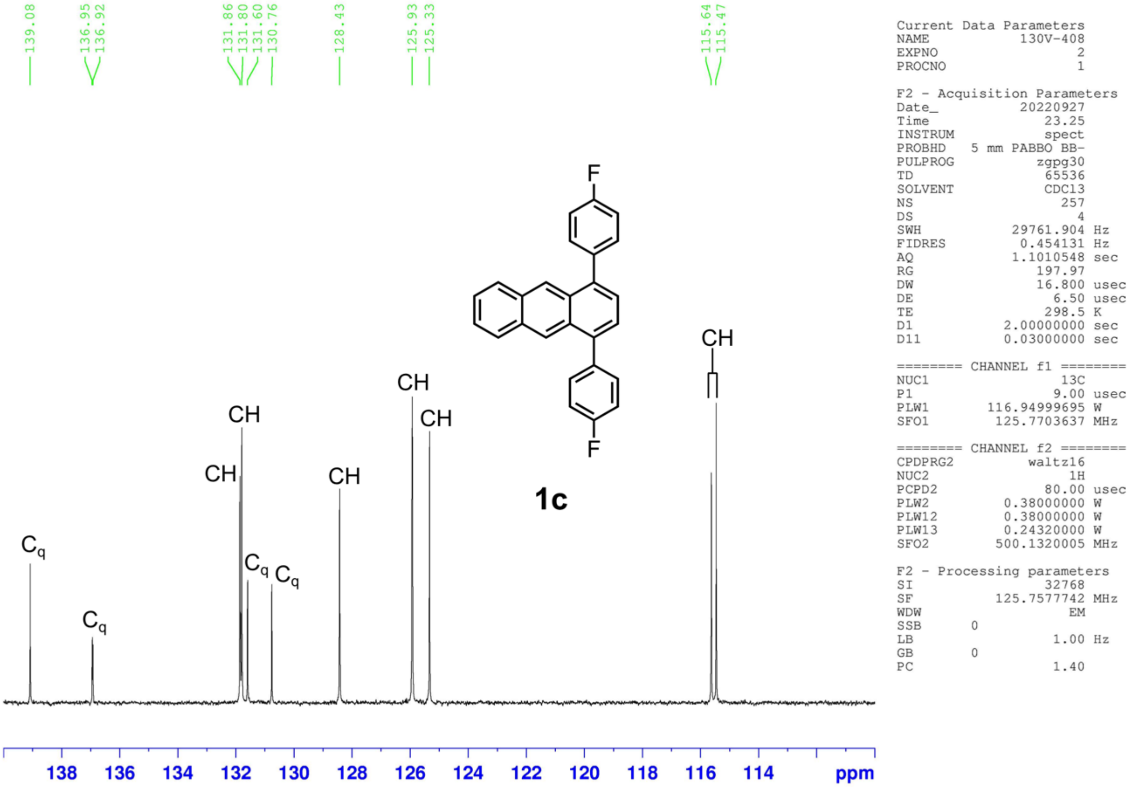

^{13}C NMR spectrum ($CDCl_3$)

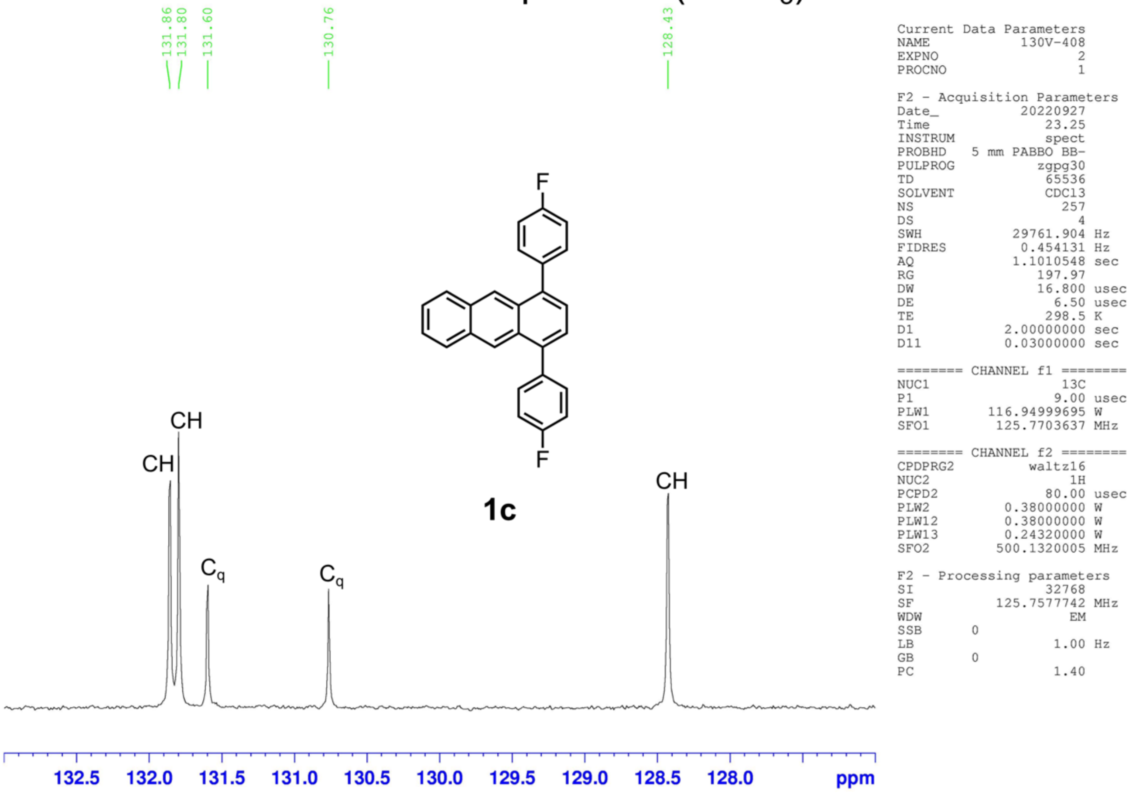

DEPT 135 NMR spectrum ($CDCl_3$)

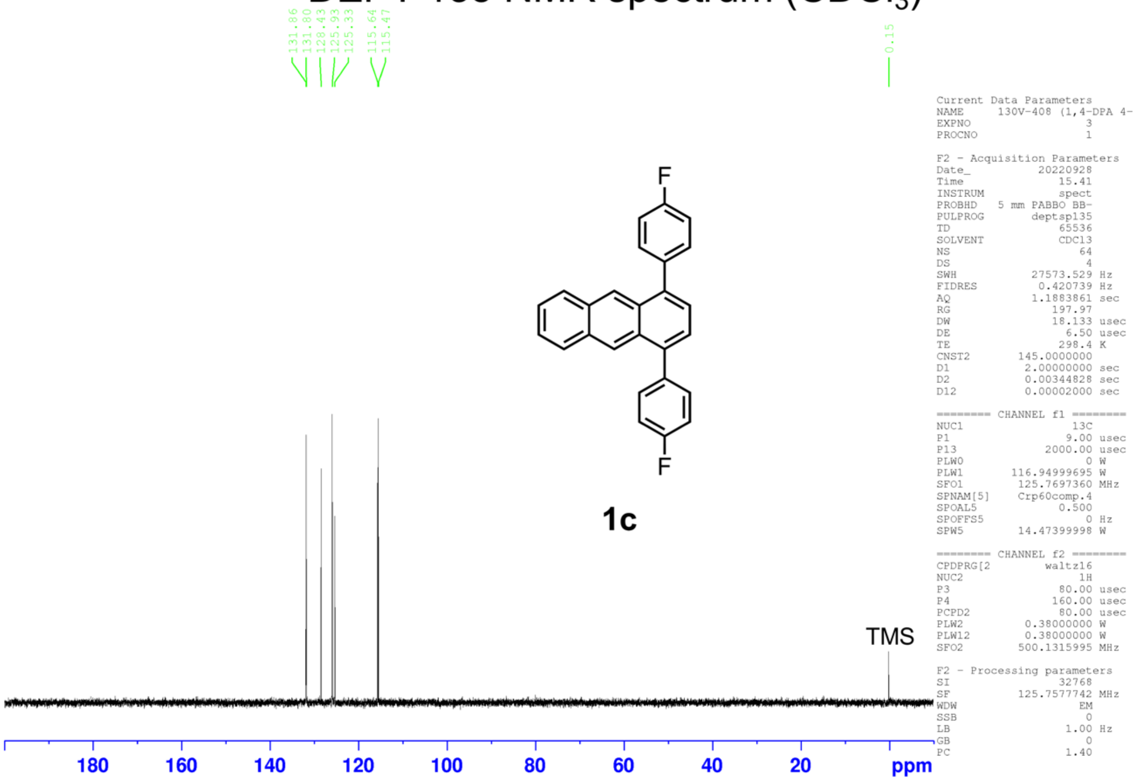

DEPT 135 NMR spectrum ($CDCl_3$)

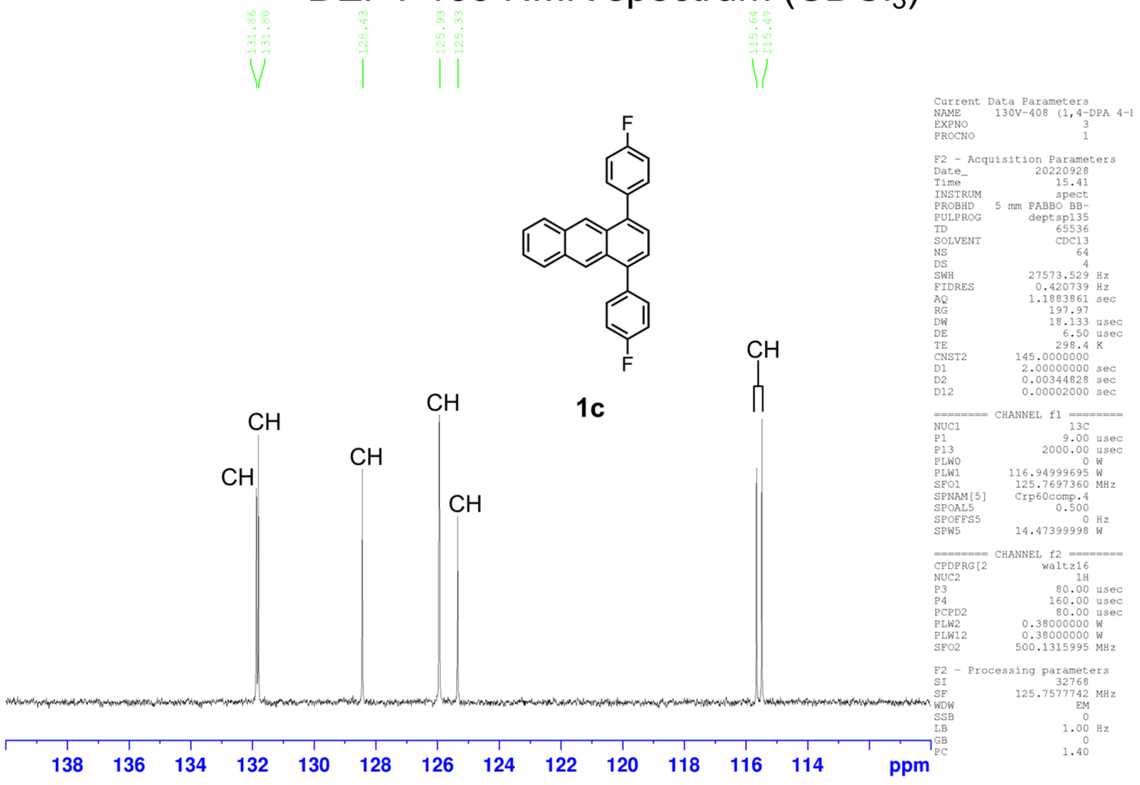

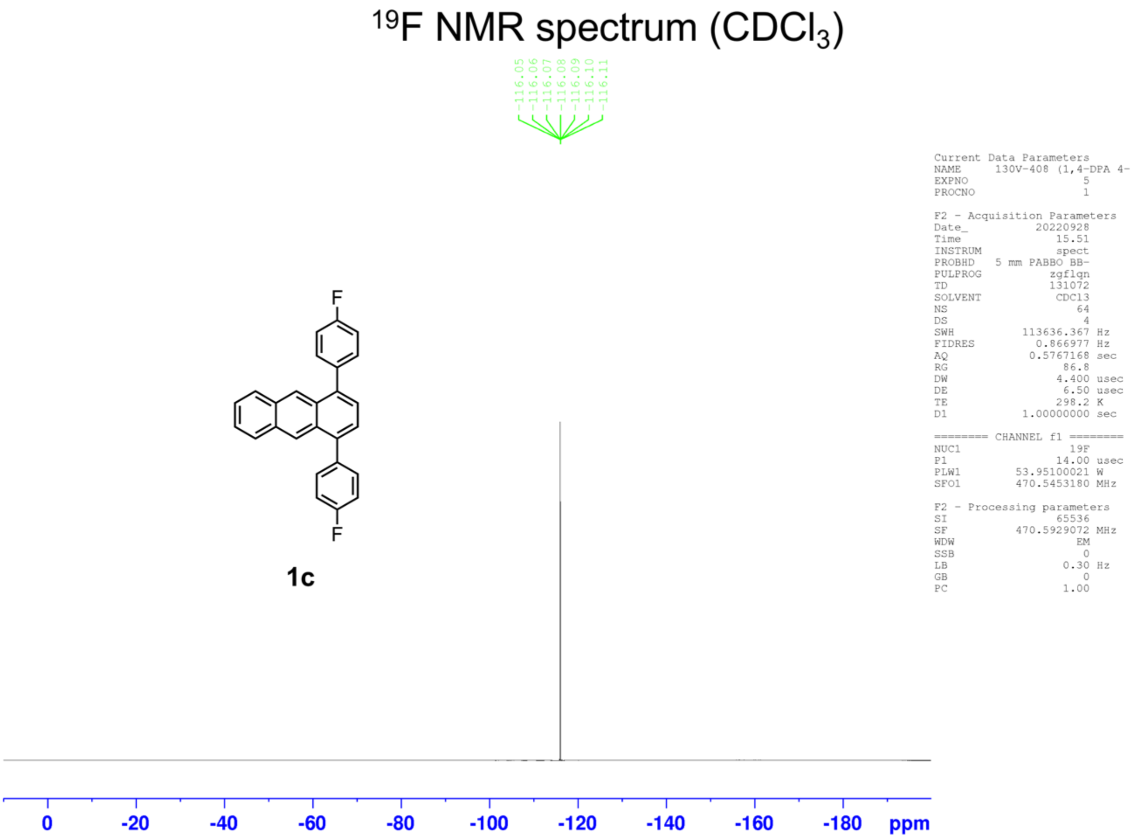
19F NMR spectrum (CDCl3)
-116.05
-116.06
-116.07
-116.08
-116.09
-116.10
-116.11
F
F
1c
Current Data Parameters
NAME 130V-408 (1,4-DPA 4-
EXPNO 5
PROCNO 1
F2 - Acquisition Parameters
Date_ 20220928
Time 15.51
INSTRUM spect
PROBHD 5 mm PABBO BB-
PULPROG zgflqn
TD 131072
SOLVENT CDCl3
NS 64
DS 4
SWH 113636.367 Hz
FIDRES 0.866977 Hz
AQ 0.5767168 sec
RG 86.8
DW 4.400 usec
DE 6.50 usec
TE 298.2 K
D1 1.00000000 sec
======== CHANNEL f1 ========
NUC1 19F
P1 14.00 usec
PLW1 53.95100021 W
SFO1 470.5453180 MHz
F2 - Processing parameters
SI 65536
SF 470.5929072 MHz
WDW EM
SSB 0
LB 0.30 Hz
GB 0
PC 1.00
0
-20
-40
-60
-80
-100
-120
-140
-160
-180
ppm

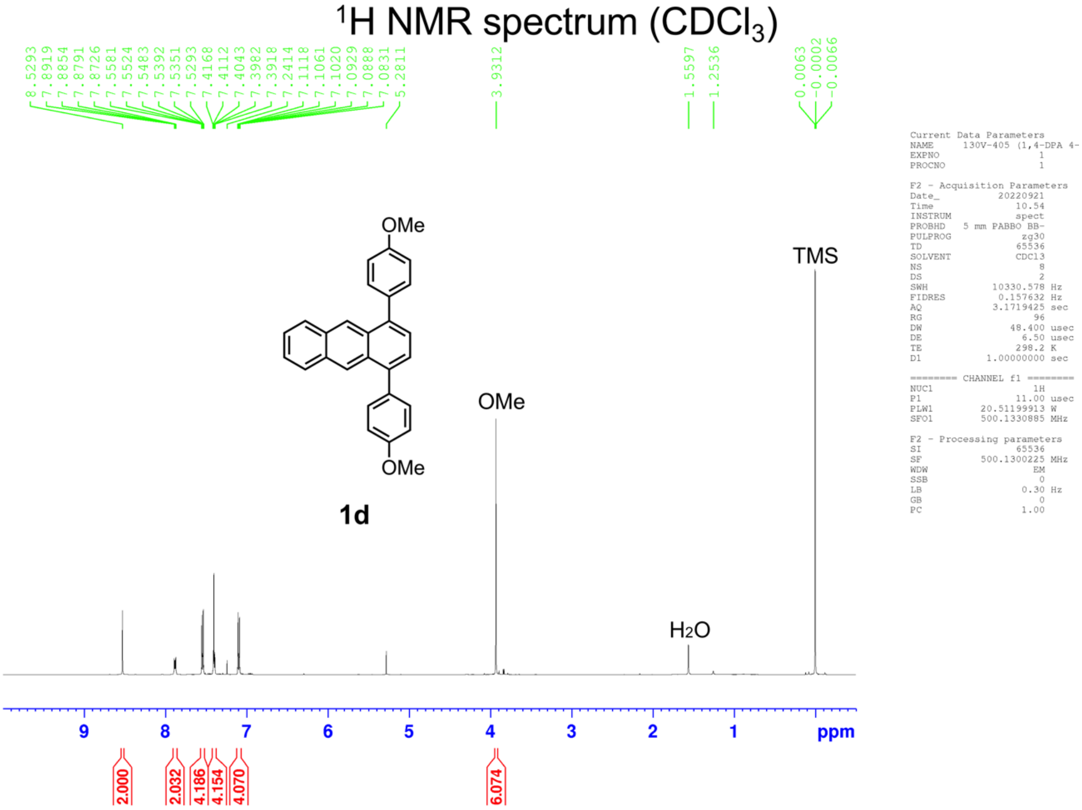
1H NMR spectrum (CDCl3)
8.5293
7.8919
7.8854
7.8791
7.8726
7.5581
7.5524
7.5483
7.5392
7.5351
7.5293
7.4168
7.4112
7.4043
7.3982
7.3918
7.2414
7.1118
7.1061
7.1020
7.0929
7.0888
7.0831
5.2811
3.9312
1.5597
1.2536
0.0063
-0.0002
-0.0066
OMe
OMe
1d
OMe
TMS
H2O
Current Data Parameters
NAME 130V-405 (1,4-DPA 4-
EXPNO 1
PROCNO 1
F2 - Acquisition Parameters
Date_ 20220921
Time 10.54
INSTRUM spect
PROBHD 5 mm PABBO BB-
PULPROG zg30
TD 65536
SOLVENT CDCl3
NS 8
DS 2
SWH 10330.578 Hz
FIDRES 0.157632 Hz
AQ 3.1719425 sec
RG 96
DW 48.400 usec
DE 6.50 usec
TE 298.2 K
D1 1.00000000 sec
======== CHANNEL f1 ========
NUC1 1H
P1 11.00 usec
PLW1 20.51199913 W
SFO1 500.1330885 MHz
F2 - Processing parameters
SI 65536
SF 500.1300225 MHz
WDW EM
SSB 0
LB 0.30 Hz
GB 0
PC 1.00
9
8
7
6
5
4
3
2
1
ppm
2.000
2.032
4.186
4.154
4.070
6.074

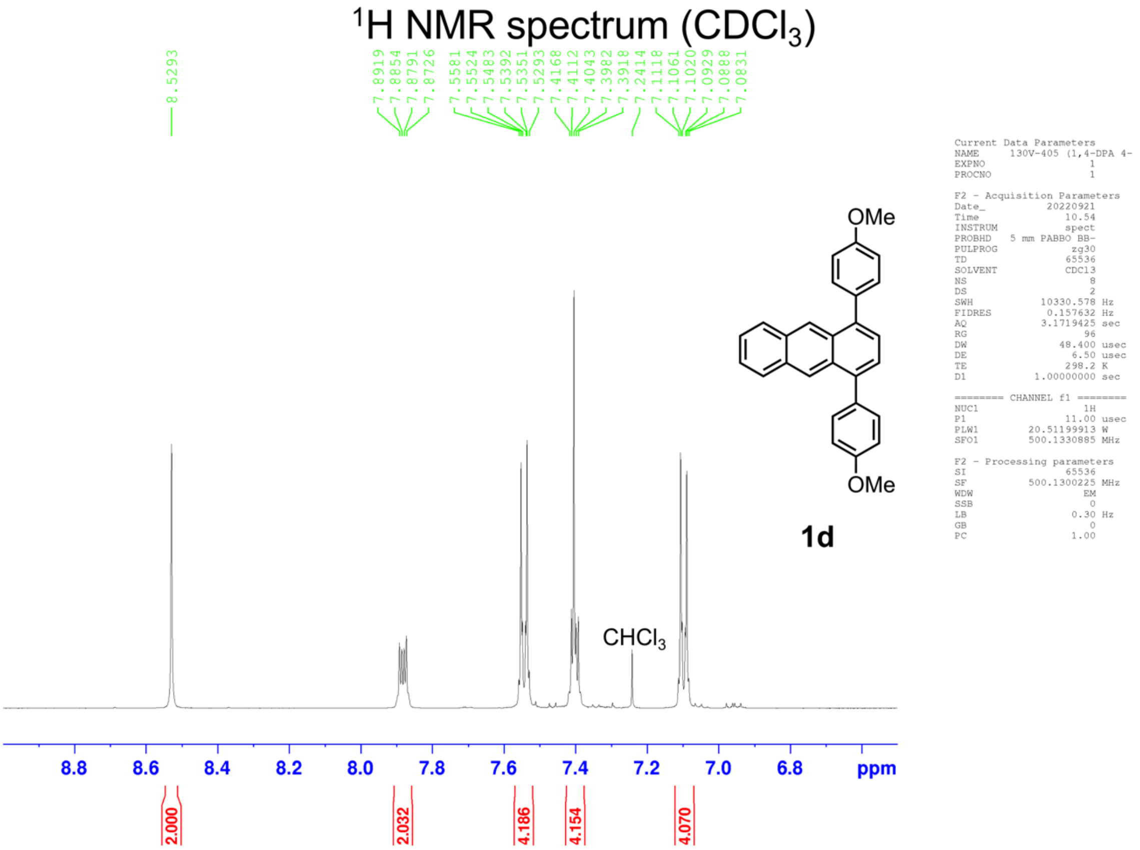

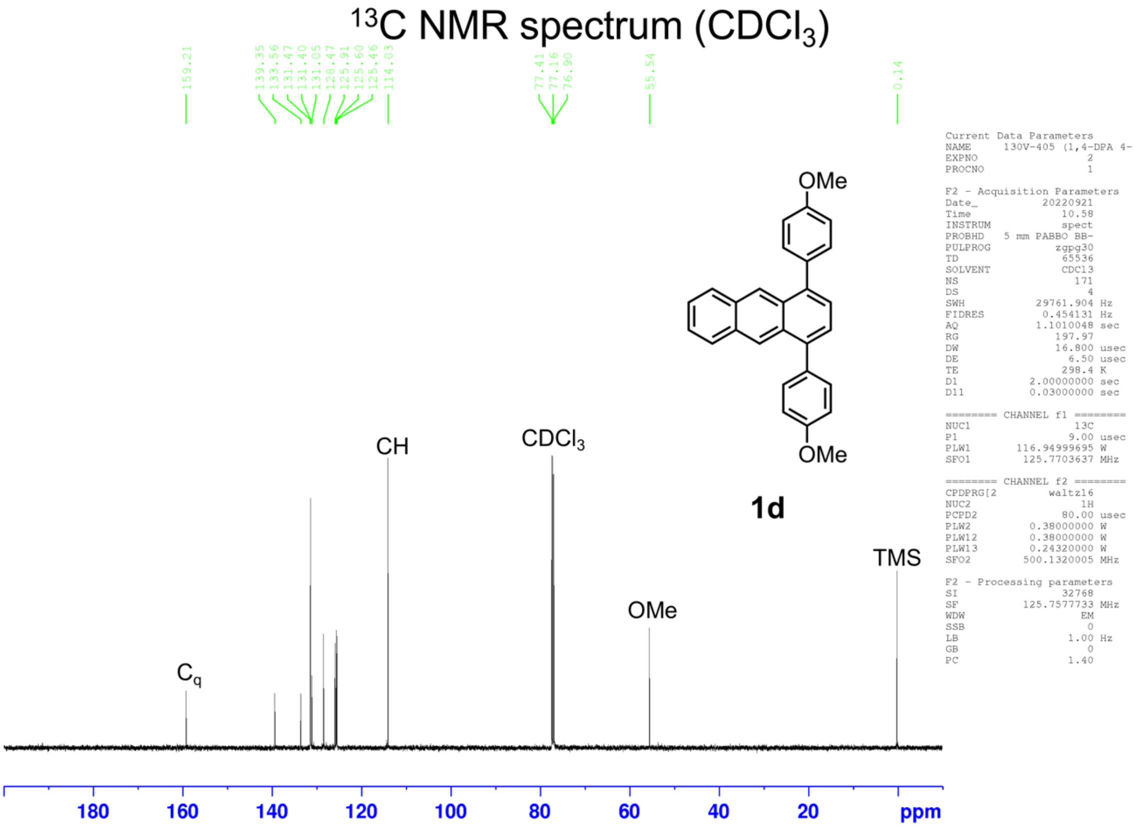

^{13}C NMR spectrum ($CDCl_3$)

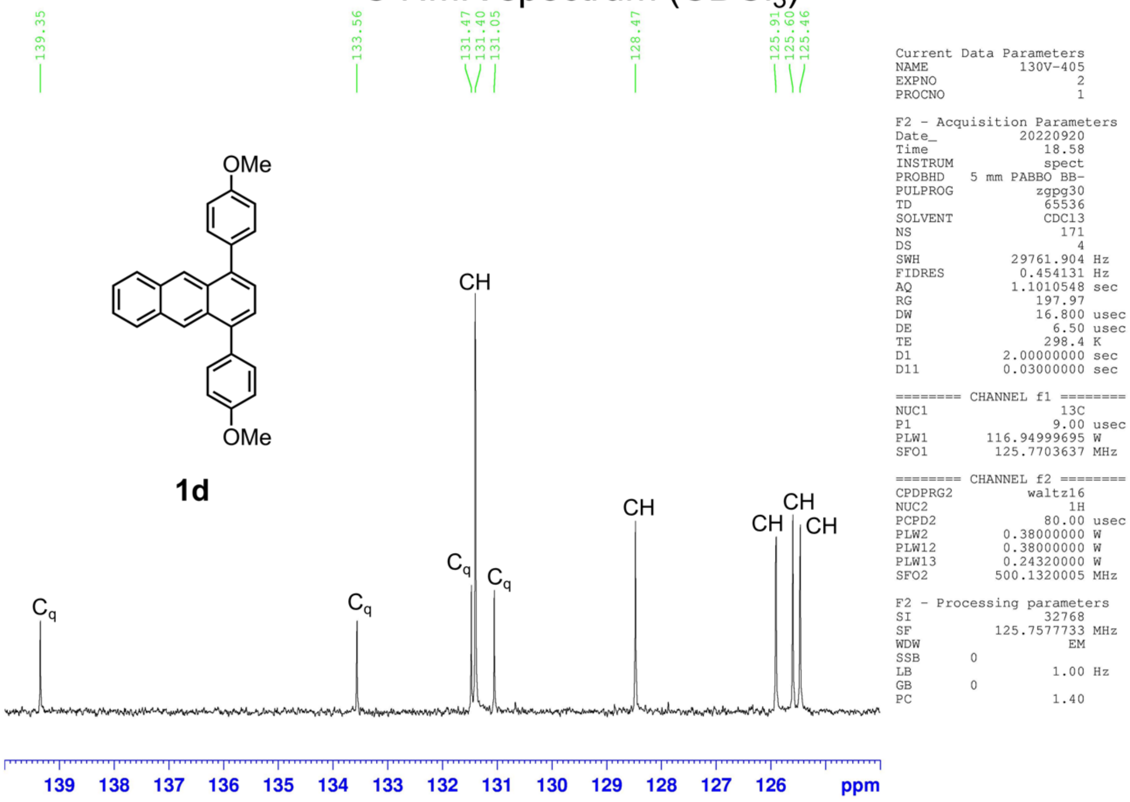

DEPT 135 NMR spectrum ($CDCl_3$)

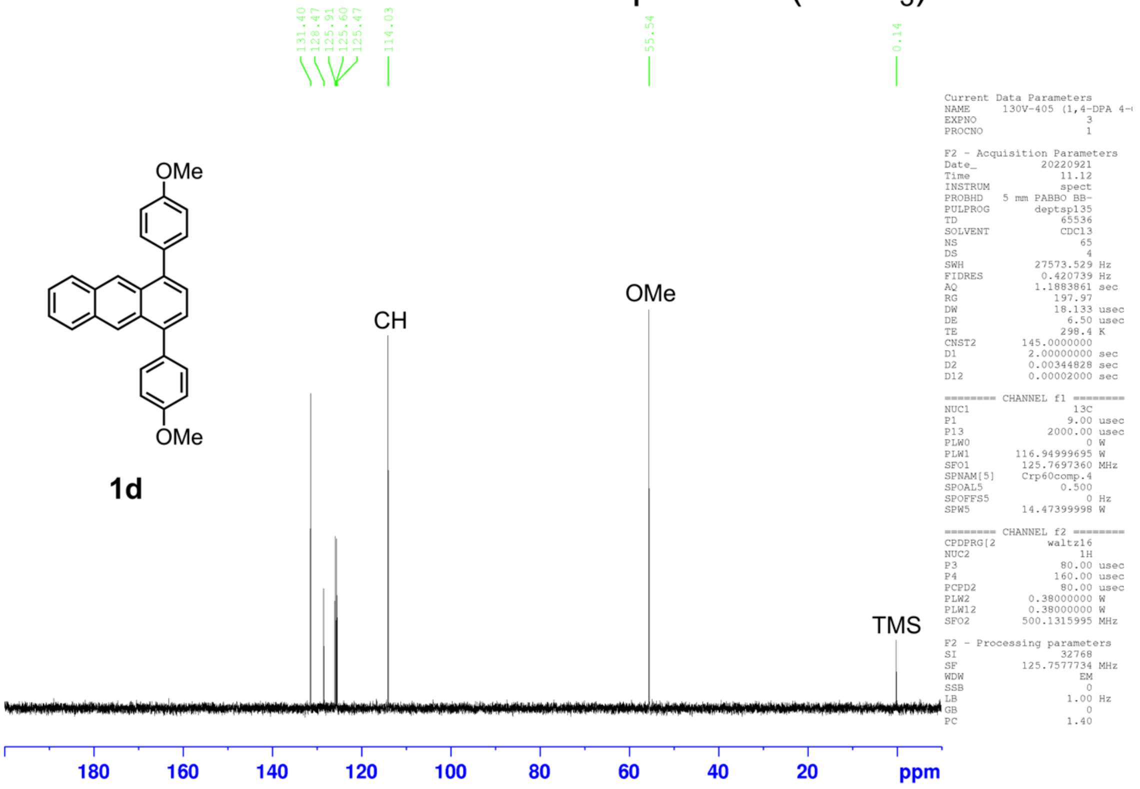

DEPT 135 NMR spectrum ($CDCl_3$)

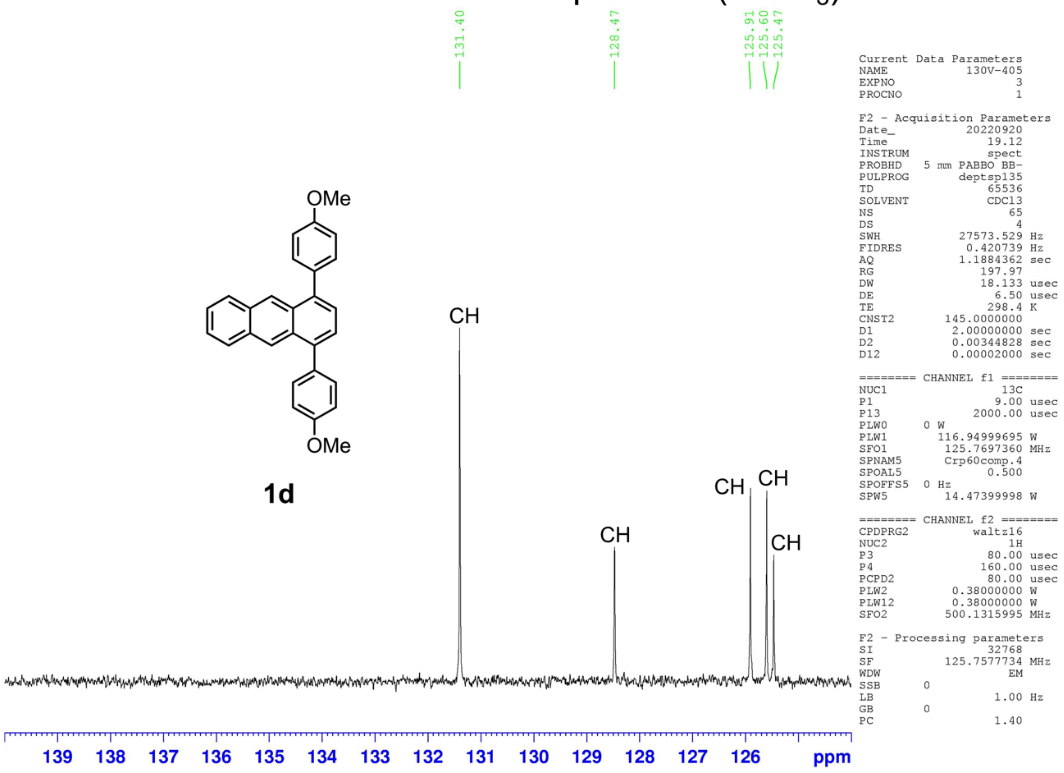

^1H NMR spectrum ($CDCl_3$)

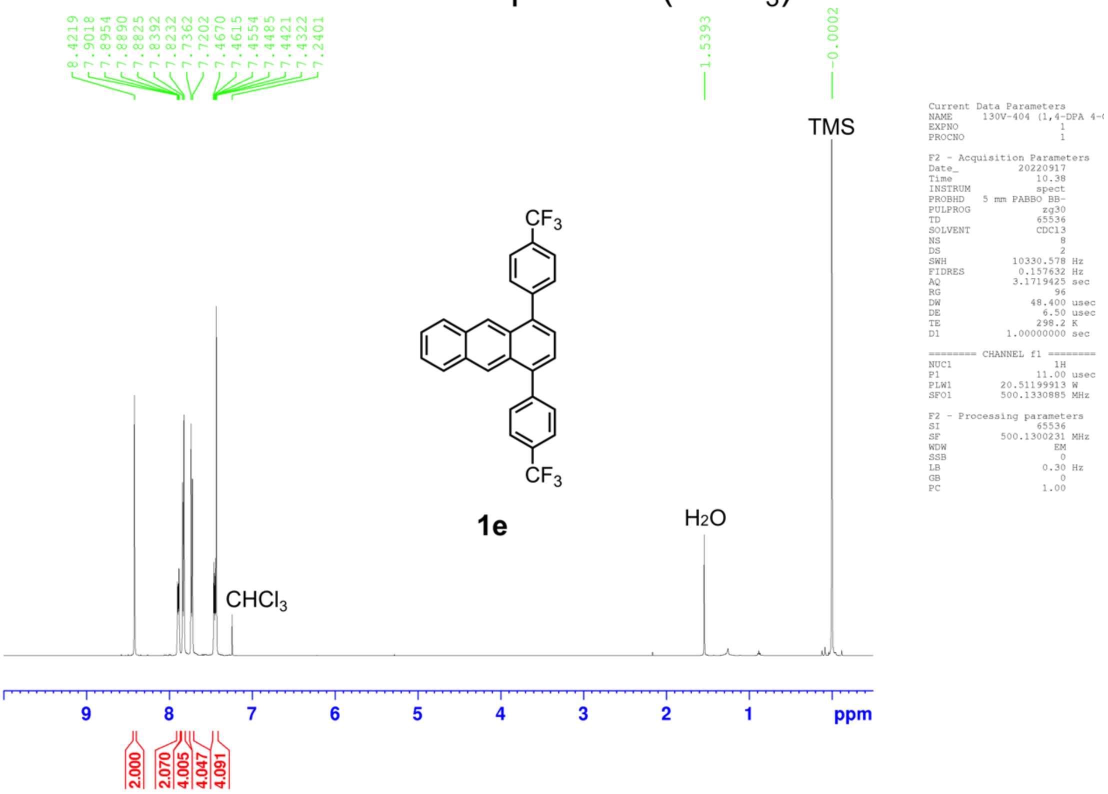

^{1}H NMR spectrum ($CDCl_3$)

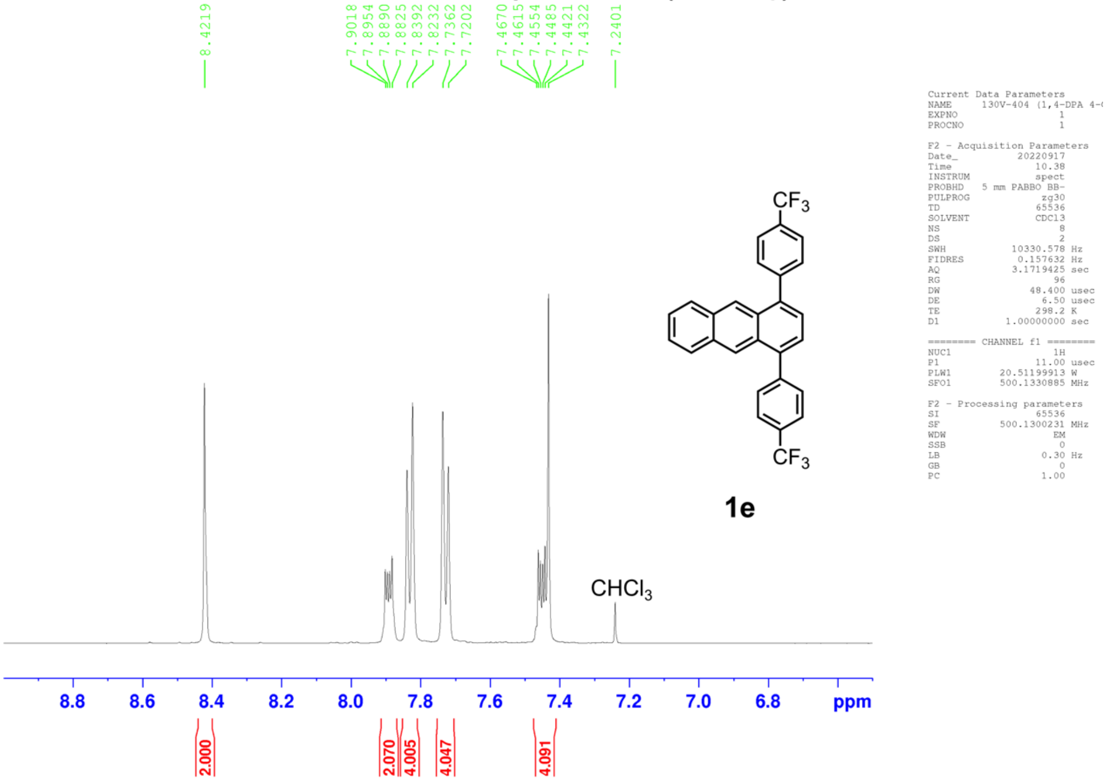

^{13}C NMR spectrum ($CDCl_3$)

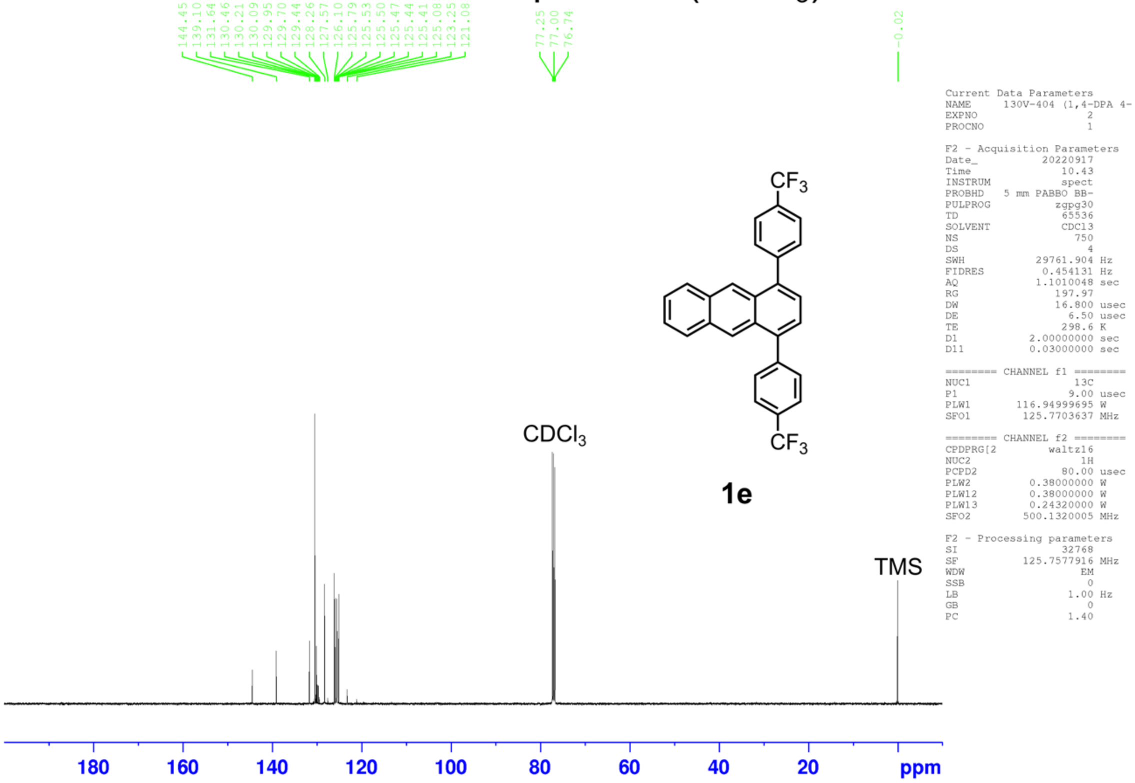

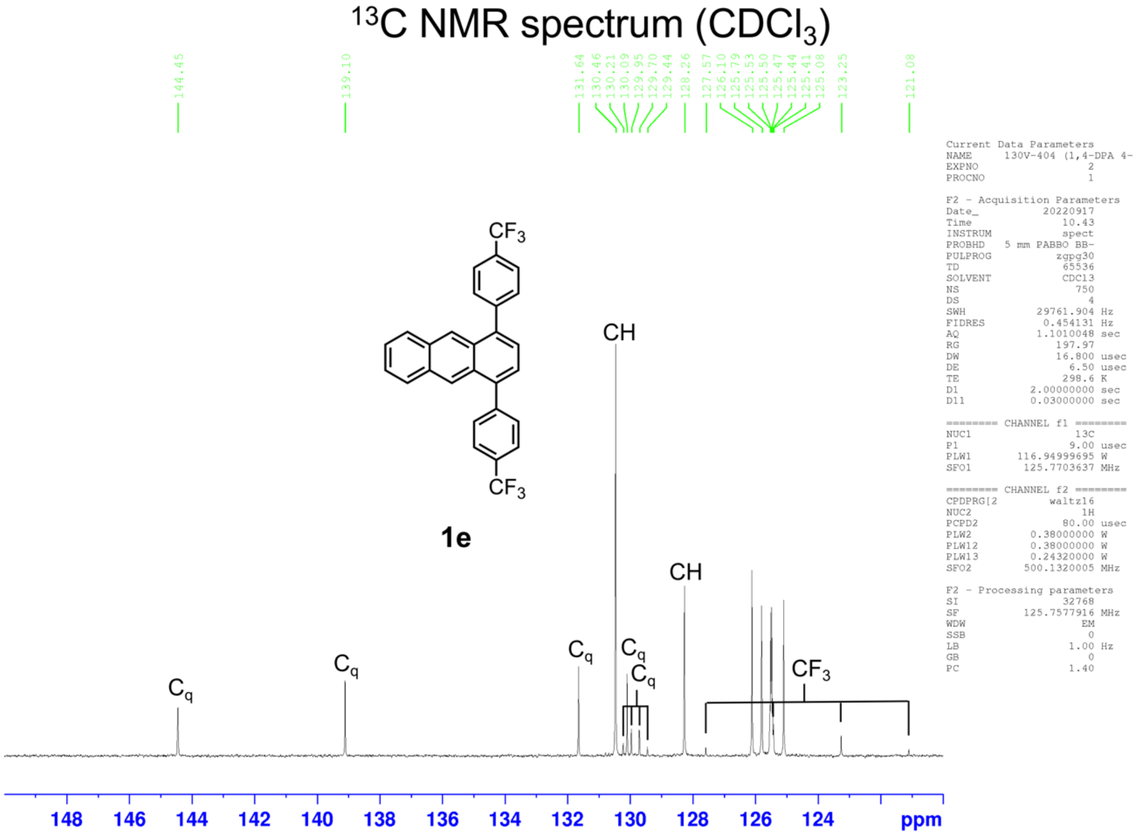

13C NMR spectrum (CDCl3)
144.45
139.10
131.64
130.46
130.21
130.09
129.95
129.70
129.44
128.26
127.57
126.10
125.79
125.53
125.50
125.47
125.44
125.41
125.08
123.25
121.08
CF3
CF3
1e
CH
CH
Cq
Cq
Cq
Cq
Cq
CF3
148 146 144 142 140 138 136 134 132 130 128 126 124 ppm
Current Data Parameters
NAME 130V-404 (1,4-DPA 4-
EXPNO 2
PROCNO 1
F2 - Acquisition Parameters
Date_ 20220917
Time 10.43
INSTRUM spect
PROBHD 5 mm PABBO BB-
PULPROG zgpg30
TD 65536
SOLVENT CDCl3
NS 750
DS 4
SWH 29761.904 Hz
FIDRES 0.454131 Hz
AQ 1.1010048 sec
RG 197.97
DW 16.800 usec
DE 6.50 usec
TE 298.6 K
D1 2.00000000 sec
D11 0.03000000 sec
======== CHANNEL f1 ========
NUC1 13C
P1 9.00 usec
PLW1 116.94999695 W
SFO1 125.7703637 MHz
======== CHANNEL f2 ========
CPDPRG[2 waltz16
NUC2 1H
PCPD2 80.00 usec
PLW2 0.38000000 W
PLW12 0.38000000 W
PLW13 0.24320000 W
SFO2 500.1320005 MHz
F2 - Processing parameters
SI 32768
SF 125.7577916 MHz
WDW EM
SSB 0
LB 1.00 Hz
GB 0
PC 1.40

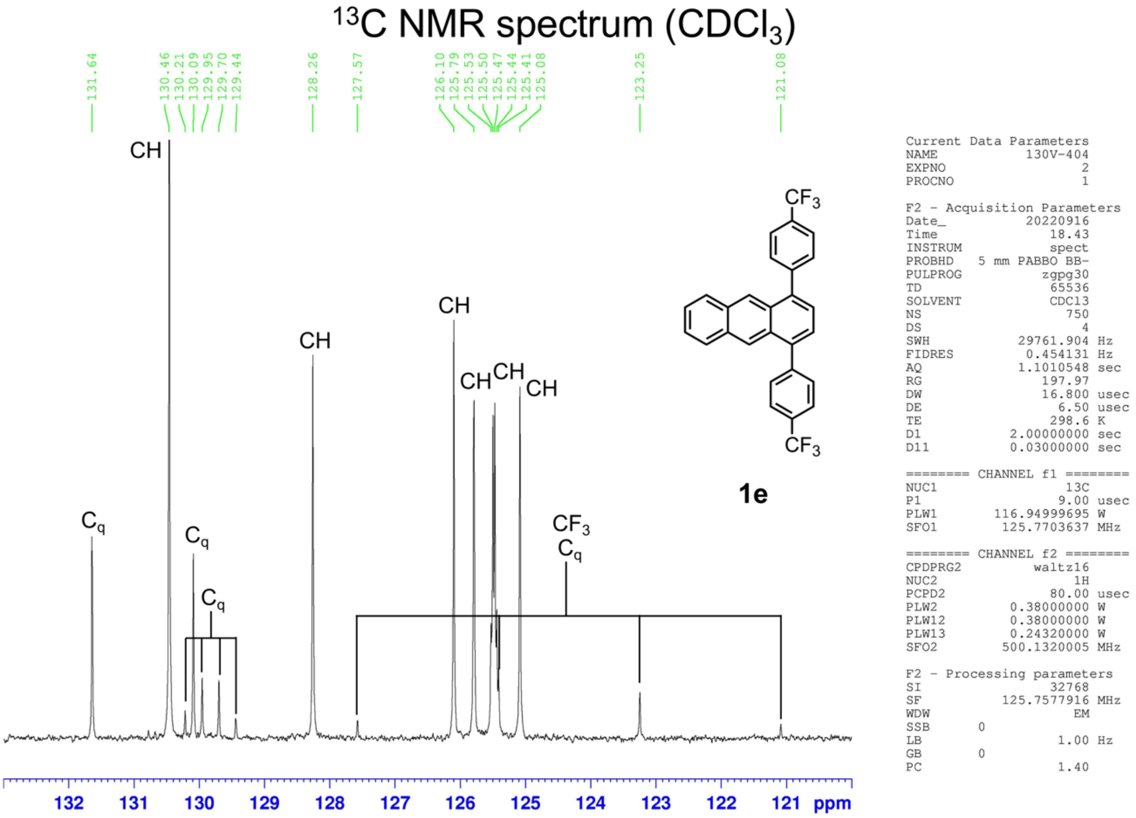

13C NMR spectrum (CDCl3)
131.64
130.46
130.21
130.09
129.95
129.70
129.44
128.26
127.57
126.10
125.79
125.53
125.50
125.47
125.44
125.41
125.08
123.25
121.08
CH
CH
CH
CH
CH
CH
Cq
Cq
Cq
CF3
Cq
CF3
CF3
1e
132 131 130 129 128 127 126 125 124 123 122 121 ppm
Current Data Parameters
NAME 130V-404
EXPNO 2
PROCNO 1
F2 - Acquisition Parameters
Date_ 20220916
Time 18.43
INSTRUM spect
PROBHD 5 mm PABBO BB-
PULPROG zgpg30
TD 65536
SOLVENT CDCl3
NS 750
DS 4
SWH 29761.904 Hz
FIDRES 0.454131 Hz
AQ 1.1010548 sec
RG 197.97
DW 16.800 usec
DE 6.50 usec
TE 298.6 K
D1 2.00000000 sec
D11 0.03000000 sec
======== CHANNEL f1 ========
NUC1 13C
P1 9.00 usec
PLW1 116.94999695 W
SFO1 125.7703637 MHz
======== CHANNEL f2 ========
CPDPRG2 waltz16
NUC2 1H
PCPD2 80.00 usec
PLW2 0.38000000 W
PLW12 0.38000000 W
PLW13 0.24320000 W
SFO2 500.1320005 MHz
F2 - Processing parameters
SI 32768
SF 125.7577916 MHz
WDW EM
SSB 0
LB 1.00 Hz
GB 0
PC 1.40

^{13}C NMR spectrum ($CDCl_3$)

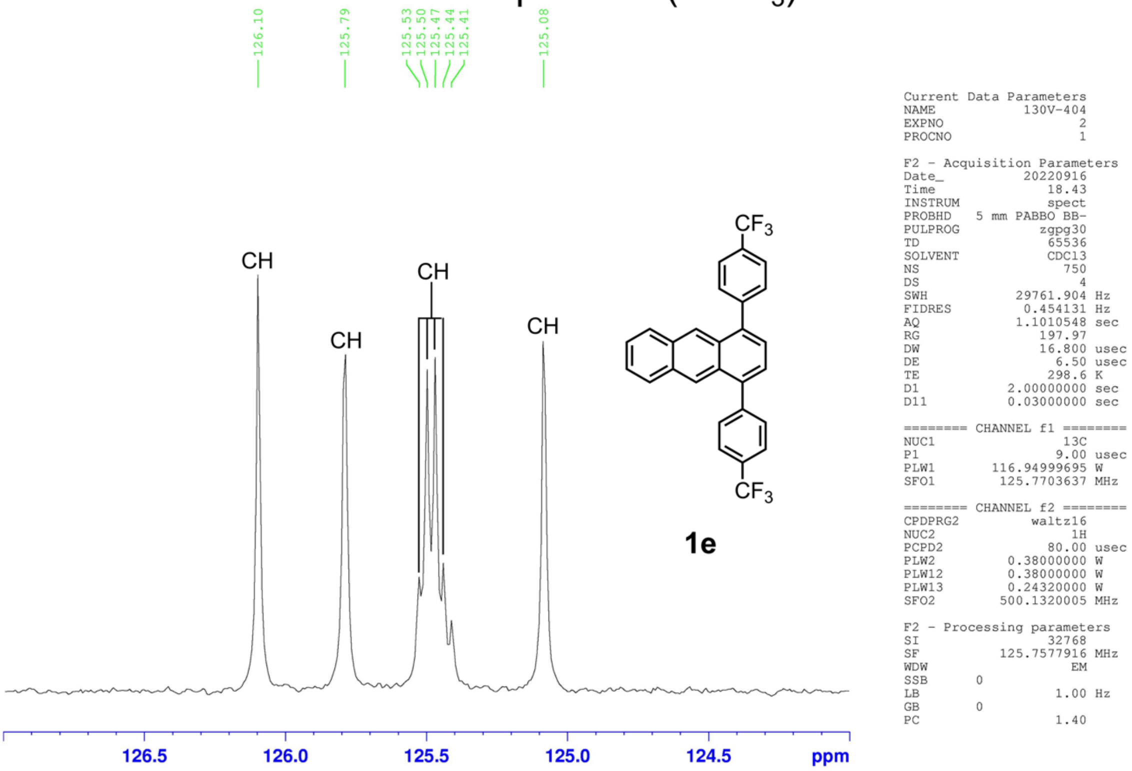

DEPT 135 NMR spectrum ($CDCl_3$)

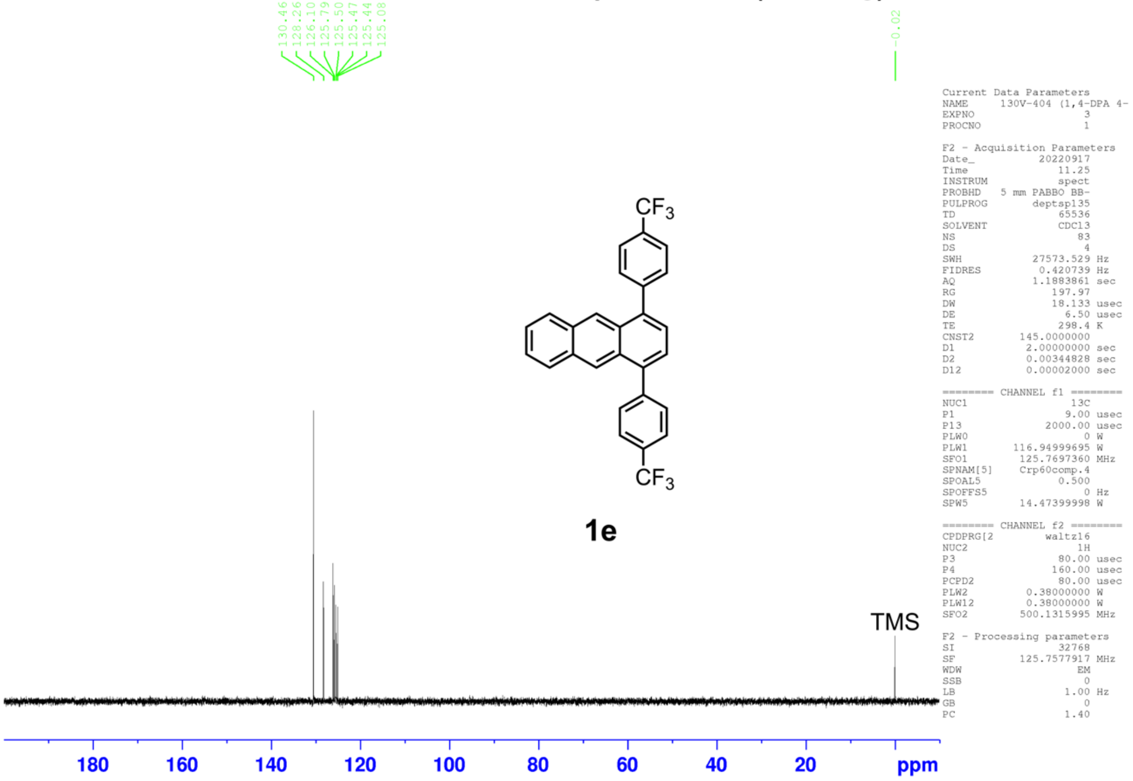

DEPT 135 NMR spectrum ($CDCl_3$)

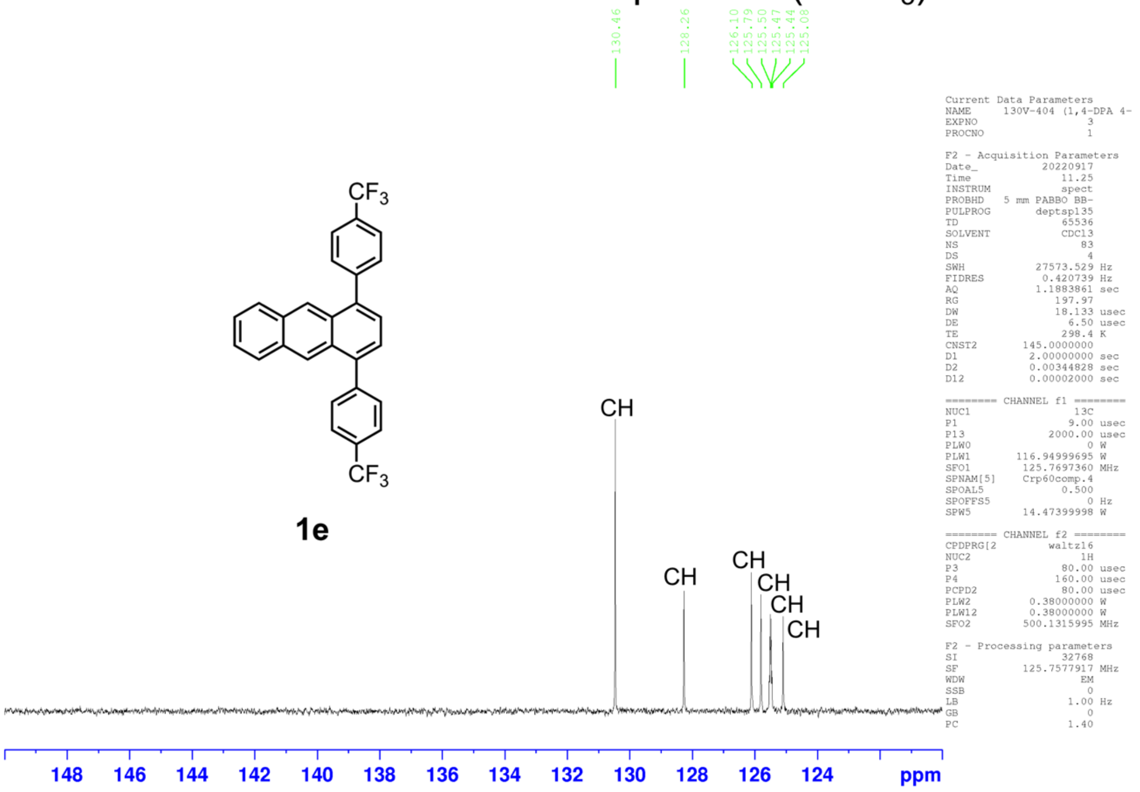

^{19}F NMR spectrum ($CDCl_3$)

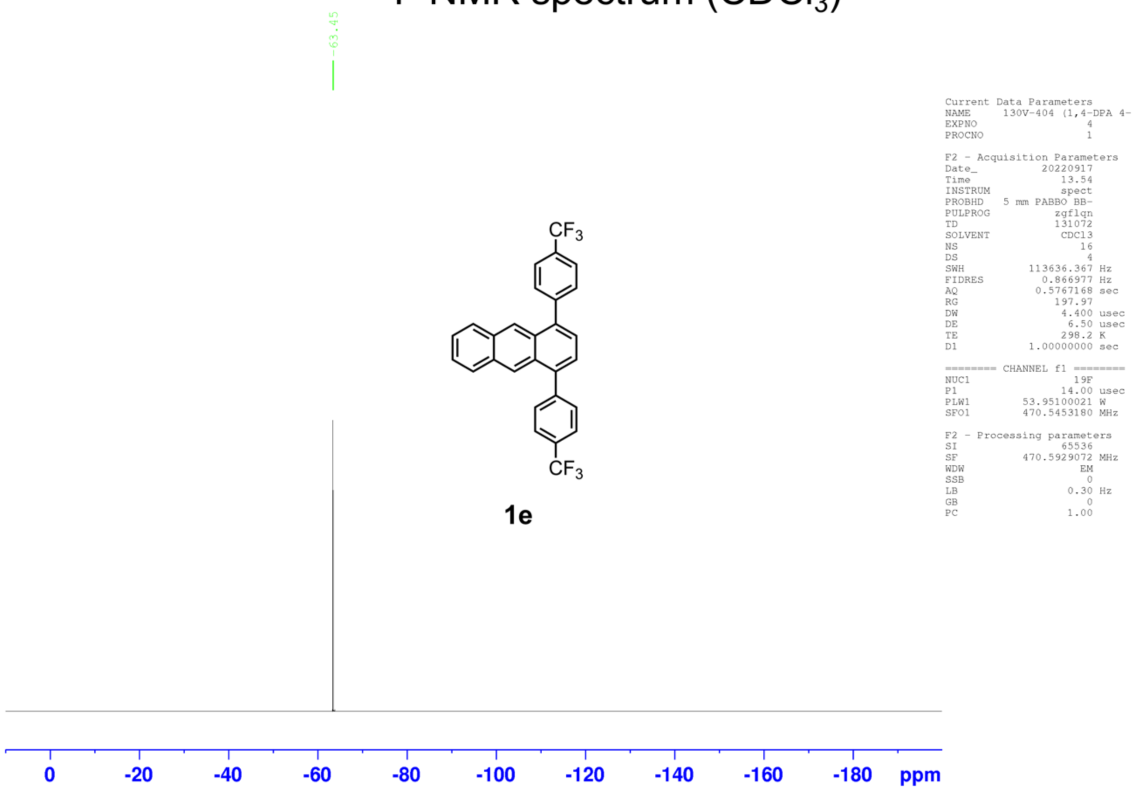

^1H NMR spectrum ($CDCl_3$)

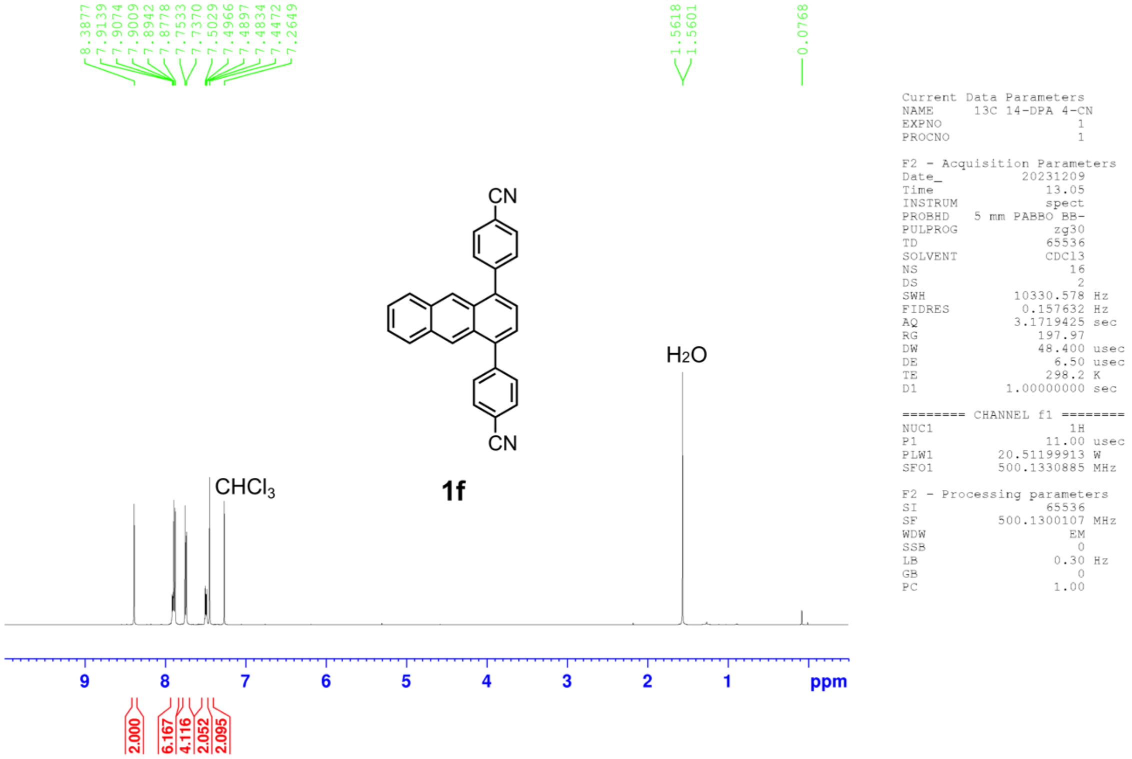

^1H NMR spectrum ($CDCl_3$)

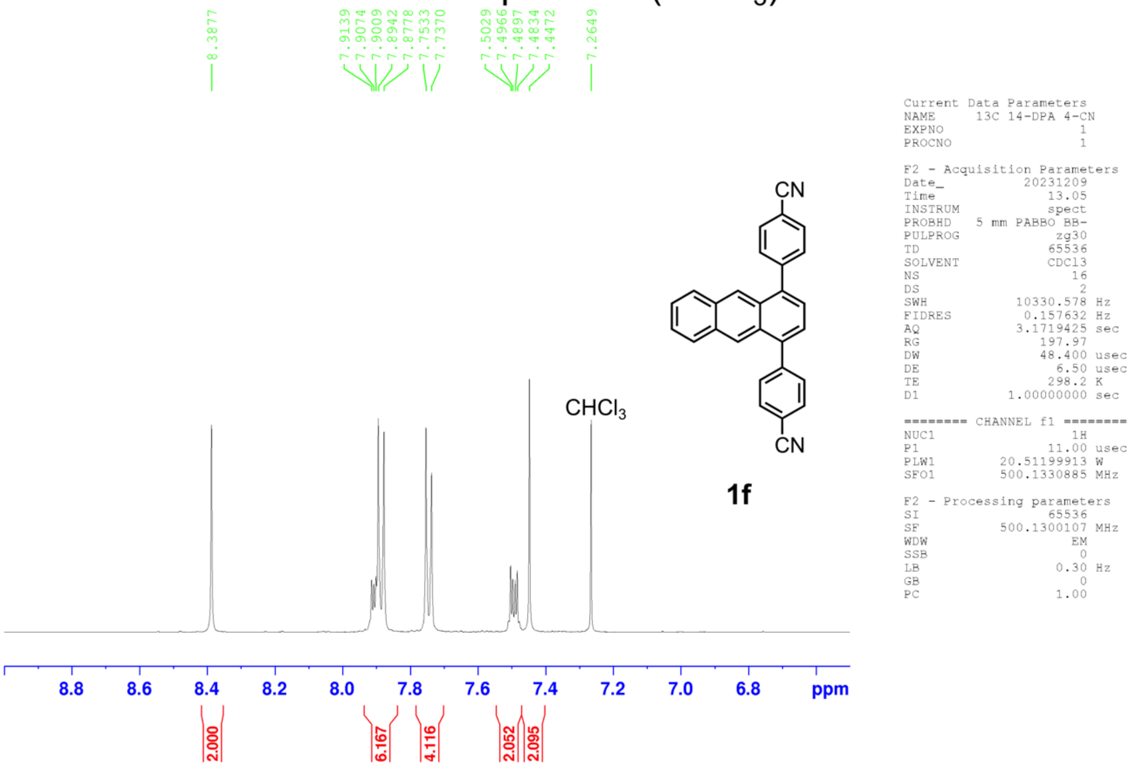

^{13}C NMR spectrum ($CDCl_3$)

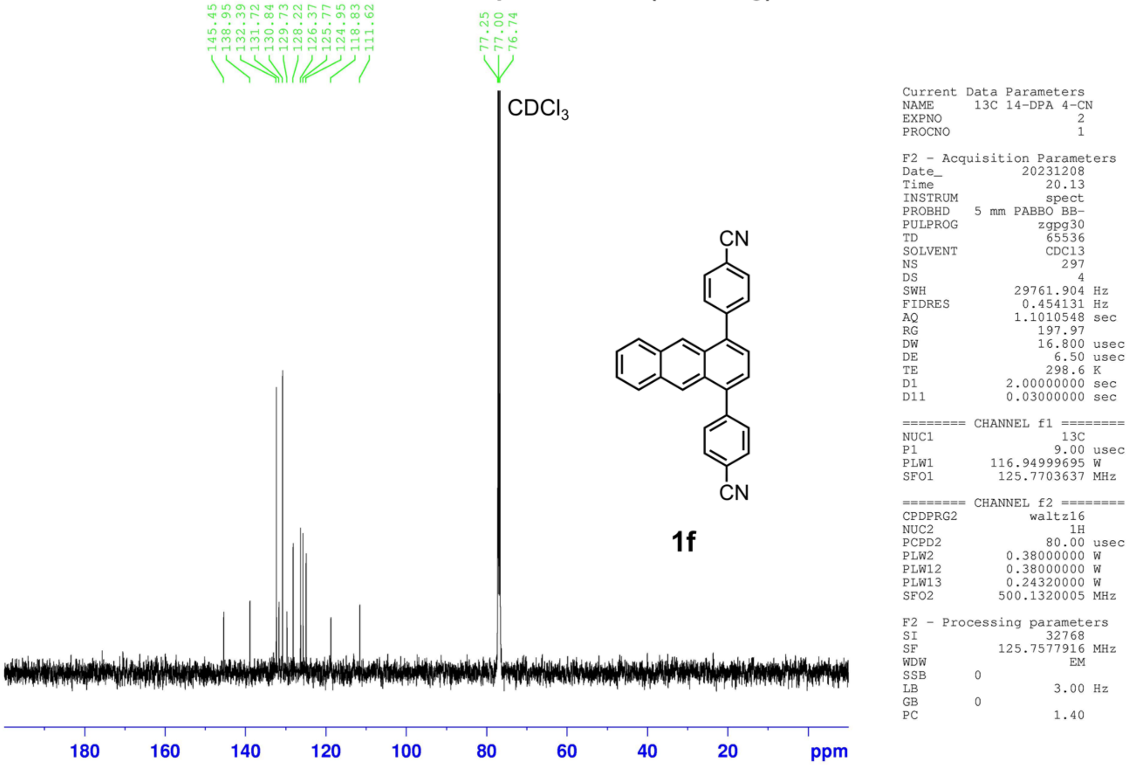

^{13}C NMR spectrum ($CDCl_3$)

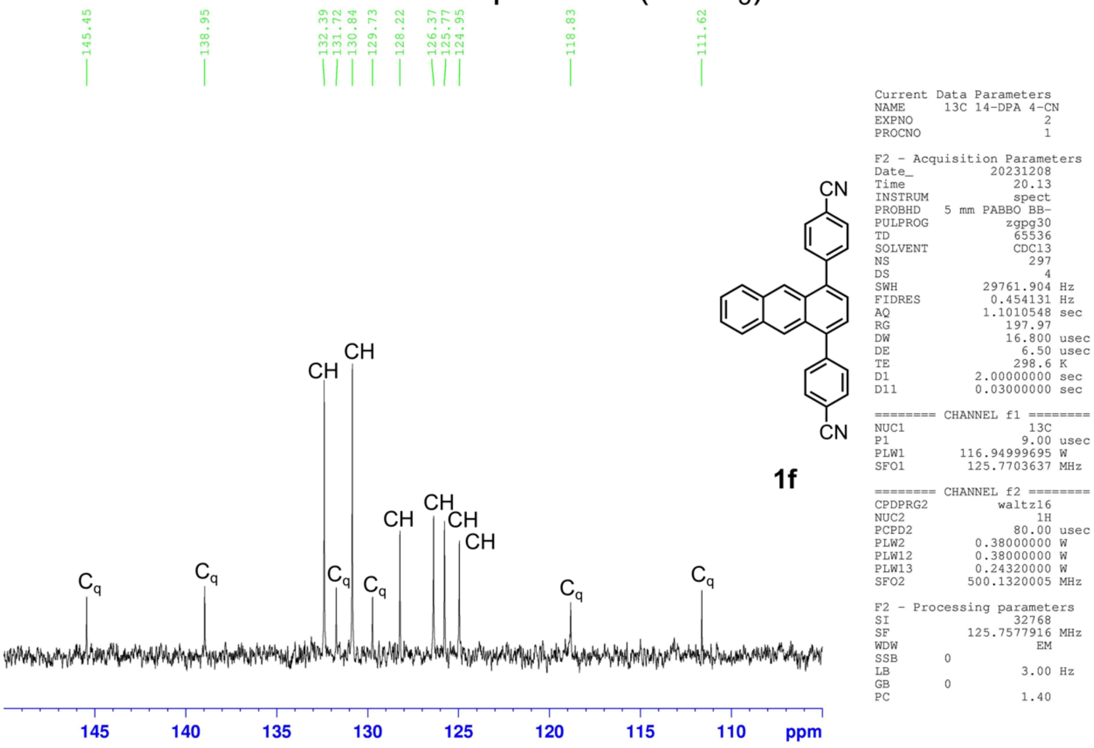

DEPT 135 NMR spectrum ($CDCl_3$)

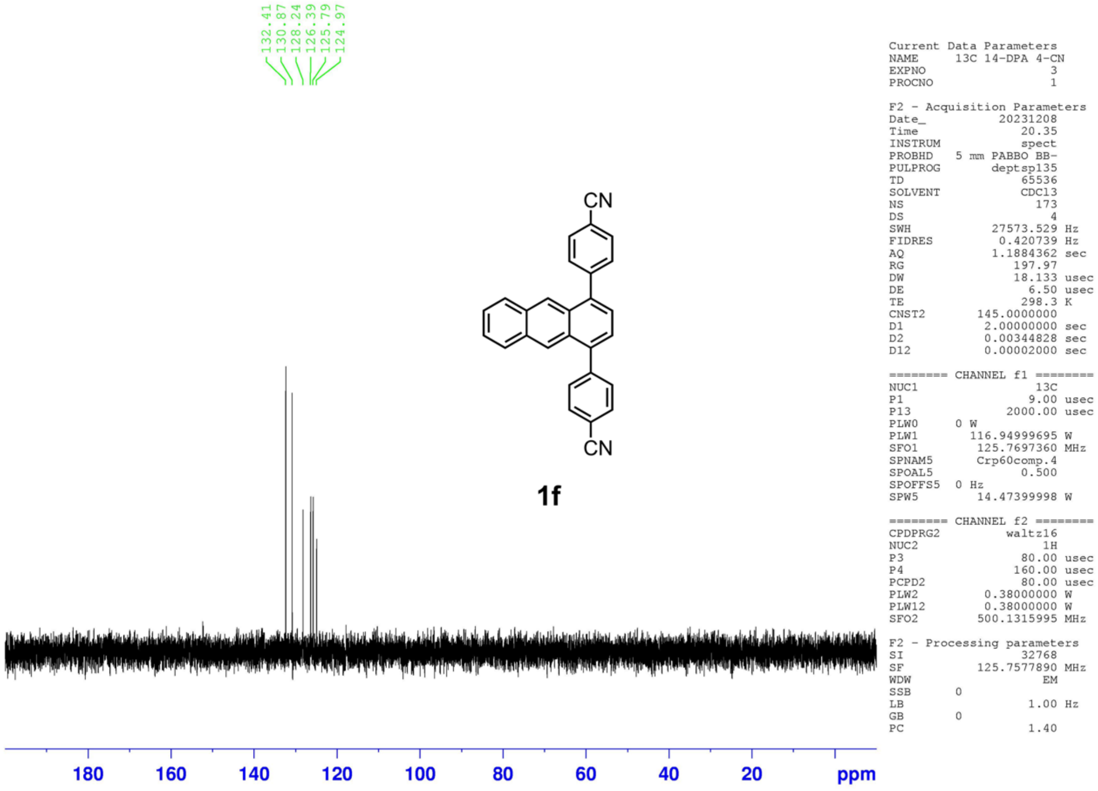

DEPT 135 NMR spectrum ($CDCl_3$)

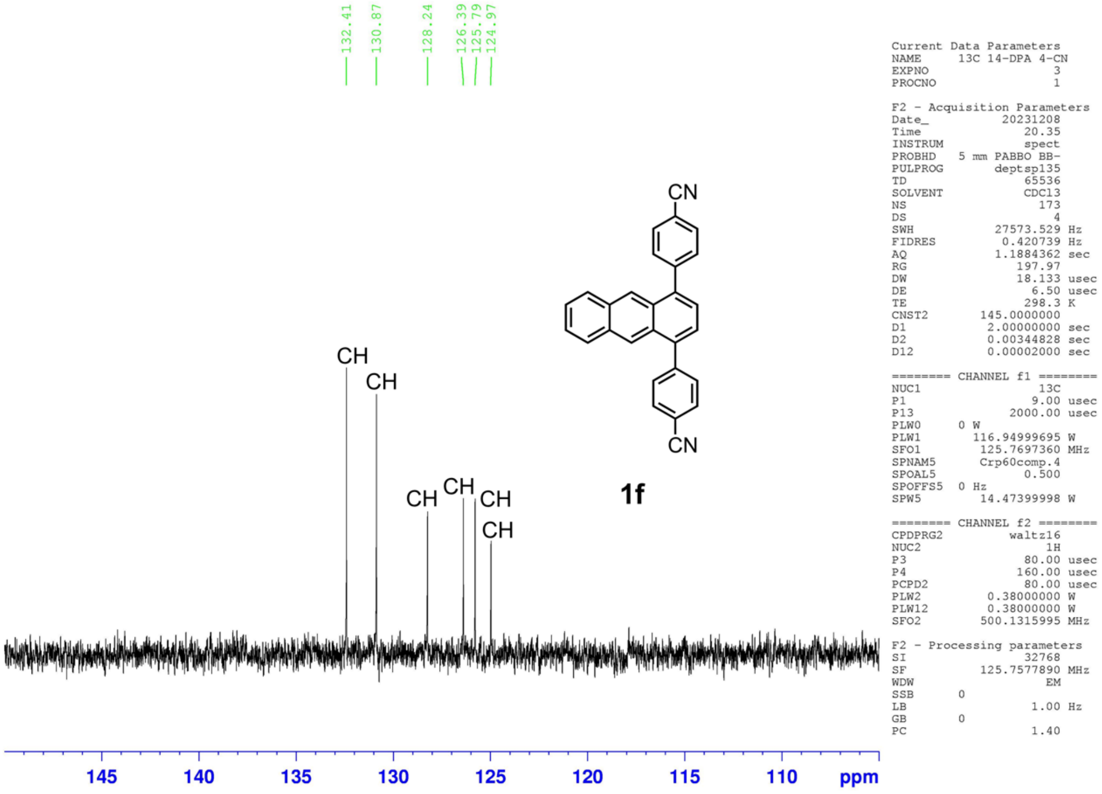

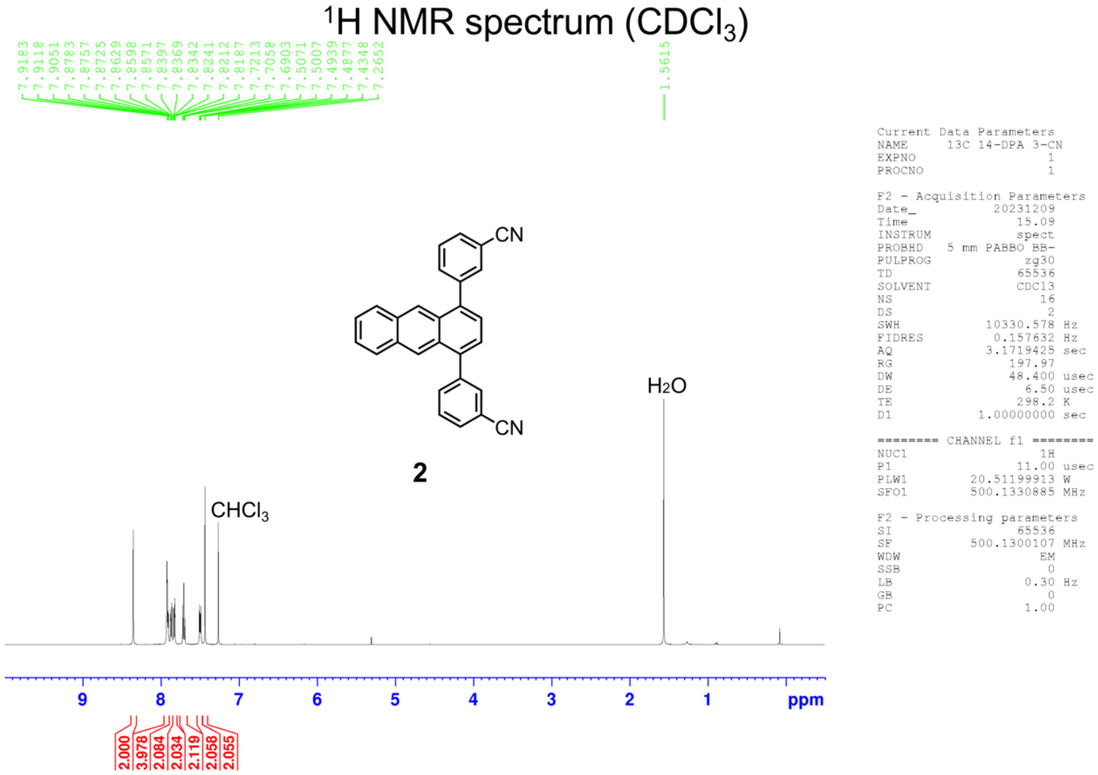
1H NMR spectrum (CDCl3)
7.9183 7.9118 7.9051 7.8783 7.8757 7.8725 7.8629 7.8598 7.8571 7.8397 7.8369 7.8342 7.8241 7.8212 7.8187 7.7213 7.7058 7.6903 7.5071 7.5007 7.4939 7.4877 7.4348 7.2652
1.5615
CN
CN
2
H2O
CHCl3
9 8 7 6 5 4 3 2 1 ppm
2.000 3.978 2.084 2.034 2.119 2.058 2.055
Current Data Parameters
NAME 13C 14-DPA 3-CN
EXPNO 1
PROCNO 1
F2 - Acquisition Parameters
Date_ 20231209
Time 15.09
INSTRUM spect
PROBHD 5 mm PABBO BB-
PULPROG zg30
TD 65536
SOLVENT CDCl3
NS 16
DS 2
SWH 10330.578 Hz
FIDRES 0.157632 Hz
AQ 3.1719425 sec
RG 197.97
DW 48.400 usec
DE 6.50 usec
TE 298.2 K
D1 1.00000000 sec
======== CHANNEL f1 ========
NUC1 1H
P1 11.00 usec
PLW1 20.51199913 W
SFO1 500.1330885 MHz
F2 - Processing parameters
SI 65536
SF 500.1300107 MHz
WDW EM
SSB 0
LB 0.30 Hz
GB 0
PC 1.00

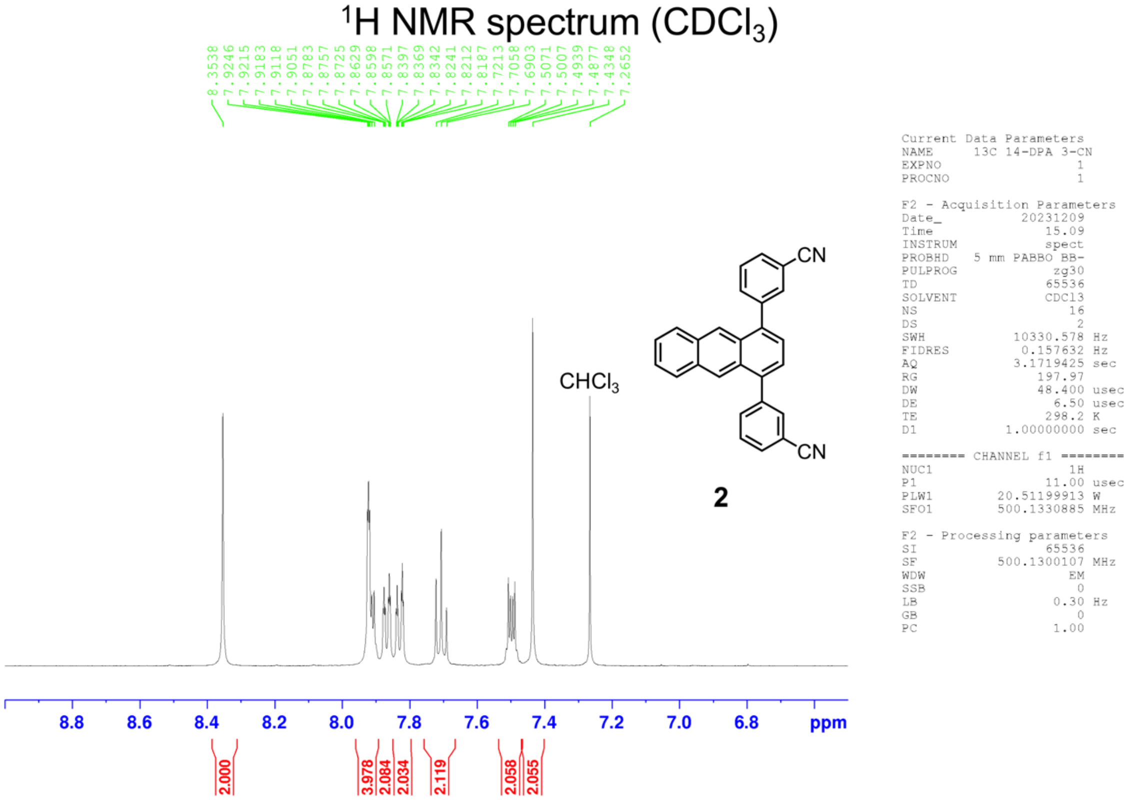
1H NMR spectrum (CDCl3)
8.3538 7.9246 7.9215 7.9183 7.9118 7.9051 7.8783 7.8757 7.8725 7.8629 7.8598 7.8571 7.8397 7.8369 7.8342 7.8241 7.8212 7.8187 7.7213 7.7058 7.6903 7.5071 7.5007 7.4939 7.4877 7.4348 7.2652
CN
CN
2
CHCl3
8.8 8.6 8.4 8.2 8.0 7.8 7.6 7.4 7.2 7.0 6.8 ppm
2.000 3.978 2.084 2.034 2.119 2.058 2.055
Current Data Parameters
NAME 13C 14-DPA 3-CN
EXPNO 1
PROCNO 1
F2 - Acquisition Parameters
Date_ 20231209
Time 15.09
INSTRUM spect
PROBHD 5 mm PABBO BB-
PULPROG zg30
TD 65536
SOLVENT CDCl3
NS 16
DS 2
SWH 10330.578 Hz
FIDRES 0.157632 Hz
AQ 3.1719425 sec
RG 197.97
DW 48.400 usec
DE 6.50 usec
TE 298.2 K
D1 1.00000000 sec
======== CHANNEL f1 ========
NUC1 1H
P1 11.00 usec
PLW1 20.51199913 W
SFO1 500.1330885 MHz
F2 - Processing parameters
SI 65536
SF 500.1300107 MHz
WDW EM
SSB 0
LB 0.30 Hz
GB 0
PC 1.00

^{13}C NMR spectrum ($CDCl_3$)

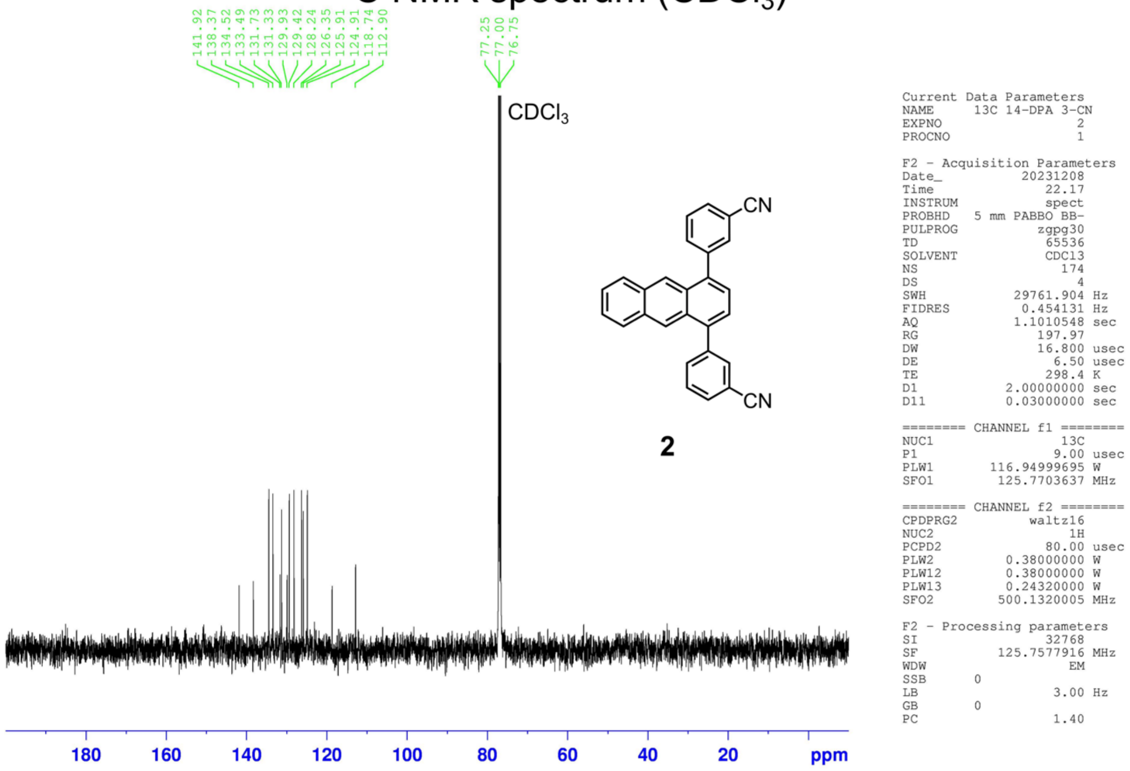

^{13}C NMR spectrum ($CDCl_3$)

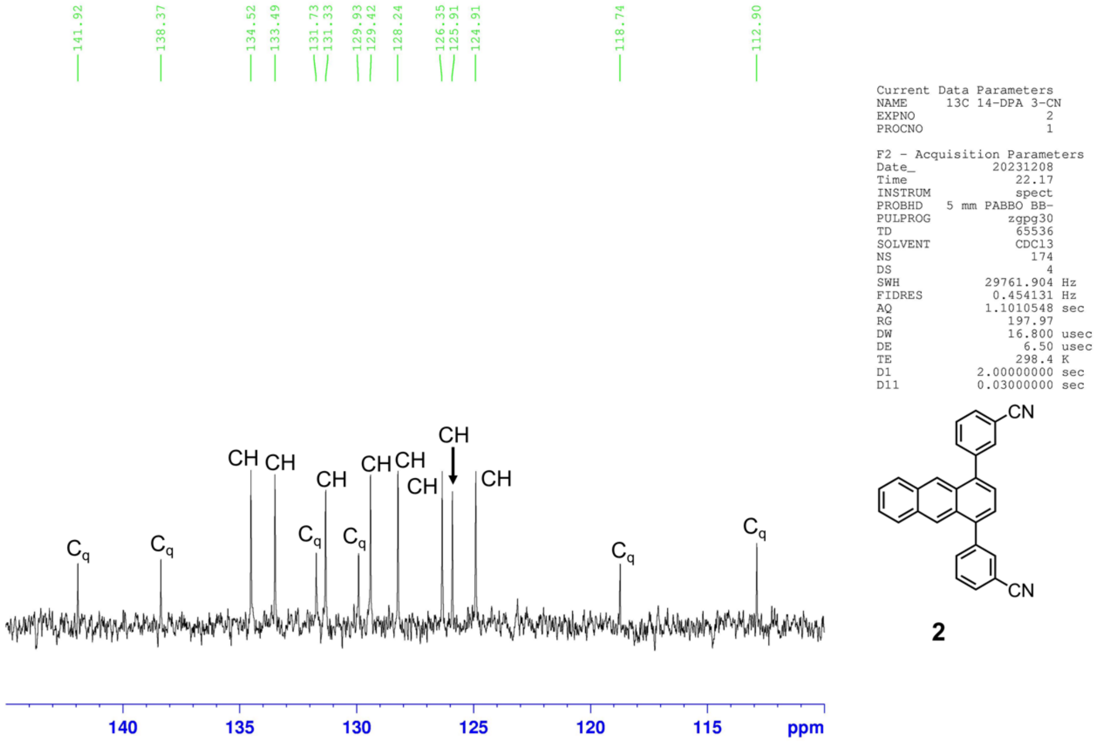

DEPT 135 NMR spectrum ($CDCl_3$)

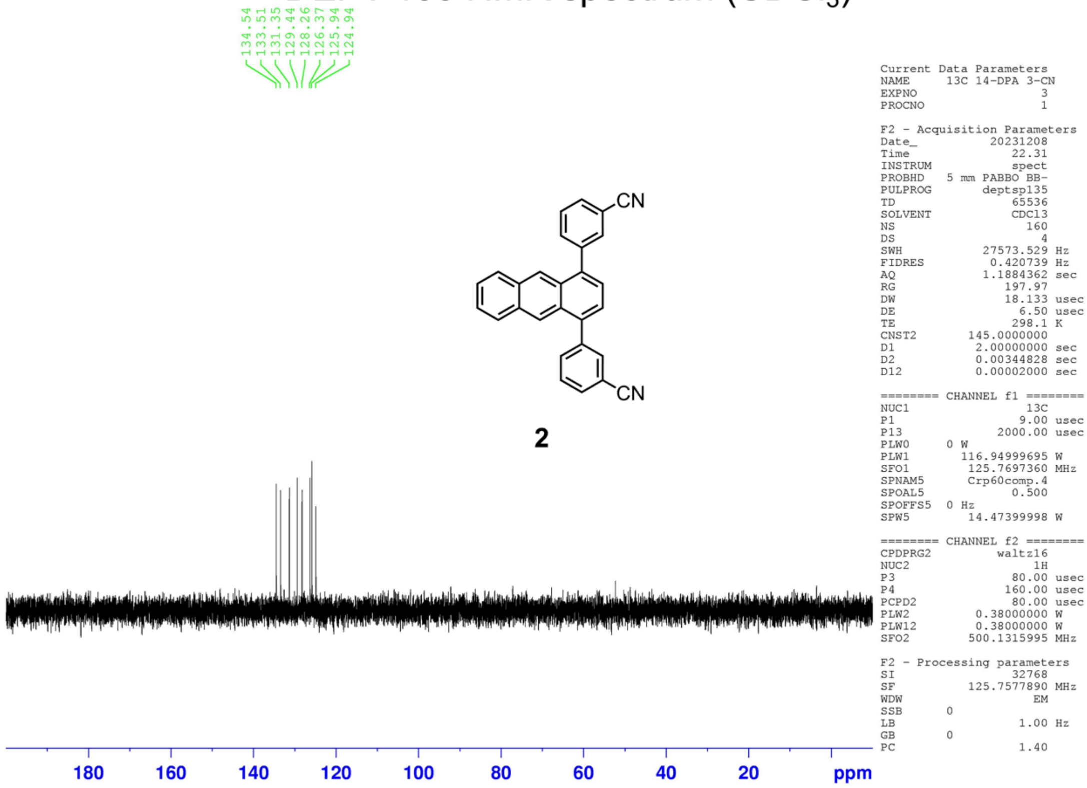

DEPT 135 NMR spectrum ($CDCl_3$)

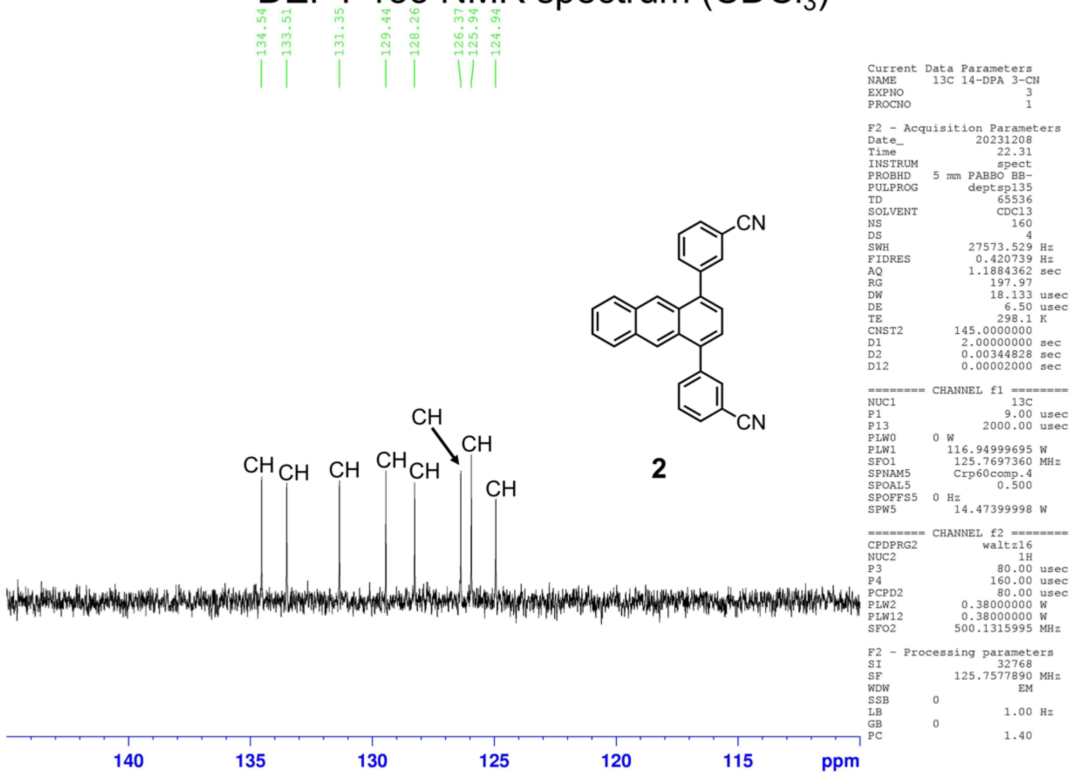

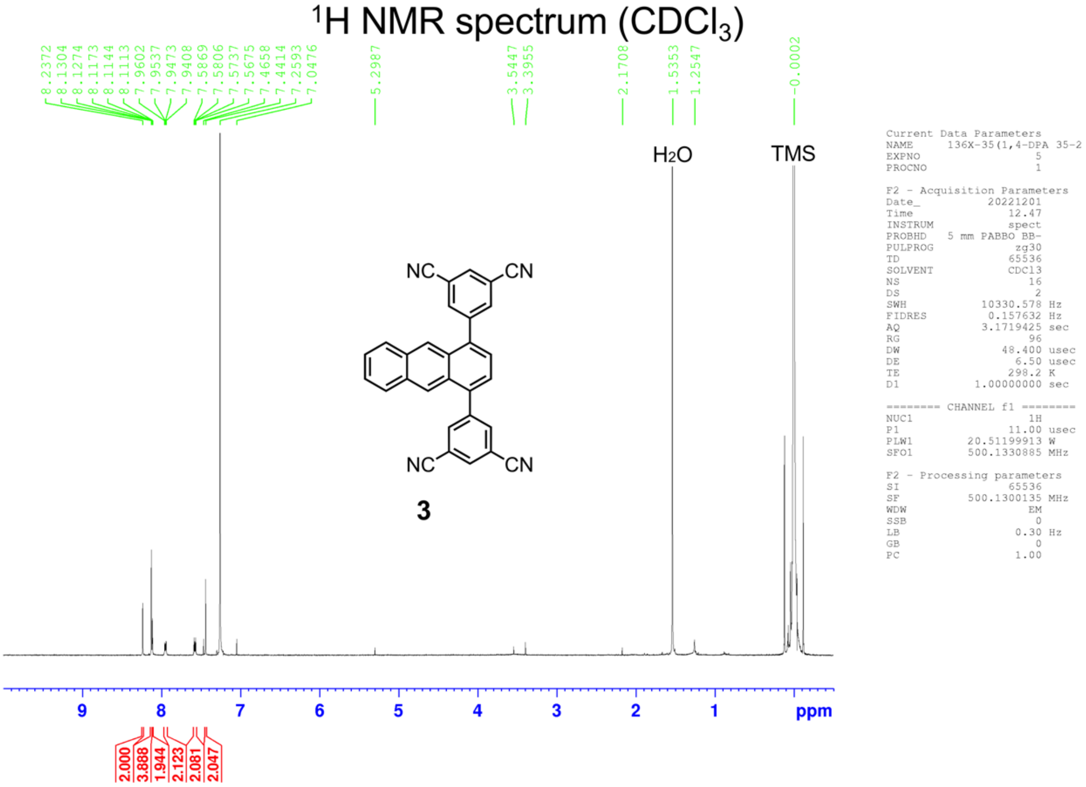

1H NMR spectrum (CDCl3)
8.2372 8.1304 8.1274 8.1173 8.1144 8.1113 7.9602 7.9537 7.9473 7.9408 7.5869 7.5806 7.5737 7.5675 7.4658 7.4414 7.2593 7.0476
5.2987
3.5447 3.3955
2.1708
1.5353 1.2547
-0.0002
H2O
TMS
NC
CN
NC
CN
3
9 8 7 6 5 4 3 2 1 ppm
2.000 3.888 1.944 2.123 2.081 2.047
Current Data Parameters
NAME 136X-35(1,4-DPA 35-2
EXPNO 5
PROCNO 1
F2 - Acquisition Parameters
Date_ 20221201
Time 12.47
INSTRUM spect
PROBHD 5 mm PABBO BB-
PULPROG zg30
TD 65536
SOLVENT CDCl3
NS 16
DS 2
SWH 10330.578 Hz
FIDRES 0.157632 Hz
AQ 3.1719425 sec
RG 96
DW 48.400 usec
DE 6.50 usec
TE 298.2 K
D1 1.00000000 sec
======== CHANNEL f1 ========
NUC1 1H
P1 11.00 usec
PLW1 20.51199913 W
SFO1 500.1330885 MHz
F2 - Processing parameters
SI 65536
SF 500.1300135 MHz
WDW EM
SSB 0
LB 0.30 Hz
GB 0
PC 1.00

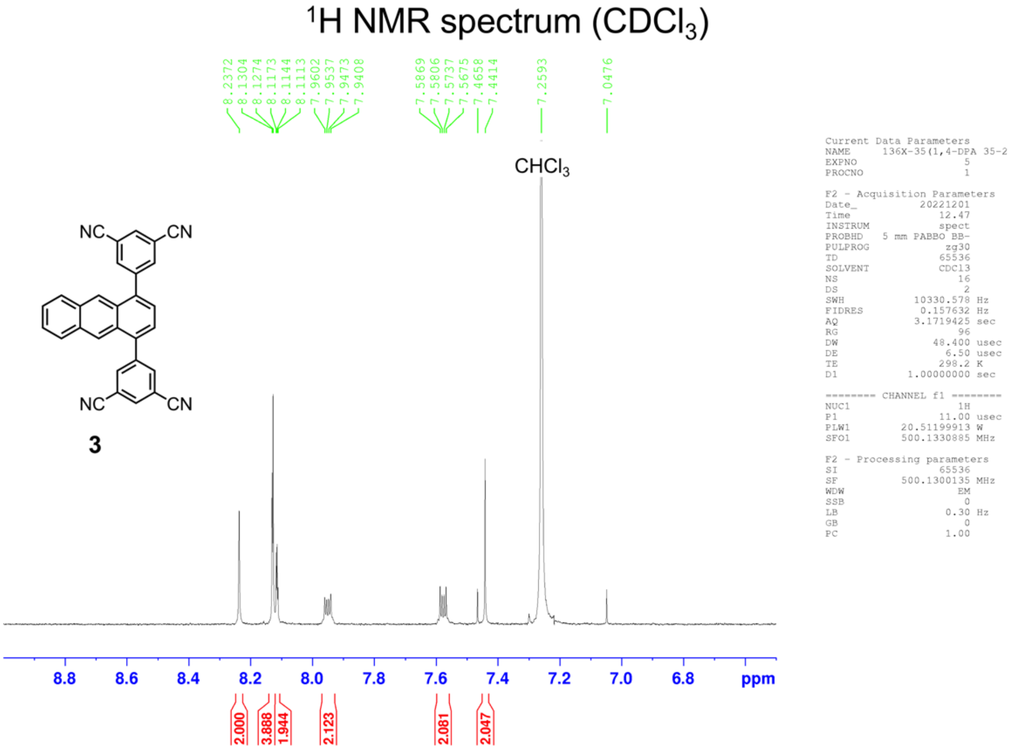

1H NMR spectrum (CDCl3)
8.2372 8.1304 8.1274 8.1173 8.1144 8.1113 7.9602 7.9537 7.9473 7.9408
7.5869 7.5806 7.5737 7.5675 7.4658 7.4414
7.2593
7.0476
CHCl3
NC
CN
NC
CN
3
8.8 8.6 8.4 8.2 8.0 7.8 7.6 7.4 7.2 7.0 6.8 ppm
2.000 3.888 1.944 2.123 2.081 2.047
Current Data Parameters
NAME 136X-35(1,4-DPA 35-2
EXPNO 5
PROCNO 1
F2 - Acquisition Parameters
Date_ 20221201
Time 12.47
INSTRUM spect
PROBHD 5 mm PABBO BB-
PULPROG zg30
TD 65536
SOLVENT CDCl3
NS 16
DS 2
SWH 10330.578 Hz
FIDRES 0.157632 Hz
AQ 3.1719425 sec
RG 96
DW 48.400 usec
DE 6.50 usec
TE 298.2 K
D1 1.00000000 sec
======== CHANNEL f1 ========
NUC1 1H
P1 11.00 usec
PLW1 20.51199913 W
SFO1 500.1330885 MHz
F2 - Processing parameters
SI 65536
SF 500.1300135 MHz
WDW EM
SSB 0
LB 0.30 Hz
GB 0
PC 1.00

^{13}C NMR spectrum ($CDCl_3$)

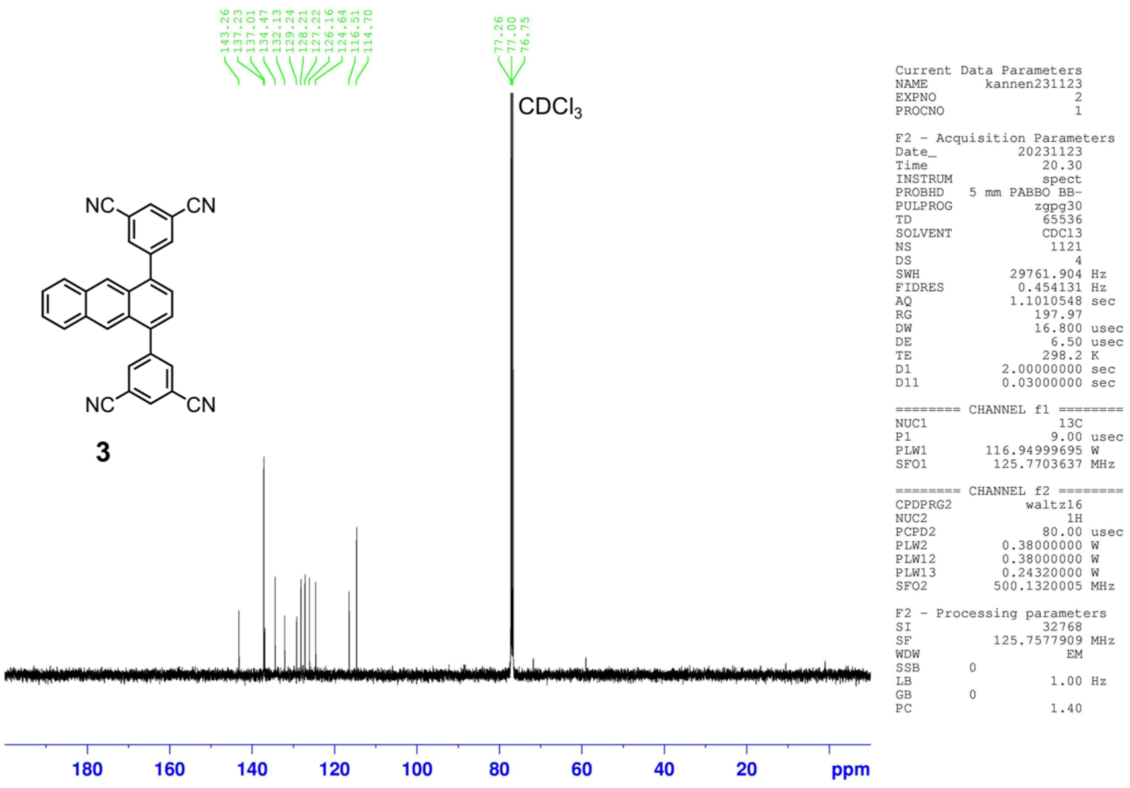

^{13}C NMR spectrum ($CDCl_3$)

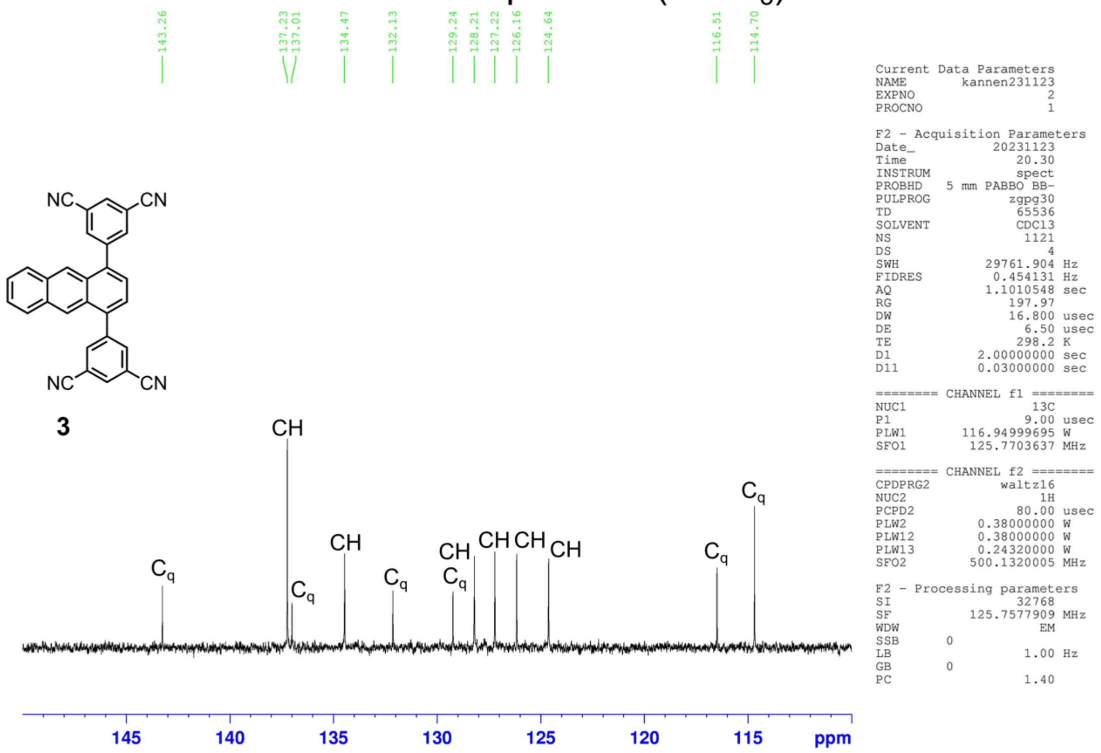

DEPT 135 NMR spectrum ($CDCl_3$)

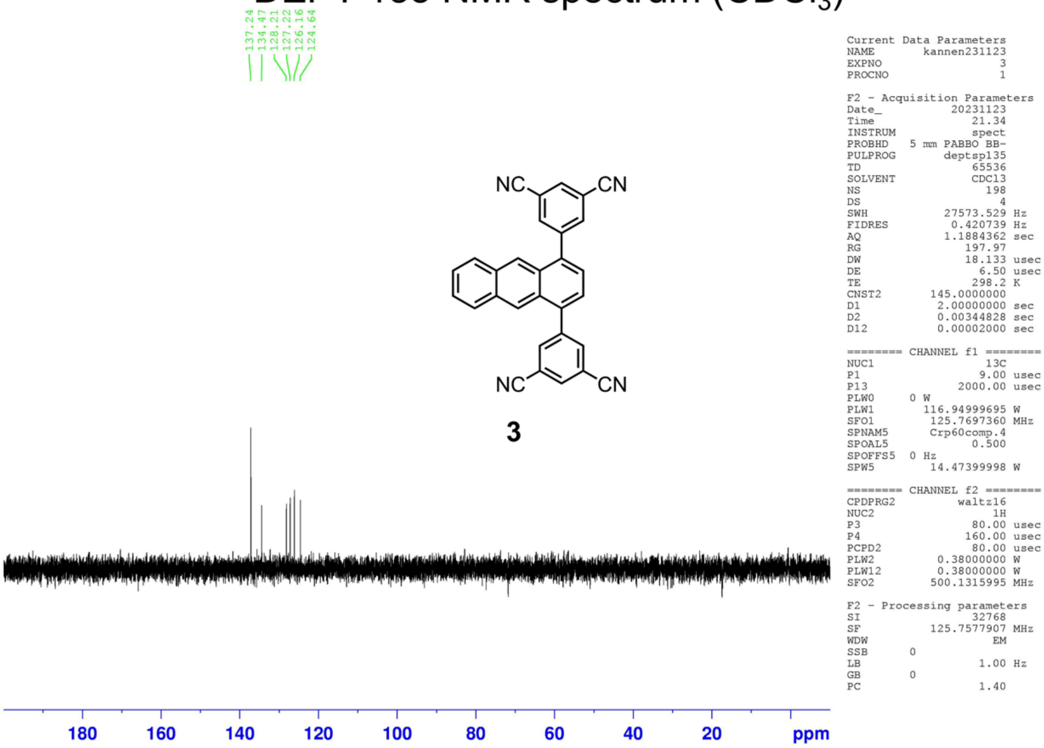

DEPT 135 NMR spectrum ($CDCl_3$)

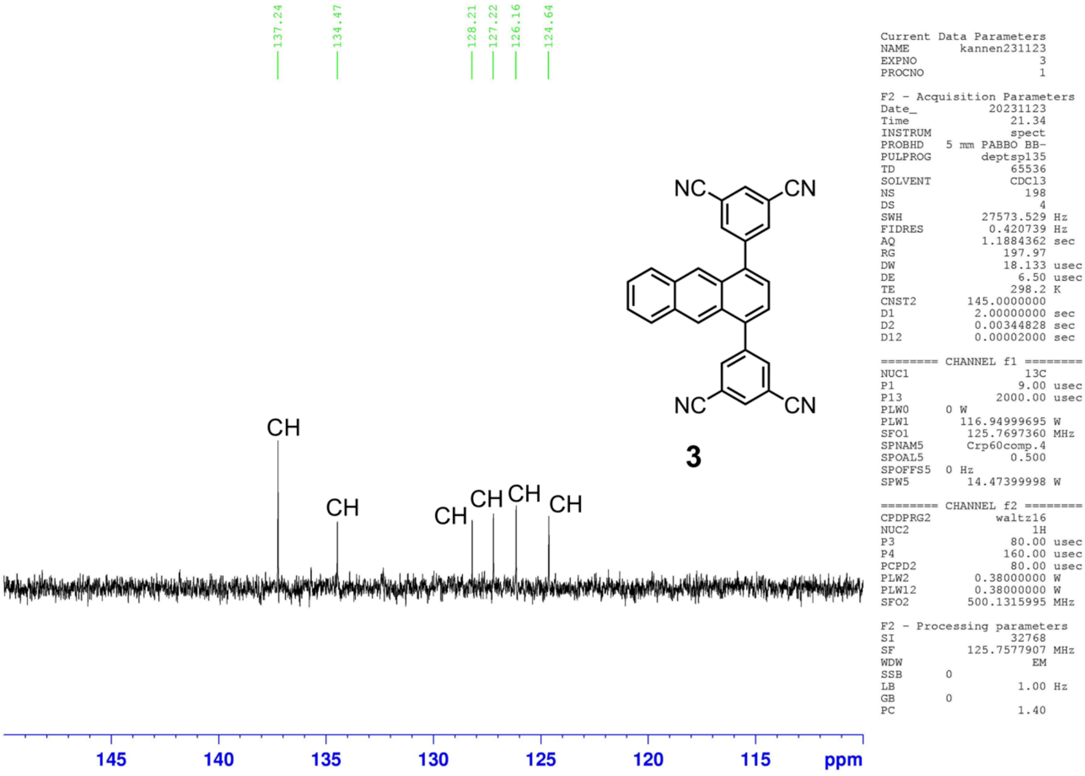

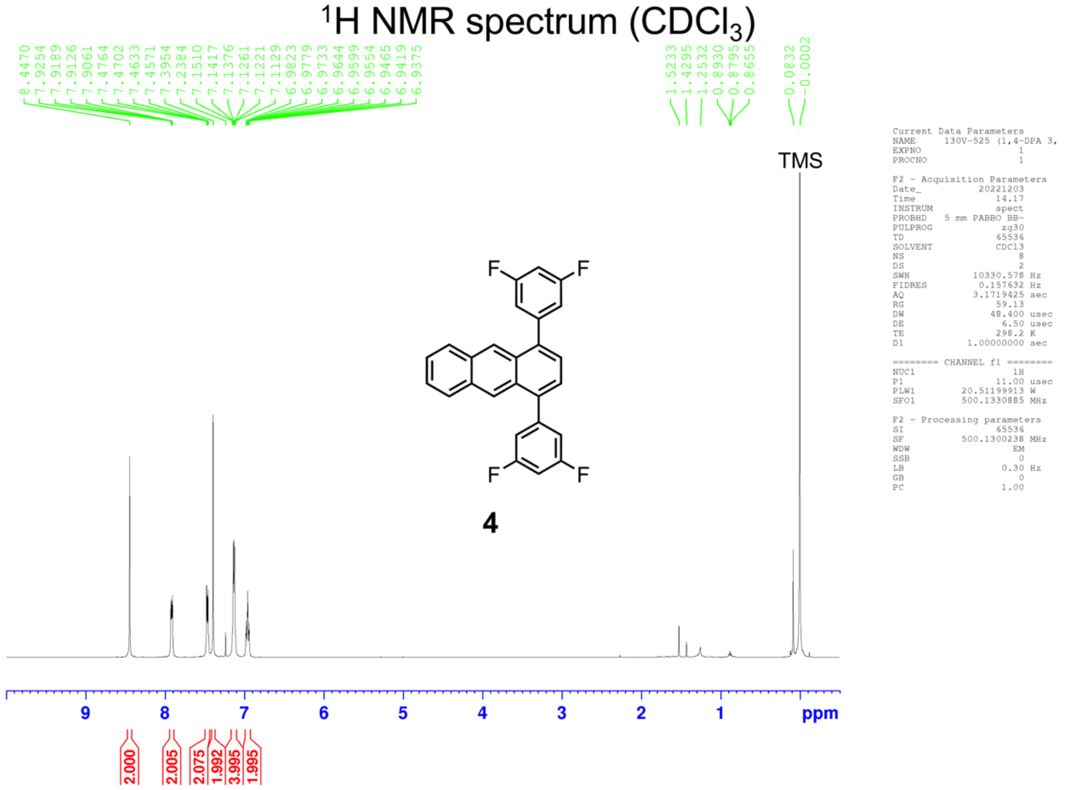
¹H NMR spectrum (CDCl3)
TMS
4
ppm

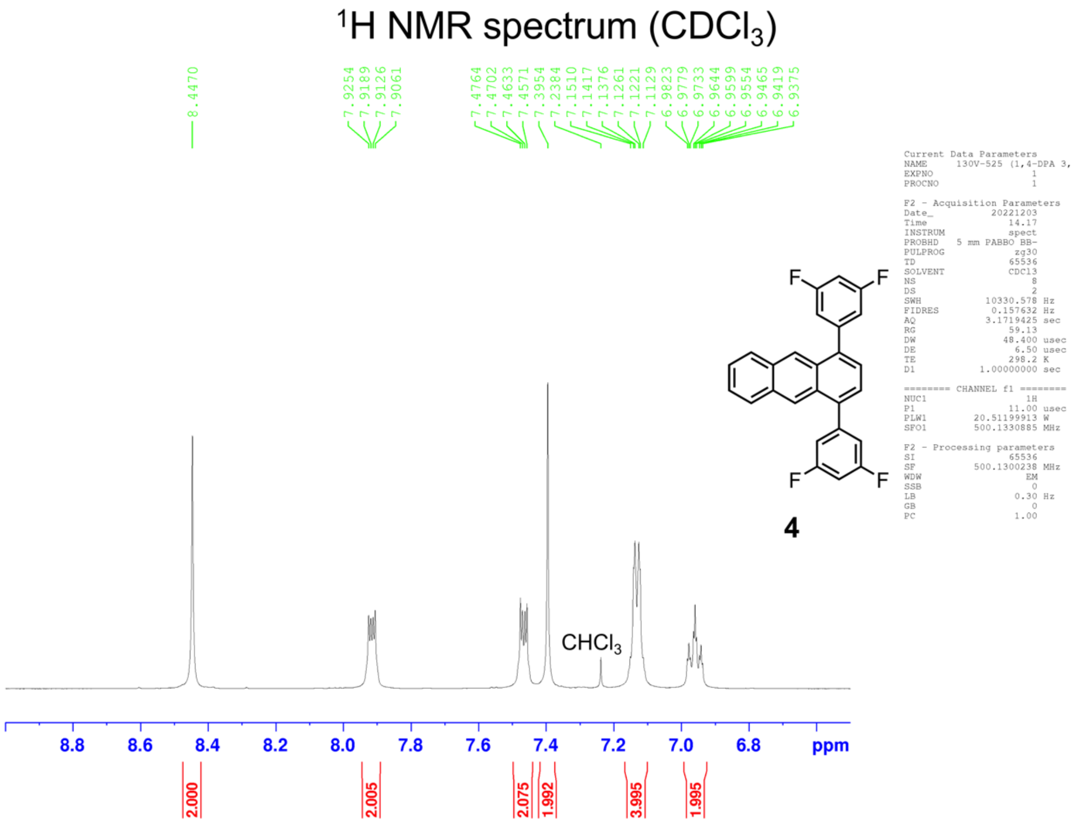
¹H NMR spectrum (CDCl3)
CHCl3
4
ppm

^{1}H NMR spectrum ($CDCl_3$)

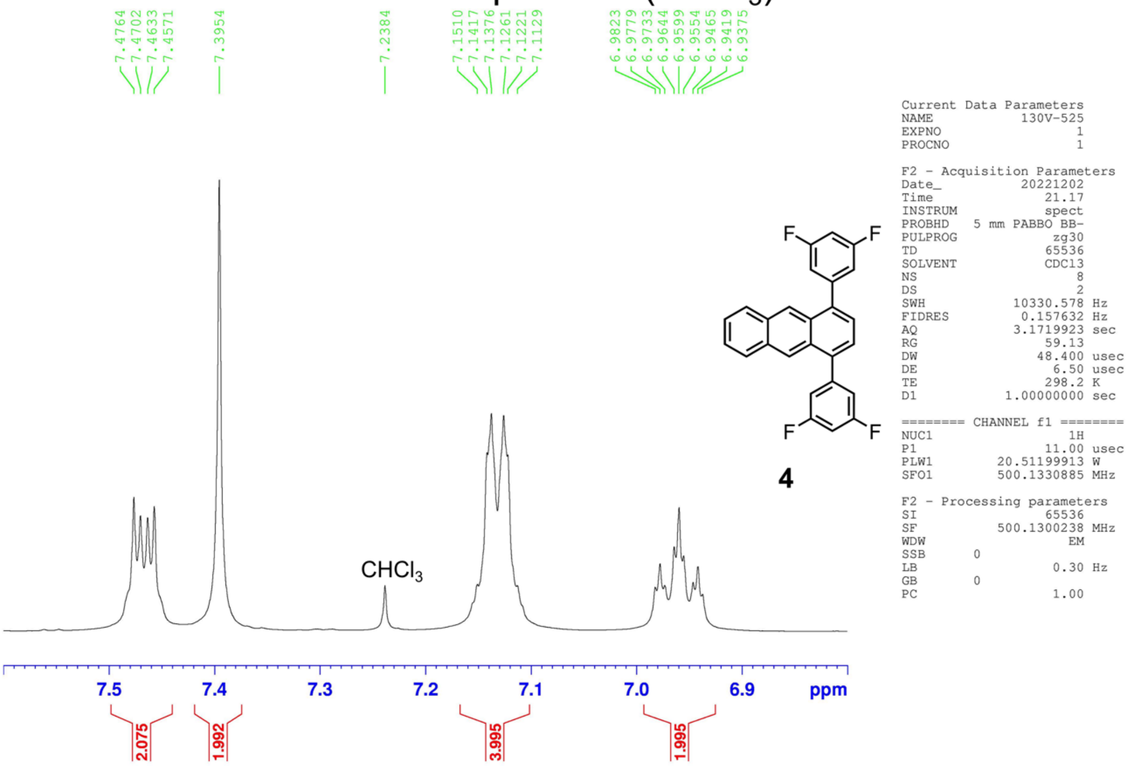

^{13}C NMR spectrum ($CDCl_3$)

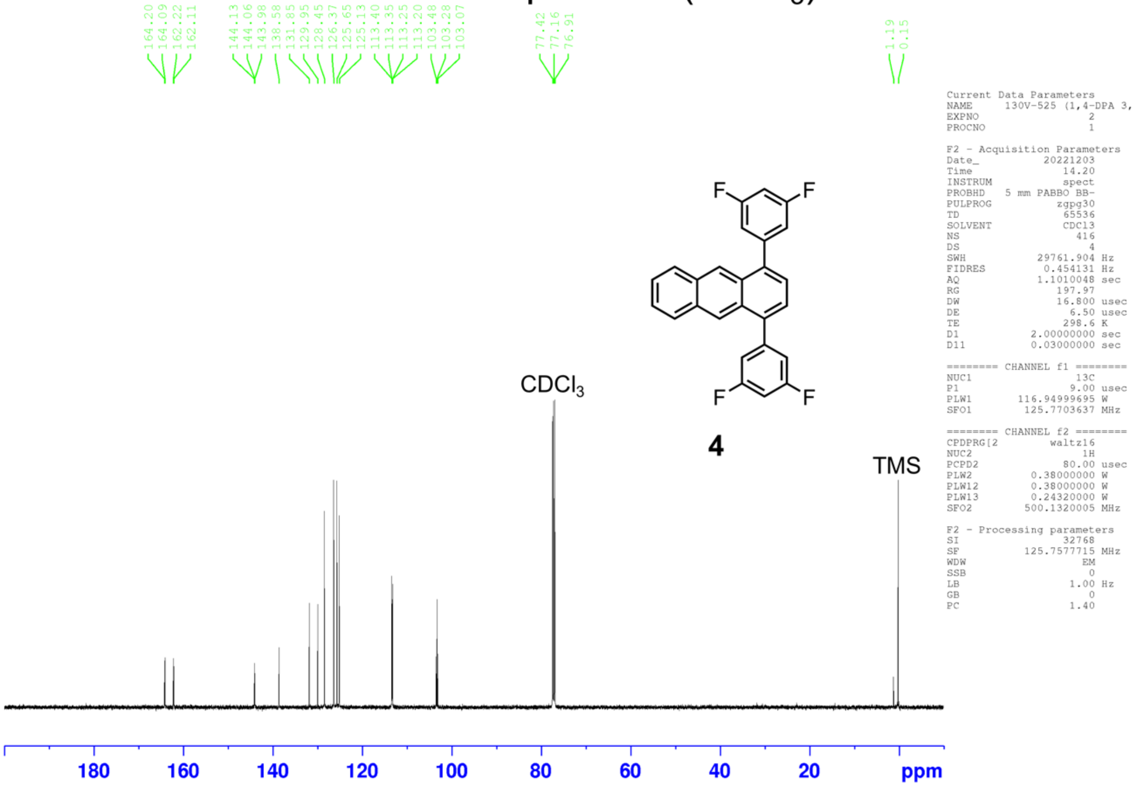

^{13}C NMR spectrum ($CDCl_3$)

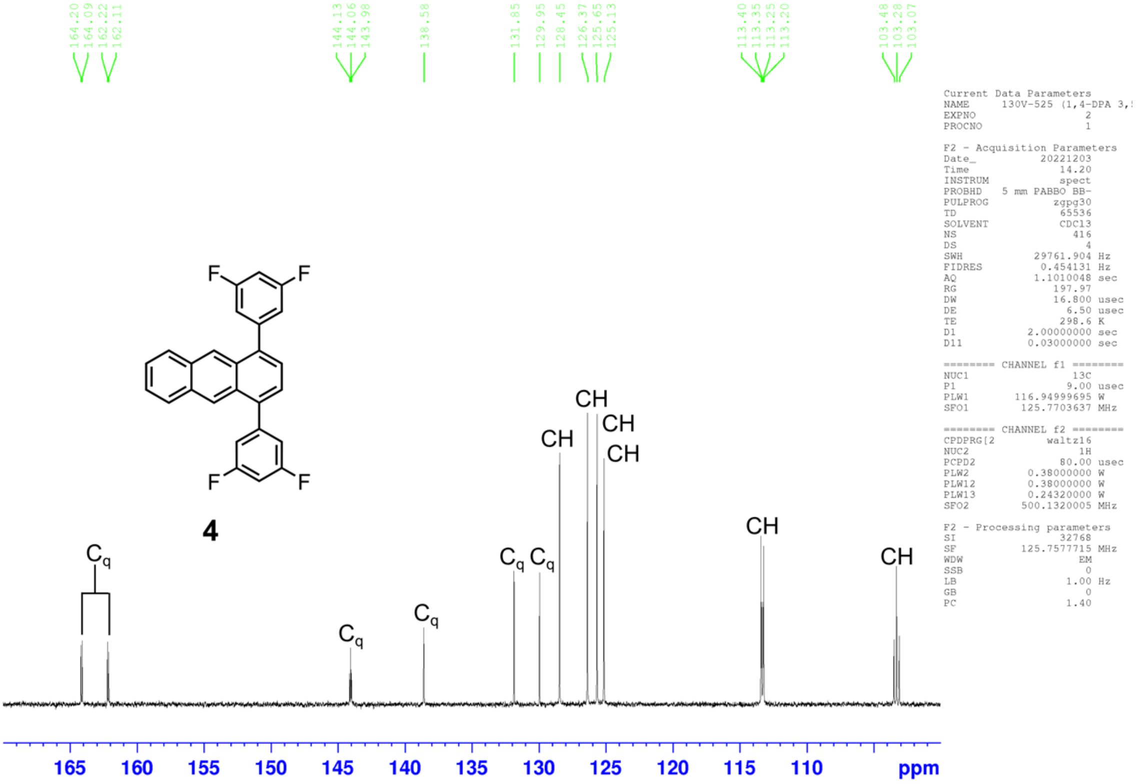

^{13}C NMR spectrum ($CDCl_3$)

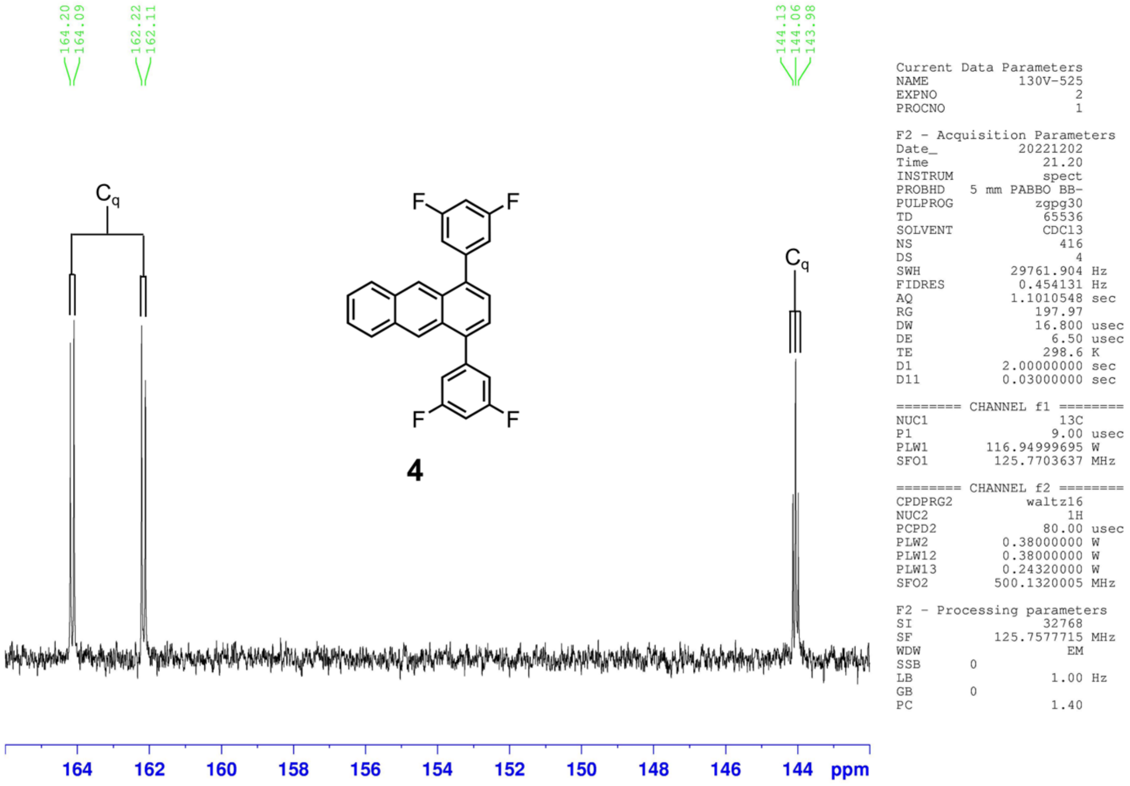

^{13}C NMR spectrum ($CDCl_3$)

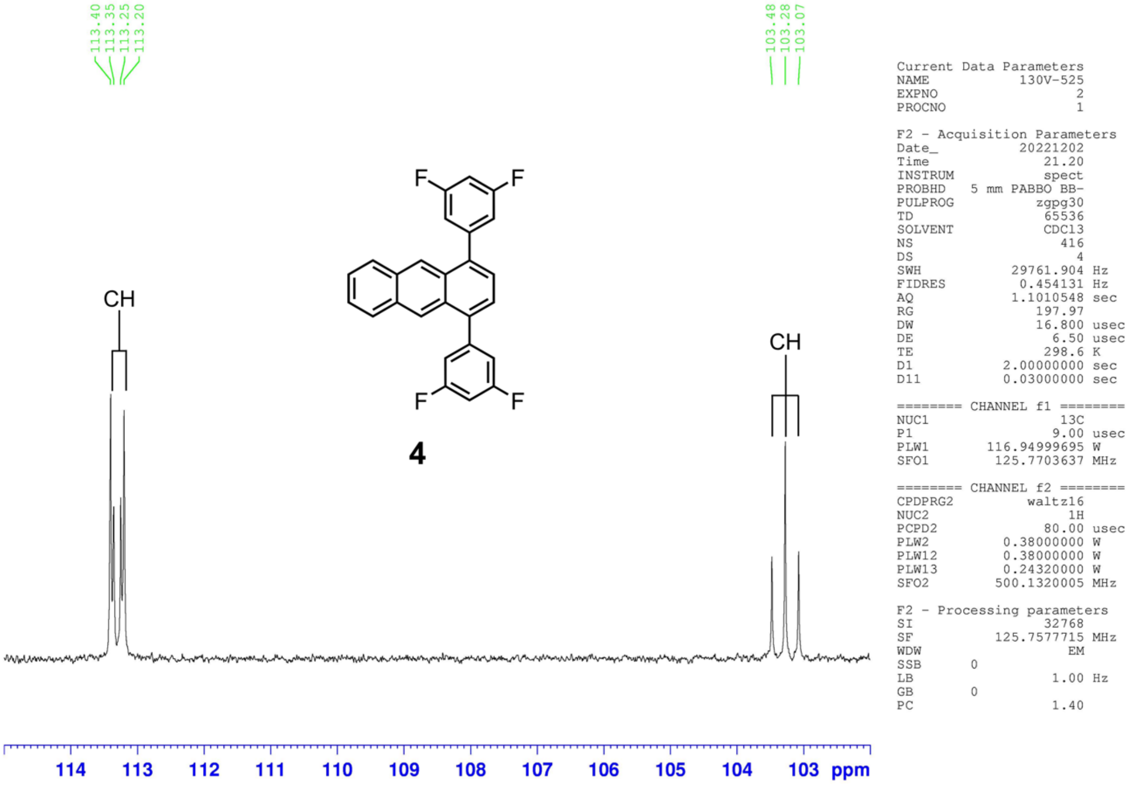

DEPT 135 NMR spectrum ($CDCl_3$)

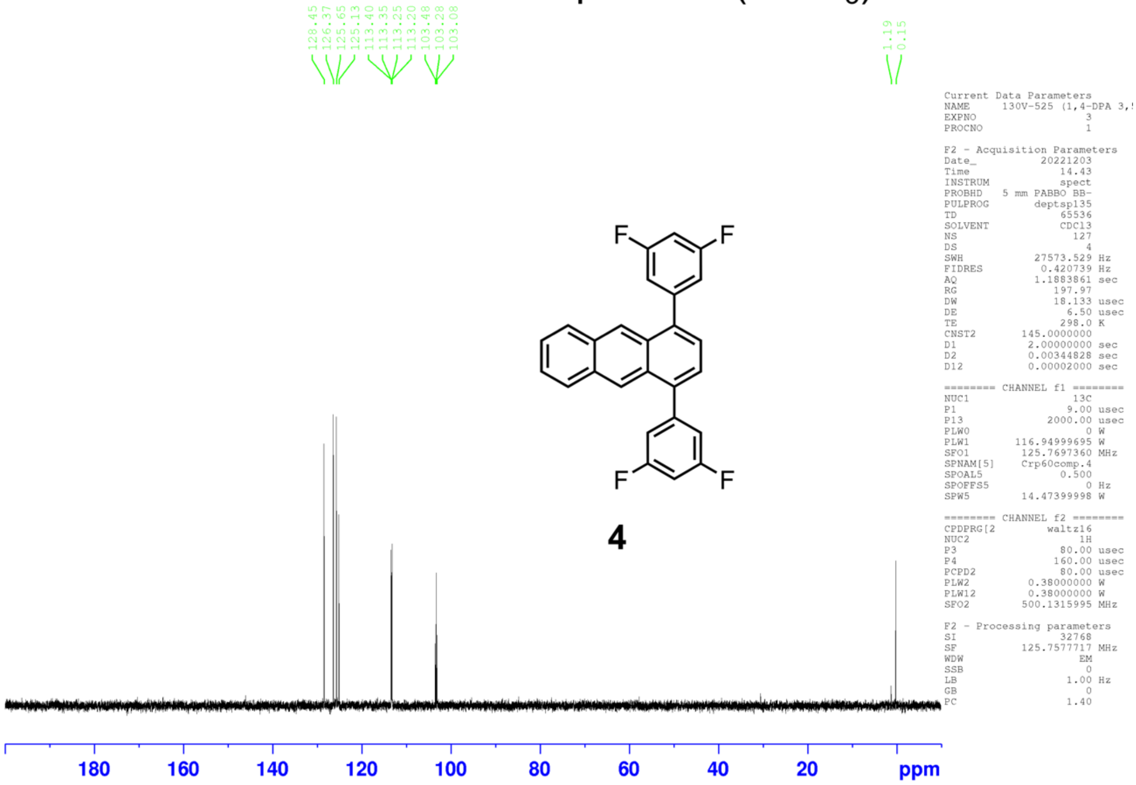

DEPT 135 NMR spectrum ($CDCl_3$)

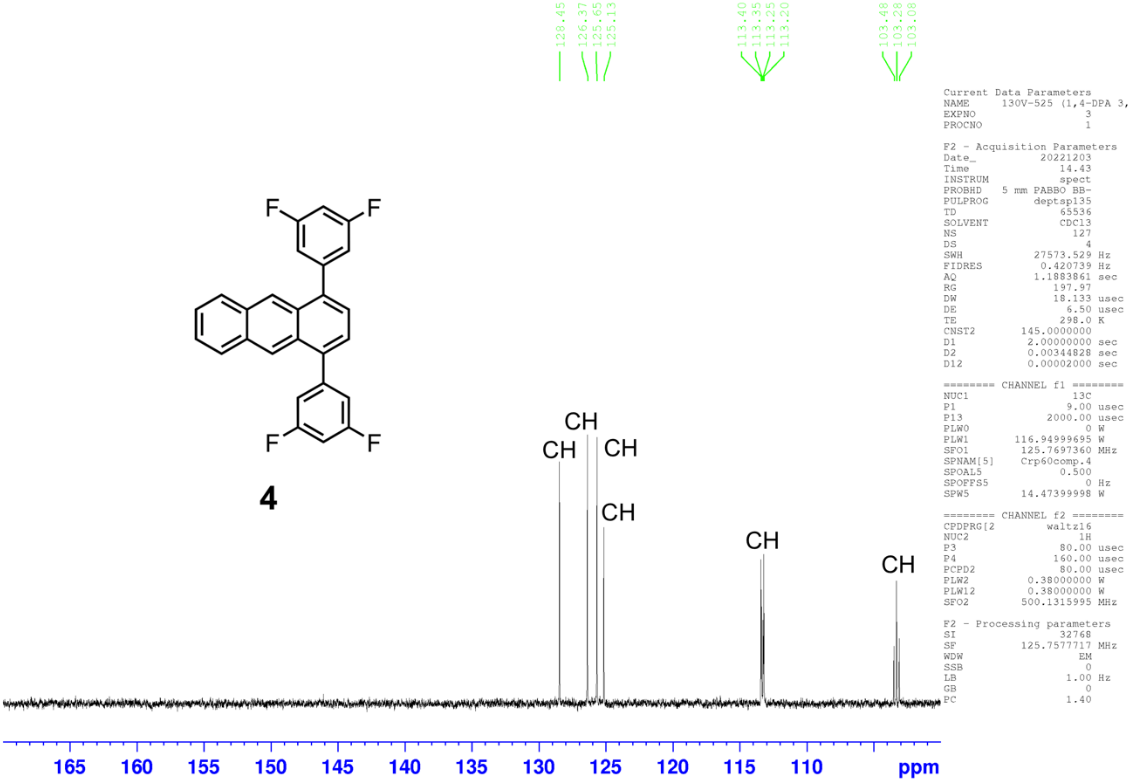

^{19}F NMR spectrum ($CDCl_3$)

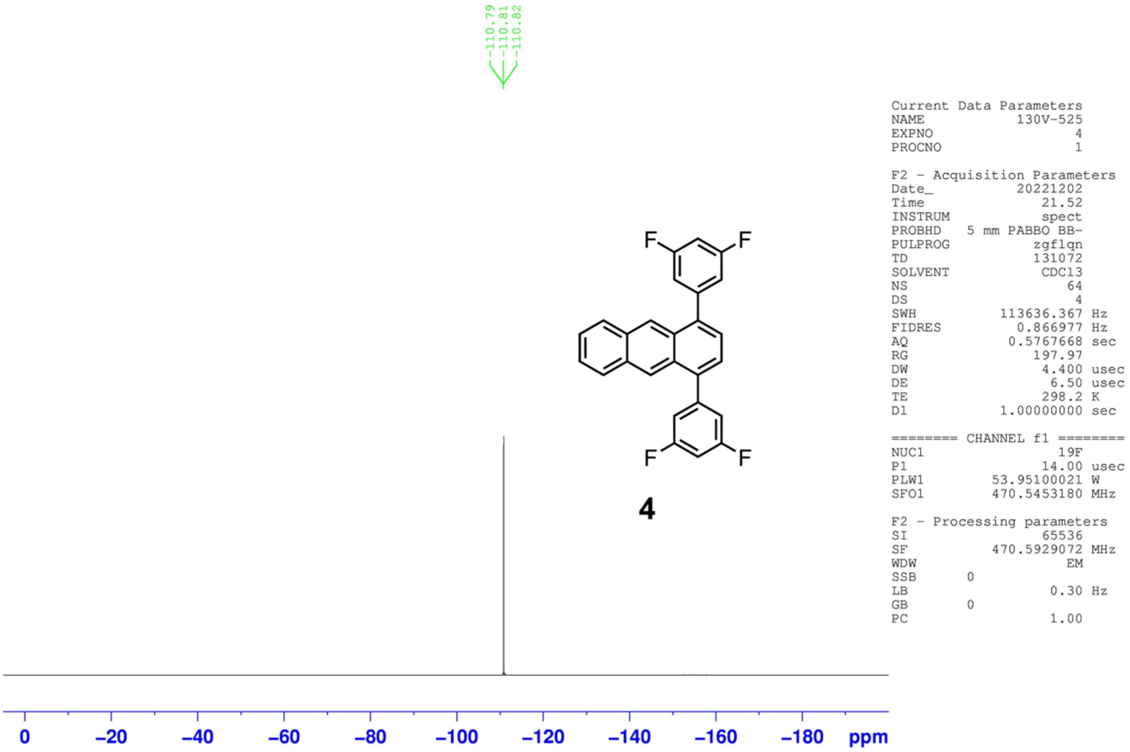

Supplement: Supplementary file 1 [file molecules-29-00407-s001.zip › molecules-2803845-supplementary.pdf]
